# An evolutionary model of sensitive periods when the reliability of cues varies across ontogeny

**DOI:** 10.1093/beheco/arab113

**Published:** 2021-10-25

**Authors:** Nicole Walasek, Willem E Frankenhuis, Karthik Panchanathan

**Affiliations:** 1 Behavioral Science Institute, Radboud University, Thomas van Aquinostraat 4, 6525 GD Nijmegen, the Netherlands; 2 Department of Psychology, Utrecht University, Heidelberglaan 1, 3584 CS Utrecht, the Netherlands; 3 Max Planck Institute for the Study of Crime, Security and Law, Günterstalstraße 73, 79100 Freiburg, Germany; 4 Department of Anthropology, University of Missouri, 225 Swallow Hall Columbia, MO 65211, USA

**Keywords:** cues, development, evolution, modeling, phenotypic plasticity, sensitive periods, stochastic dynamic programming

## Abstract

Sensitive periods are widespread in nature, but their evolution is not well understood. Recent mathematical modeling has illuminated the conditions favoring the evolution of sensitive periods early in ontogeny. However, sensitive periods also exist at later stages of ontogeny, such as adolescence. Here, we present a mathematical model that explores the conditions that favor sensitive periods at later developmental stages. In our model, organisms use environmental cues to incrementally construct a phenotype that matches their environment. Unlike in previous models, the reliability of cues varies across ontogeny. We use stochastic dynamic programming to compute optimal policies for a range of evolutionary ecologies and then simulate developmental trajectories to obtain mature phenotypes. We measure changes in plasticity across ontogeny using study paradigms inspired by empirical research: adoption and cross-fostering. Our results show that sensitive periods only evolve later in ontogeny if the reliability of cues increases across ontogeny. The onset, duration, and offset of sensitive periods—and the magnitude of plasticity—depend on the specific parameter settings. If the reliability of cues decreases across ontogeny, sensitive periods are favored only early in ontogeny. These results are robust across different paradigms suggesting that empirical findings might be comparable despite different experimental designs.

## Teaser

Phenotypic plasticity, the ability to tailor development to different conditions, typically declines with age. However, some traits have sensitive periods, windows of heightened plasticity, at later life stages, such as adolescence. The evolution of such windows is not well understood. Using mathematical modeling we find that changes in the reliability of information available to developing organisms may influence when sensitive periods evolve. Specifically, when the reliability of information increases across ontogeny, sensitive periods evolve later.

## INTRODUCTION

Sensitive periods are life stages during which experiences shape an organism’s phenotypic development to a greater extent than other stages (Bateson 1979; Fawcett and Frankenhuis 2015). While heightened phenotypic plasticity early in life appears to be the norm, it is by no means the rule. As with everything else in nature, the timing of sensitive periods varies. Sensitive periods may vary in their onset, duration, and offset across species, within species, and even among different traits within a single individual. Zebra finches learn their songs early in life, while European starlings are lifelong learners. Human children vary in the extent to which exposure to adversity affects maturation rate (Belsky and Pluess 2009; Del Giudice et al. 2011). And, for children adopted from harsh conditions into supportive ones, cognitive and emotional systems adjust at different rates (Tottenham et al. 2010; Zeanah et al. 2011). Decades of empirical research have advanced our understanding about the neurobiological bases of such variation in sensitive periods (Knudsen 2004; Creanza et al. 2016), so much so that it is possible in some cases to experimentally modify the timing and duration of sensitive periods, and even to “reopen” sensitive periods that had already closed, through physiological intervention (Takesian and Hensch 2013; Reh et al. 2020).

### Existing models of sensitive period evolution

The theory exploring the conditions in which natural selection favors the evolution of phenotypic plasticity is well developed and understood (Via et al. 1995; Pigliucci 2005; Chevin and Lande 2011; Lande 2014, 2019). Recently, formal modeling has focused on the timing of plasticity over the life course. These models explore the selection pressures that shape sensitive periods (reviewed in Fawcett and Frankenhuis 2015; Frankenhuis and Fraley 2017). More specifically, they explore how the impact of experience on phenotypic development varies across ontogeny.

A general result of models to date is that sensitive periods are typically only favored early in ontogeny. This result has been observed in a variety of scenarios, including when organisms integrate information inherited through genes (or epigenes) with individual experience (Stamps and Krishnan 2014, 2017), when organisms develop social behaviors such as helping (Kuijper and Johnstone 2019), when experiences simultaneously impact the phenotype (e.g. a nonlethal predator attack reducing somatic quality) and allow learning about the environment (e.g. updating estimates of predator density) (English et al. 2016), and when organisms build phenotypes incrementally rather than instantaneously (e.g. predator defenses in Daphnia; Whitman and Agrawal 2009) while sampling imperfect cues to the environmental state (Frankenhuis and Panchanathan 2011a; Panchanathan and Frankenhuis 2016). The duration of plasticity typically depends on the degree to which uncertainty about environmental conditions persists across ontogeny. Organisms that are able to reduce their uncertainty faster often lose plasticity earlier than organisms that remain uncertain.

To our knowledge, only two models have documented the evolution of sensitive periods later in ontogeny, that is, the highest levels of plasticity occurring halfway through ontogeny (Fischer et al. 2014; Stamps and Krishnan 2017). Both models assume that organisms start ontogeny with an induced phenotype and that development is fully reversible and unconstrainted, such that organisms can express any phenotype at any time during ontogeny. Although these models find that age-dependent declines in plasticity are favored across the majority of explored conditions, they also find that plasticity may first increase early in ontogeny before decreasing when there is a discrepancy between organisms’ inherited information and early-life experiences; that is, when these two sources of information indicate different states of the world. More generally, this discrepancy rule is said to cause small increases in plasticity early in ontogeny in Bayesian models of development, of which the Stamps and Krishnan (2017) model is one example (Fawcett and Frankenhuis 2015; Stamps and Frankenhuis 2016).

All models to date—that is, those that find sensitive periods early in ontogeny as well as those that find sensitive periods halfway through ontogeny—have assumed that the cue reliability is constant within the lifetime of an organism. It is unknown how this assumption affects their shared finding that sensitive periods are typically favored early in ontogeny, rarely halfway through ontogeny, and never at the end of ontogeny. In this paper, we present a mathematical model that explores the timing of sensitive periods favored by natural selection when the cue reliability varies across ontogeny.

### When are mid-ontogeny sensitive periods adaptive?

Though less common, sensitive periods in later developmental stages are widespread. In mammals, experiences during adolescence typically influence adult social behavior to a greater degree than experiences during childhood (Buwalda et al. 2011; Mutwill et al. 2020; Sachser et al. 2020). For example, adolescent guinea pigs housed in large colonies respond to being transferred to a new colony by developing lower levels of stress and aggression as adults, more so than juvenile guinea pigs do (Sachser et al. 2018). In humans, adolescence seems to be a period of enhanced plasticity in several neural and cognitive traits (Dahl 2004; Blakemore and Mills 2014; Fuhrmann et al. 2015; Knoll et al. 2016; Larsen and Luna 2018). For example, adolescents are more sensitive to the effects of social stress, such as social isolation, on mental health, and are more capable of recovering from those same social stressors compared to children and adults (Fuhrmann et al. 2015). Recent work suggests that adolescents, more so than children or adults, rely on learning strategies that are specifically suited to exploring novel opportunities and challenges in the environment (Raab and Hartley 2020).

Some researchers have speculated that natural selection might favor later sensitive periods when the reliability of cues varies across ontogeny (Fawcett and Frankenhuis 2015; Frankenhuis and Fraley 2017). Variation in cue reliability may arise when the information available to an organism systematically changes across ontogeny. Such a change may happen if organisms use the same cue across ontogeny, but its reliability changes across different developmental stages. Another possibility is that organisms receive cues more frequently at some developmental stages compared to others, and combining cues increases reliability (Fawcett and Johnstone 2003; Mariette 2020). A third possibility is that organisms use different cues, with different reliabilities, at different developmental stages. In all three scenarios, natural selection might have adapted organisms to anticipate changes in cue reliabilities across developmental stages.

We explore three patterns of cue reliability across ontogeny: “increasing”, “decreasing”, and “first increasing and then decreasing” (or “triangular”). Cue reliability might increase when an animal estimates its competitive ability in adulthood based on its interactions with conspecifics in the juvenile period. For example, during male-male combat animals often use an opponent’s relative body size to predict combat outcome and to adjust their behavior accordingly, such as whether to fight or not (McCullough and Simmons 2016; Li et al. 2018; Matsumura et al. 2020). As the animal and its conspecifics approach their adult form, relative body size becomes an increasingly reliable indicator of competitive ability in adulthood. Cue reliability might decrease when cues are more frequent, or only available, earlier in life. Prenatal cues, for example, may provide an integrative summary of the experiences of recent matrilineal ancestors, which predicts future nutritional conditions more reliably than early postnatal observations (Kuzawa 2005). Theoretically, it is also conceivable that cue reliability first increases and later decreases. Although examples of this pattern may be rarer in nature, we speculate that early adolescent social bonds in humans follow such a pattern. Adolescents form strong bonds with peers (Forbes and Dahl 2010). The feedback adolescents receive from these relationships might be more informative about their social status or mate value in adulthood than feedback received in early childhood or right before the onset of adulthood (Forbes and Dahl 2010; Allen et al. 2014). We do not explore the cue reliability “first decreasing and later increasing”. This pattern has not been proposed in the literature nor are we aware of empirical examples in nature.

### Our contribution

Here, we develop a model in which organisms sample environmental cues and tailor their phenotypes to the environmental state. Phenotypic development is both incremental and irreversible, in the sense that organisms gradually adjust phenotypes and that developed adjustments cannot be undone. Extending previous work (Frankenhuis and Panchanathan 2011a; Panchanathan and Frankenhuis 2016), we introduce variation in cue reliability across ontogeny. We use stochastic dynamic programming to compute optimal developmental policies across a range of evolutionary ecologies. Such a “policy” prescribes the optimal developmental decision given the organism’s state, which comprises the current phenotype and the environmental cues sampled thus far. The optimal policy maximizes expected fitness at the end of ontogeny. We then examine these optimal developmental policies to extract information about the patterning of phenotypic plasticity across ontogeny. In particular, we hope to better understand when natural selection favors the later emergence of sensitive periods.

We also examine how phenotypic variation develops among organisms who follow the same optimal policy. Previous models have shown that individual differences in phenotypes tend to stabilize across ontogeny (Frankenhuis and Panchanathan 2011a; Panchanathan and Frankenhuis 2016), but have not quantified this process. To this end, we develop a measure of trait repeatability. Repeatability is widely used in studies of animal personality to quantify consistency in individual differences over time (Roberts and DelVecchio 2000; Fisher et al. 2018; Trillmich et al. 2018; Kok et al. 2019; Polverino et al. 2019).

Finally, we examine the robustness of our findings by conducting two kinds of sensitivity analyses. First, we quantify patterns of plasticity across ontogeny using paradigms commonly used in empirical research. This approach links theoretical and empirical research: it allows us to compare qualitative predictions from different empirical paradigms. Second, we investigate differences in patterns of plasticity as a result of simplifying the model. Some models have incorporated phenotype as a fitness determinant (e.g. Panchanathan and Frankenhuis 2016), and others only the information state of an organism (e.g. Stamps and Krishnan 2014, 2017). By comparing these models, we can explore to what extent our qualitative results generalize across models; whether a complex model structure that includes phenotypes alongside information states offers any insights that cannot be obtained from an information-only model; and how information state, both on its own and in combination with phenotype, affects the evolution of mid-ontogeny sensitive periods.

## MATERIALS AND METHODS

### The environment and the organism

Organisms are born and randomly disperse into discrete and non-overlapping patches which can be in one of two states: *E*_0_ or *E*_1_ (e.g. dangerous or safe). The state of a patch does not change over ontogeny. Organisms sample environmental cues and develop phenotypes, reproduce proportional to fitness, and die. We assume that organisms have adapted to the fixed distribution of patches in the environment (McNamara et al. 2006), and use this distribution at the onset of ontogeny as a prior estimate about the probability of being in one state or the other.

Ontogeny consists of *T* = 20 discrete time periods. Organisms can develop towards two phenotypic targets, *P*_0_ and *P*_1_ which correspond to the optimal, fully specialized phenotypes for *E*_0_ and *E*_1_. Increments toward each of these two phenotypic targets occur on independent dimensions; these two phenotypes are not endpoints of a single and continuous trait (for similar models, see Frankenhuis and Panchanathan 2011a; Panchanathan and Frankenhuis 2016). For example, we might imagine that an organism can invest in a heavily armored phenotype to avoid predation or, instead, invest in a heavily adorned phenotype to attract mates. We track increments towards these targets with two numbers: the number of time periods specialized towards *P*_0_ (denoted by *y*_0_) and towards *P*_1_ (denoted by *y*_1_). At the onset of ontogeny organisms start with 0 specializations towards either phenotypic target (*y*_0_ = *y*_1_ = 0). In each time period, organisms receive an environmental cue and then either increment *y*_0_ by 1, increment *y*_1_ by 1, or wait and forgo specialization in this time period (leaving *y*_0_ and *y*_1_ unchanged). We denote the number of time periods waited by *y*_*w*_.

Development is irreversible in the sense that once a phenotypic increment has developed, it cannot be undone. However, organisms can switch developmental trajectories and specialize towards the other phenotypic target, for instance, because they have revised their estimates. At the end of ontogeny, the number of increments towards *P*_0_ and *P*_1_ and the number of time periods waited sum to the total number of time periods (*y*_0_ + *y*_1_ + *y*_*w*_ = *T*). In this way, phenotypic development is constrained by the duration of ontogeny. The later organisms start specializing towards one of the phenotypic targets, the fewer increments they can make towards it.

Environmental cues provide informative but imperfect guidance. The reliability of a cue indicates the probability of receiving the current cue (*C*_0_ or *C*_1_) conditioned on being in the corresponding environmental state (*E*_0_ or *E*_1_). We assume that organisms “know” the reliability of a cue because they have adapted to the association between cues and environmental states over evolutionary time. However, because the reliability of cues varies across ontogeny, we denote the cue reliabilities of *C*_0_ and *C*_1_ at time *t* as *P*(*C*_0,*t*_|*E*_0_) and *P*(*C*_1,*t*_|*E*_1_). The probabilities of observing an incorrect cue then correspond to: *P*(*C*_1,*t*_|*E*_0_) = 1 − *P*(*C*_0,*t*_|*E*_0_) and *P*(*C*_0,*t*_|*E*_1_) = 1 − *P*(*C*_1,*t*_|*E*_1_). We assume that the cue reliability is the same in both environmental states, i.e. *P*(*C*_0,*t*_|*E*_0_) = *P*(*C*_1,*t*_|*E*_1_).

Over time, organisms build up a dataset comprising the cues that they have sampled. We denote the sequence of cues until time period *t* by *D*_*t*_ = {*x*_1_, *x*_2_, … *x*_*t*_,}, where *x*_1_, *x*_2_, etc. until *x*_*t*_ denote the kind of cue (*C*_0_ or *C*_1_) received in each time period. At any given time *t*, the state of an organism comprises the developmental decisions it has made and the environmental cues it has received, denoted by the tuple (*D*_*t*_,*y*_0_,*y*_1_,*y*_*w*_,*t*).

We consider three patterns of cue reliability across ontogeny: (1) linearly increasing, (2) linearly decreasing, and (3) first linearly increasing and then linearly decreasing (triangular). All three patterns range between a minimum cue reliability of 0.55 and a maximum cue reliability of 0.95. We ensure that the average cue reliability across ontogeny is the same across cue reliability patterns. This controls for the total information available to organisms across all of ontogeny. To explore whether results are driven by the maximally attainable cue reliability, we also computed results for patterns ranging between 0.55 and 0.75 (see Supplementary Material 1, Figure A1.1). Results from both ranges were qualitatively similar, so we report only the range 0.55 to 0.95 in the main text.

We assume that organisms are Bayesian learners (McNamara and Houston 1980; Mangel 1990; Tufto 2000; McNamara et al. 2006; Trimmer et al. 2011; Dall et al. 2015; Stamps and Frankenhuis 2016), using the fixed distribution of patches as the prior estimate of the environmental state and the time-dependent cue reliabilities to update these estimates (Stamps and Frankenhuis 2016). To see how this works, suppose an organism has sampled a specific sequence of cues *D*_*t*=3_ = {*C*_0_,*C*_1_,*C*_0_}.

According to Bayes’ theorem, its posterior estimate after the first cue is:


$$\begin{array}{l} \begin{array}{ll} & P\left(E_{0}\;|\;C_{0}\right)=\;\frac{P\left(C_{0}|E_{0}\right)•P\left(E_{0}\right)}{P\left(C_{0}|E_{0}\right)•P\left(E_{0}\right)+\;P\left(C_{0}|E_{1}\right)•P\left(E_{1}\right)}\\ \left[1pt\right] & P\left(E_{1}\;|\;C_{0}\right)=1-\;P\left(E_{0}\;|\;C_{0}\right) \end{array} \end{array}$$ (1)

To compute the posteriors *P*(*E*_0_|*D*_*t*_) and *P*(*E*_1_|*D*_*t*_) after the whole sequence of cues, we have to reapply Bayes’ theorem for each cue using the previous posterior as the new prior. We provide an overview of our variables and the Bayesian inference in Supplementary Material 2 (sections a and b). Additionally, we depict which posterior estimates result from different cue reliability patterns and priors in Supplementary Material 3.

### Mapping from phenotype to fitness

We assume that fitness is accrued at the end of ontogeny (e.g. adulthood). A mature organism accrues fitness depending on how well its phenotype matches the environmental state. The better the match, the higher the fitness. Therefore, the earlier an organism specializes, the more it can improve its fit with the environment (Panchanathan and Frankenhuis 2016). In this way, there is an opportunity cost to delaying phenotypic specialization (Dunlap and Stephens 2016). In addition, we assume that developing a phenotype that does not match the environmental state reduces fitness, and the penalty magnitude depends on the degree of mismatch (Innes-Gold et al. 2019). We do not, however, assume a constitutive cost of plasticity in the sense that there is no explicit cost for building, running, and maintaining the physiological mechanisms enabling plasticity (DeWitt et al. 1998; Relyea 2002; Auld et al. 2009). Nor do we assume a “switch cost” if organisms switch from specializing from one phenotypic target to another.

Equations (2)–(4) show the mapping of phenotypic increments to fitness rewards and penalties at the end of ontogeny (see also Supplementary Material 2, section c). We denote the mature phenotype at the end of ontogeny by *Y*_*mat*_ = (*y*_0_,*y*_1_,*T*). The parameter *π*_0_ corresponds to the baseline fitness of an organism that waited throughout ontogeny, never specializing toward either phenotypic target. The expression *ϕ*(*Y*_*mat*_) corresponds to the fitness reward for correct phenotypic specializations. The expression *ψ*(*Y*_*mat*_) corresponds to the fitness penalty for incorrect specializations. Thus total fitness, *π*(*Y*_*mat*_), is:


$$\begin{array}{l} \pi \left(Y_{mat}\right)=\;\pi_{0}+\phi \left(Y_{mat}\right)+\;\psi \left(Y_{mat}\right) \end{array}$$ (2)

We explore three mappings between phenotypic increments and fitness effects. With “linear” fitness effects, each correct (or incorrect) increment results in a constant marginal fitness gain (or loss). With “decreasing” fitness effects, the marginal fitness gain (or loss) of each correct (or incorrect) increment decreases. And with “increasing” fitness effects, the marginal fitness gain (or loss) of each correct (or incorrect) increment increases. The formulas for these mappings can be found in Supplementary Material 2.c. The attainable fitness payoff for a perfectly matched organism is the same for each fitness mapping and for each environmental state.

To see how these mappings work, suppose that an organism has sampled a specific sequence of cues, *D*_*t*_, throughout ontogeny. Its posterior estimates *P*(*E*_0_|*D*_*t*=*T*_) and *P*(*E*_1_|*D*_*t*=*T*_) reflect the probabilities of being in either environmental state at the end of ontogeny. Thus, to compute rewards and penalties, we need to compute the expectation across both environmental states, weighted by how likely each state is, as indicated by the posterior estimates at the end of ontogeny. We denote the mapping from phenotypic increments to rewards and penalties by *f*(*y*), where *y* can refer to both *y*_0_ and *y*_1_, and derive the following expressions for expected rewards and penalties:


$$\begin{array}{l} \begin{array}{ll} & \phi \left(Y_{mat}\right)=\;P\left(E_{0}\;|\;D_{t=T}\right)•f\left(y_{0}\right)+\;P\left(E_{1}\;|\;D_{t=T}\right)•f\left(y_{1}\right)\\ \left[1pt\right] & \psi \left(Y_{mat}\right)=-\left(P\left(E_{0}\;|\;D_{t=T}\right)•f\left(y_{1}\right)+\;P\left(E_{1}\;|\;D_{t=T}\right)•f\left(y_{0}\right)\right) \end{array} \end{array}$$ (3)

Inserting this into equation (2) results in the final formula for total fitness at the end of ontogeny:


$$\begin{array}{l} \begin{array}{ll} & \pi \left(Y_{mat}\right)=\;\pi_{0}+P\left(E_{0}\;|\;D_{t=T}\right)•f\left(y_{0}\right)+\;P\left(E_{1}\;|\;D_{t=T}\right)•f\left(y_{1}\right)\;\\ \left[1pt\right] & \;-\left(P\left(E_{0}\;|\;D_{t=T}\right)•f\left(y_{1}\right)+\;P\left(E_{1}\;|\;D_{t=T}\right)•f\left(y_{0}\right)\right) \end{array} \end{array}$$ (4)

### Optimal developmental policies

We use stochastic dynamic programming to compute optimal developmental policies for different evolutionary ecologies (McNamara and Houston 1980; Mangel and Clark 1988). We explore three prior distributions of environmental states: *P*(*E*_0_) = *P*(*E*_1_) = 0.5, *P*(*E*_0_) = 0.3 and *P*(*E*_1_) = 0.7, and *P*(*E*_0_) = 0.1 and *P*(*E*_1_) = 0.9; and three cue reliability patterns: increasing, decreasing, and triangular. For each possible state of an organism (*D*_*t*_, *y*_0_, *y*_1_, *y*_*w*_, *t*), stochastic dynamic programming identifies the developmental decision that will result in the highest expected fitness at the end of ontogeny. In the event of a tie between two or more options in a particular state, the organism chooses amongst the current alternatives with equal probability. *F*(*D*_*t*_, *y*_0_, *y*_1_, *y*_*w*_, *t*, *T*) denotes the maximum expected fitness that can be attained as a result of decisions made between *t* and *T*. The organism chooses option *a* to maximize expected fitness:


$$\begin{array}{l} \begin{array}{lll} & F\left(D_{t},y_{0},y_{1},y_{w},\;t,T\right)\;\; & =\underset{a\epsilon \left\{0,1,w\right\}}{\max}\;F_{a},\;where\\ \left[1pt\right] & \;F_{0} & =\;F\left(D_{t},y_{0}+1,y_{1},y_{w},\;t+1,T\right),\\ \left[1pt\right] & \;F_{1} & =\;F\left(D_{t},y_{0},\;y_{1}+1,y_{w},\;t+1,T\right),\\ \left[1pt\right] & \;F_{w} & =\;F\left(D_{t},y_{0},y_{1},y_{w}+1,\;t+1,T\right). \end{array} \end{array}$$ (5)

For each possible state of an organism, we initialize *F*(*D*_*t*_, *y*_0_, *y*_1_, *y*_*w*_, *T*, *T*), which represents the fitness at the end of ontogeny, with *π*(*Y*_*mat*_) as defined in equation (4). Using this as a starting point, we solve equation (5) via backwards induction. We also describe our approach to computing optimal policies in Supplementary Material 2 (section d). Our code, written in Python 2.7, is available on GitHub (Walasek 2021).

### Quantifying plasticity

We use a simulated “twin” study to quantify trajectories of plasticity across ontogeny. We first simulate 10 000 pairs of twins with identical phenotypes and posteriors following the optimal policy up to time period *t*. From each pair, we keep one twin in its natal patch (the focal individual) and move the other one into a “mirror” patch (the clone). From the time of separation until the end of ontogeny, the focal individual and the clone receive opposite environmental cues. That is, whenever the focal individual receives a cue indicating *E*_0_, the clone receives a cue indicating *E*_1_, and vice versa.

We then compare the mature phenotypes of twin pairs at the end of ontogeny. We define plasticity as the Euclidean distance between the two twins along the two phenotypic dimensions (*y*_0_ and *y*_1_). The larger the difference between mature twins, the more cues have shaped their phenotypes since their separation; thus, the more developmentally plastic these twins were at the time of their separation. Our paradigm resembles twin studies that compare similarities and differences between adult twins who were separated at different points in ontogeny to assess the impact of genetic and environmental factors on phenotypic development.

We distinguish between two measures of plasticity, “absolute” and “proportional”. Absolute plasticity is the average Euclidean distance across simulated twin pairs normalized to range between 0 and 1. Proportional plasticity is the average Euclidean distance between simulated twins, divided by the maximally achievable Euclidean distance from the moment of separation until the end of ontogeny. In contrast to absolute distance, proportional distance accounts for potential phenotypic distance, which is smaller the later separation occurs, as there is less time for twins to diverge. We offer both measures to facilitate comparison across different theoretical models and/or empirical studies that potentially use one or the other (or both) measures to quantify plasticity.

### Quantifying rank-order stability

We developed a measure of trait repeatability to quantify the process by which individual differences in phenotypes develop and might stabilize over ontogeny. We assume that organisms within a population that show high trait repeatability also show stable phenotype ranks across ontogeny. Specifically, we assume that the higher trait repeatability is, the lower the likelihood of rank-switches between two time periods. We simulate a population of 10 000 organisms and rank them at each time point during ontogeny based on their phenotypic values. At each time period, we compute the proportion of individuals that experiences a rank-switch (relative to the population size, which is constant) from one time period to the next. Organisms that have the same trait value share a rank. This paradigm allows us to compute not only the proportion of rank-switches between consecutive time periods, but also that between periods farther apart.

### Sensitivity analyses

**Figure 1:**
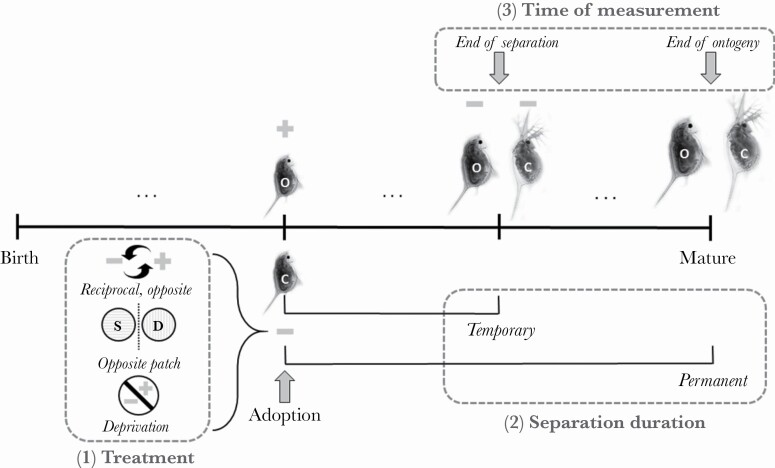
**Measuring changes in plasticity across ontogeny. We separate twins (original, denoted with O, and clone, denoted with C) at different ages. We vary three dimensions: treatment, separation duration, and time of measurement. (1) Treatment refers to how the experiences of the original and clone differ during their separation. The clone might experience reciprocal opposite cues; cues from the opposite patch; or deprivation. With reciprocal opposite cues, the clone always samples the opposite cue of the original: if the original samples a minus cue [–], the clone samples a plus cue [+]. With cues from the opposite patch, the clone samples a sequence of cues typical of the opposite patch: if the original tends to sample more minus cues, the clone tends to sample more plus cues. In our figure, the original and the clone are both in the dangerous patch (denoted with D), but the clone receives cues typical of the safe patch (denoted with S). With deprivation, the clone is equally likely to sample a plus or a minus cue; thus preventing learning about the environment. (2) Separation duration refers to whether the separation of twins is permanent or temporary. Permanent separation occurs if twins experience different conditions from their separation until the end of ontogeny (maturity). Temporary separation occurs if twins are reunited before the end of ontogeny. (3) Time of measurement refers to when differences in the phenotypes of twins are measured. We measure differences in phenotypes of twins at two different time points: at the end of their separation and at the end of ontogeny. Our results show that different treatments tend to produce (qualitatively) similar patterns of plasticity. Our predictions are therefore similar for different treatments and for different measurement times used in empirical research. Copyright: this figure has been adapted from** Frankenhuis and Walasek (2020) **and we have used the images of Daphnia with permission from Dr. Weiss (2019).**

We conduct two different kinds of sensitivity analyses. First, we explore whether results are robust to variations in the basic twin study paradigm. Specifically, we vary (1) the degree to which cues sampled by the separated clone differ from those sampled by its identical twin; (2) whether separation is temporary, lasting only for a fixed number of time periods, or permanent until the end of ontogeny; and (3) whether twins are compared directly after separation rather than at the end of ontogeny. These paradigms resemble those used in empirical research with humans in developmental psychology or epidemiology and with non-human animals in behavioral ecology (Frankenhuis and Walasek 2020). By varying the degree to which cues between separated twins differ we capture “dose-dependent experience studies” which, for example, study how matched individuals from the same litter respond to different dosages of the same treatment. We vary separation duration and measurement time to capture “cross-fostering studies”, in which a subset of individuals is removed from their natal environment and raised in a different environment for some time to disentangle the effects of rearing environment and subsequent differences in experience on phenotypic development. Differences between separated individuals and control individuals can be measured at the end of the separation duration, or at some later time after the separated individuals have been reintroduced to their original environment. By comparing these different paradigms, we are able to explore to what extent developmental trajectories of phenotypic plasticity uncovered in empirical studies may vary as a function of study paradigm. We depict the paradigms in Figure 1. We show the trajectories of phenotypic plasticity that result from these different paradigms in Supplementary Figures A4.2–A4.5.

Second, we explore the extent to which optimal decisions depend on phenotypic states versus posterior estimates. In our model, optimal decisions depend both on an organism’s phenotypic state and on its posterior estimate. Accordingly, organisms with identical posteriors might make different decisions because their previously constructed phenotypes differ. Other kinds of models, however, have assumed a one-to-one mapping between posteriors and phenotypes (e.g. Stamps and Krishnan 2014, 2017). To explore to what extent the inclusion of phenotypic states yields qualitatively different outcomes than a posterior-only model, we compare patterns of plasticity derived from both models when the reliability of cues varies across ontogeny. In line with our basic twin study paradigm, we compare the average proportional phenotypic distance and average difference in posterior estimates across 10 000 simulated pairs of twins at the end of ontogeny, following permanent separation.

## RESULTS

In the main text, we describe results for linear rewards and linear penalties. We present results from other combinations of reward and penalty functions in Supplementary Material 5 (Supplementary Figures A5.1–A5.27). We also provide additional analyses allowing comparison of results from this model with results of a previously published model of incremental development exploring fixed cue reliabilities (Panchanathan and Frankenhuis 2016), in Supplementary Material 1 (Supplementary Figures A1.2–A1.3) and Supplementary Material 5 (Supplementary Figures A5.28–A5.45).

### Sensitive periods may occur halfway through ontogeny

**Figure 2:**
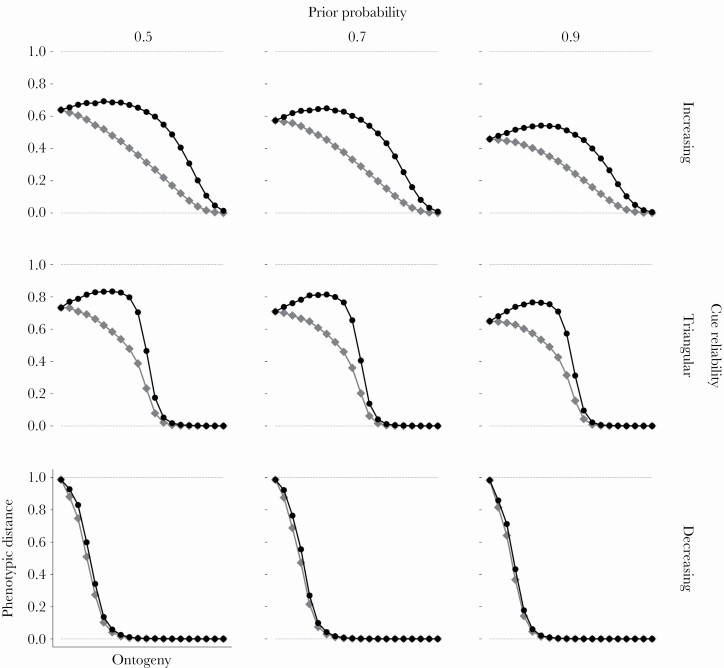
**Plasticity across ontogeny. The fitness rewards for correct specializations and fitness penalties for incorrect specializations are linear across all panels (see** **Supplementary Material 5, Figures A5.1–A5.9** **for other combinations of rewards and penalties). The prior probability of *E***_**1**_ **varies across columns and the cue reliability pattern varies across rows. Each panel represents *T* experimental “twin studies”, one for each t ∈{1, T}. Outcomes of each twin study are marked by a grey diamond and a black circle. For each study we simulate 10 000 pairs of identical twins who follow the optimal policy and track their development across ontogeny. The environmental state is fixed to *E***_**1**_ **. For each pair of twins, one individual (the “focal”) receives a set of environmental cues across ontogeny simulated from the prior probability and cue reliability pattern. Its clone receives the same cues until the moment of separation in time period *t* after which it begins to receive reciprocal, opposite cues, which lasts until the end of ontogeny. The vertical axis within each panel depicts the phenotypic distance between focal individuals and their clones. The horizontal axis depicts the time period in which pairs of twins were separated. The phenotypic distance at the end of ontogeny between a focal individual and its clone corresponds to the Euclidean distance between their phenotypes. Grey lines and diamonds depict “absolute” phenotypic distance, the average distance between the 10 000 focal individuals and their clones at the end of ontogeny (ranging from 0 to 20√2, scaled to a 0 to 1 range). Black lines and circles depict “proportional” distance, the average absolute distance divided by the maximum possible distance following separation.**

With absolute plasticity, sensitive periods are only favored early in ontogeny (Figure 2, grey lines and squares). With proportional plasticity, natural selection might favor sensitive periods in mid-ontogeny, but only if the cue reliability increases across ontogeny or first increases and then decreases, resulting in a triangular pattern (Figure 2, black lines and circles). Peaks are higher for the triangular cue reliability pattern, because the reliability of cues increases more rapidly during the first half of ontogeny. With decreasing cue reliabilities, sensitive periods evolve only early in ontogeny.

In most conditions, optimal policies track the cue reliabilities across time, meaning plasticity is highest when the cue reliability is highest. However, this is not always the case. When the cue reliability increases, plasticity peaks halfway through ontogeny, while cues are moderately reliable. By then, some organisms—those who have sampled consistent cue sets (see below)—have achieved a high level of confidence and their plasticity starts to decline.

Prior distributions only have a quantitative but not a qualitative impact on these patterns: the more uniform the prior distribution is, the lower the level of overall plasticity across ontogeny, as measured by the area under the curve. This small effect of prior is moderated by the cue reliability. Prior distributions have the strongest effect when cue reliabilities peak only at the end of ontogeny (increasing cue reliability). This makes sense. When information quality is low and one environmental state is much more likely than the other, organisms eschew plasticity and pick the more likely option.

Early in ontogeny prior distributions shape posterior estimates and thereby affect phenotypic development. However, as ontogeny proceeds and organisms sample more cues, the adjustment in posteriors and phenotypes in response to cues converges and becomes independent of the initial prior and cues take over in shaping both posteriors and phenotypes (Supplementary Material 1, Figure A1.4). Eventually, phenotypic plasticity declines regardless of the prior distribution and cue reliability pattern. Plasticity declines more steeply if organisms have access to reliable cues earlier in ontogeny, as is the case for the decreasing and triangular pattern. More reliable cues imply more consistency in cue sequences. Thus, the optimal policy instructs organisms to lose plasticity early in ontogeny and to invest in phenotypic specialization to reap fitness benefits (Frankenhuis and Panchanathan 2011a; Panchanathan and Frankenhuis 2016).

Previous models of stable environments also find that plasticity is higher early in ontogeny and then rapidly declines when cue reliability is high and constant across ontogeny (Frankenhuis and Panchanathan 2011a; Panchanathan and Frankenhuis 2016; Stamps and Krishnan 2017). Organisms use highly reliable cues at the onset of ontogeny to drastically reduce uncertainty about their environment, eliminating the need for continued plasticity. For the same reason, we find early-ontogeny sensitive periods with the decreasing cue reliability pattern. Combining our findings and those from previous models, we speculate that in environments that are stable across ontogeny, any pattern in which cues are highly reliable at the onset of ontogeny will lead to sensitive periods early in ontogeny. However, when the state of the environment fluctuates across ontogeny, we speculate that highly reliable cues early in ontogeny are often not sufficient to reduce uncertainty about future conditions. Under these conditions, natural selection may favor prolonged plasticity if the reliability of cues decreases across ontogeny, or even multiple sensitive periods if the cue reliability first decreases and then increases.

### Individual differences in sensitive periods

**Figure 3:**
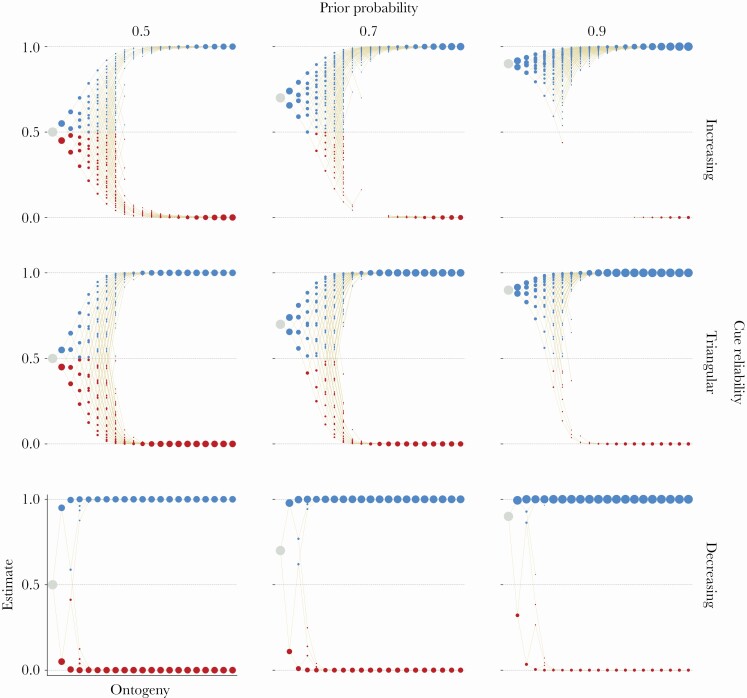
**Optimal developmental policies. The fitness rewards for correct specializations and fitness penalties for incorrect specializations are linear across all panels (see** **Supplementary Material 5, Figures A5.10–A5.18** **for other combinations of rewards and penalties). The prior probability of *E***_**1**_ **varies across columns and the cue reliability pattern varies across rows. Each panel depicts the optimal developmental policy for the corresponding parameter values as well as information about the probability of reaching each possible state. The horizontal axis shows developmental time and the vertical axis shows an organism’s estimate of being in *E***_**1**_ **. Each organism begins ontogeny with the same prior (large grey circle) and then, in each time period, samples a cue, updates its posterior, and makes a phenotypic decision. Beige lines represent possible changes in posteriors across development, tracking possible developmental trajectories. Colored circles represent phenotypic decisions: black indicates waiting, red specializing towards *P***_**0**_ **, and blue specializing towards *P***_**1**_ **. The area of a circle is proportional to the probability of reaching the corresponding state. These probabilities sum to one within a time period. We only show states that have a probability of more than 0.5% of being reached.**

Across the entire range of parameter values, natural selection favors early plasticity that declines across ontogeny and tends toward zero by the end (Figure 3). This gradual decline in plasticity, however, masks substantial individual variation in the onset, duration, and offset of sensitive periods. Organisms that receive consistent cue sets early in ontogeny become insensitive to cues earlier in ontogeny, whereas organisms that receive inconsistent cue sets prolong plasticity. Consistent cue sets are those in which a large fraction of cues indicate one environmental state over the other.

The consistency of cue sets is related to the cue reliability pattern. When the reliability of cues decreases, cue sets are relatively consistent early in ontogeny and inconsistent later in ontogeny. In this case, natural selection favors early sensitivity and a rapid decline in plasticity across ontogeny. When the reliability of cues first increases and then decreases (triangular pattern), cue set consistency at first increases and peaks at mid-ontogeny before turning and becoming increasingly inconsistent. Here, plasticity declines rapidly after mid-ontogeny. When the reliability of cues increases, early cue sets are inconsistent and become increasingly more consistent over time. Organisms in this case prolong plasticity well beyond mid-ontogeny.

### Repeatability depends on the environment

We track the proportion of rank-switches in a population of developing organisms across ontogeny to infer trait repeatability. This allows us to quantify and visualize how individual phenotypic differences develop and stabilize over time.

**Figure 4:**
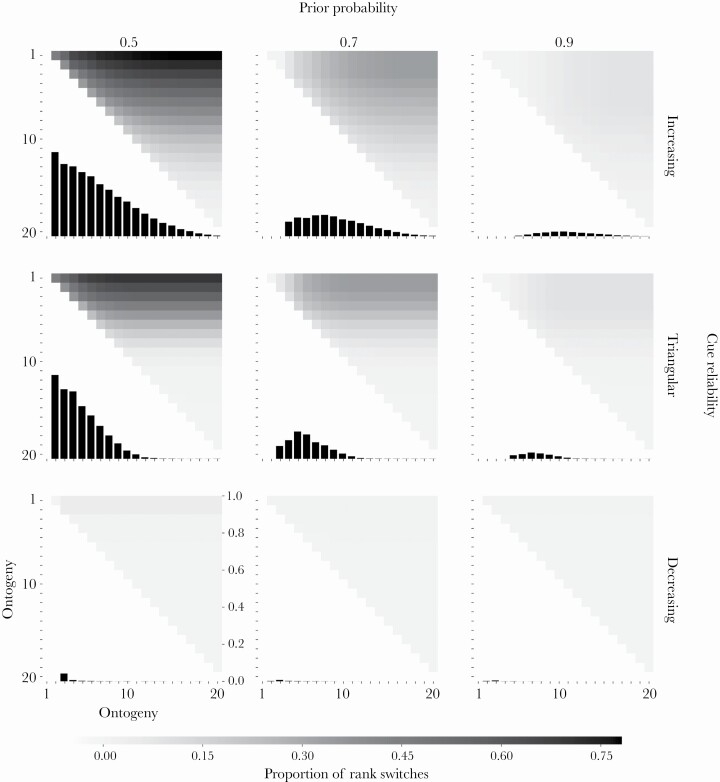
**Rank-order stability. The fitness rewards for correct specializations and fitness penalties for incorrect specializations are linear across all panels (see** **Supplementary Material 5, Figures A5.19–A5.27** **for other combinations of rewards and penalties). The prior probability of *E***_**1**_ **varies across columns and the cue reliability pattern varies across rows. Each panel depicts a simulation of 10 000 organisms following the optimal policy across ontogeny. The environmental state is fixed to *E***_**1**_ **. In each time period, organisms are ranked according to the number of specializations towards *P***_**1**_ **. Organisms with the same number of specializations share a rank. Each square panel depicts two sets of results, one in the upper right triangle and another in the lower left triangle. For the upper right triangle, the relevant axes are the horizontal and the left vertical, each depicting the full range of ontogenetic time periods. Each cell in this triangle indicates the proportion of rank-switches occurring from the time period on the horizontal axis to the time period on the (left) vertical axis in gray scale, with lighter cells indicating fewer rank-switches and darker cells more rank-switches. The lower left triangle within each panel zooms in on the diagonal of the upper right triangle, depicting the proportion of rank-switches between consecutive time periods in a bar chart. We highlight this scenario as it is the most relevant for empirical research on animal personality where repeatability is typically measured across consecutive years. For this portion of the panel, the horizontal axis depicts ontogenetic time periods and the right vertical axis depicts the proportion of rank-switches in that time period.**

Across prior distributions and cue reliability patterns, the proportion of rank-switches decreases as ontogeny proceeds indicating an increase in trait repeatability (bar charts in Figure 4). Over time organisms become more certain of their environmental state and consistently specialize towards it. When the prior distribution is uniform (0.5; left column, Figure 4), the decrease in rank-switches across ontogeny is accelerated when organisms have access to highly reliable cues early in ontogeny (decreasing cue reliability; bottom row, Figure 4). When cue reliability is low early in ontogeny (increasing or triangular cue reliability; top and middle rows, Figure 4), organisms “drift” early on, resulting in more rank-switches, and only settle on specialization trajectories later on, after sufficiently reducing uncertainty about the environmental state.

When the prior distribution is informative (0.7 or 0.9; middle and right columns, Figure 4), the proportion of rank-switches might increase during mid-ontogeny when the cue reliability starts out low and increases over time, as a consequence of increasing or triangular cue reliabilities; top and middle rows, Figure 4). Under these conditions, the majority of organisms within a population start specializing towards the same environmental state based on their priors, which keeps the proportion of rank-switches low. As the cue reliability begins to increase, organisms’ posteriors are more likely to shift, leading to more phenotypic “drifting”. This “drifting” increases the proportion of rank-switches and thus temporarily lowers trait repeatability.

### Results are robust to study paradigm

We conducted the simulated twin study (depicted in Figure 2) under different study paradigms, resembling those used in empirical studies of ontogenetic changes in phenotypic plasticity (Supplementary Material 4, Figure A4.1). To capture a wide variety of empirical paradigms, we vary three dimensions of our original twin study: (1) the degree to which cues sampled by the separated clone differ from those sampled by its identical twin; (2) whether separation is temporary and thus only lasts for a fixed number of time periods, or permanent until the end of ontogeny; and (3) whether twins are compared directly after separation or at the end of ontogeny.

We find that the degree to which cues between separated twins differ does not change qualitative changes in phenotypic plasticity, but merely influences the total magnitude of plasticity across ontogeny (Supplementary Material 4, Figure A4.2–A4.3). Not surprisingly, we observe that larger differences in sampled cues between separated twins result in greater magnitudes of phenotypic plasticity across ontogeny. When the separation of twins is temporary, plasticity measured at the end of ontogeny reflects the long-term effects of this separation. This measure illustrates to what extent the time periods in which twins have reunited buffer against further phenotypic divergence or even initiate phenotypic convergence of twins. When plasticity is measured at the end of ontogeny and cue reliabilities increase, plasticity nonetheless tends to increase towards the end of ontogeny (Supplementary Material 4, Figure A4.4, first row). This indicates that highly reliable cues have a major long-term effect on phenotypic development, even in the later stages of ontogeny. This enduring effect cannot be compensated for by short time windows in which twins have reunited. Plasticity measured directly after temporary separation quantifies the short-term, immediate effects of separation (Supplementary Material 4, Figures A4.5). Immediate effects of separation are largest if separation occurs when twins are uncertain about environmental conditions due to a uniform prior distribution and/or when cues are highly reliable during the window of separation.

### Mid-ontogeny sensitive periods might depend on both phenotypes and posteriors

**Figure 5:**
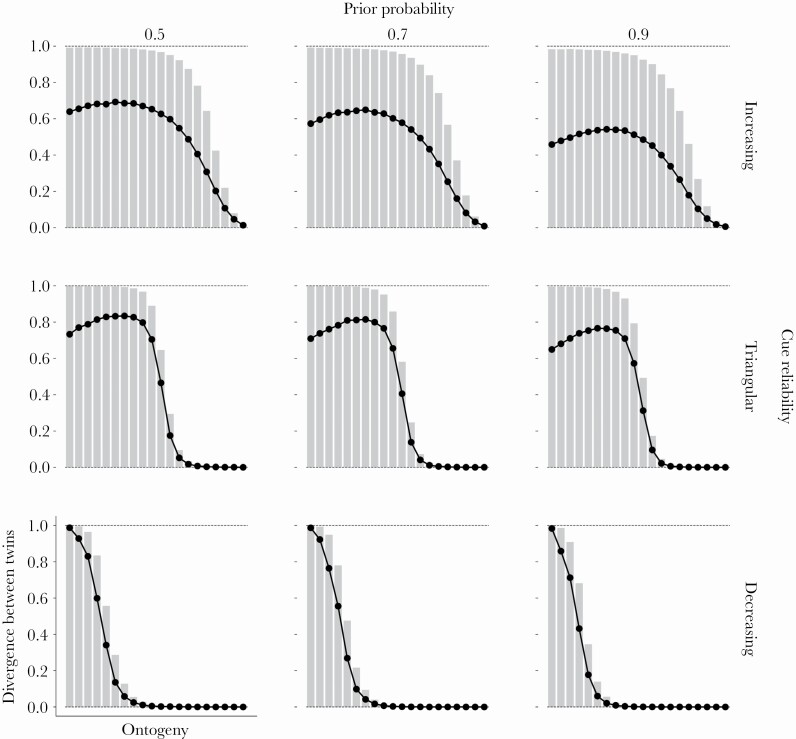
**Plasticity in phenotype and posterior estimate. The fitness rewards for correct specializations and fitness penalties for incorrect specializations are linear across all panels. The prior probability of *E***_**1**_ **varies across columns and the cue reliability pattern varies across rows. As in Figure 2** **, each panel represents *T* experimental “twin studies”, one for each t ∈{1, T}. Black circles correspond to the phenotype-and-posterior and gray bars to the posterior-only model. For each study we simulate 10 000 pairs of identical twins who follow the optimal policy and track their development across ontogeny. The environmental state is fixed to *E***_**1**_ **. For each pair of twins, one individual (the “focal”) receives a set of environmental cues across ontogeny simulated from the prior probability and cue reliability pattern. Its clone receives the same cues until the moment of separation in time period *t* after which it begins to receive reciprocal, opposite cues, which lasts until the end of ontogeny. The vertical axis within each panel depicts the difference between focal individuals and their clones in the phenotype-and-posterior model and the posterior-only model. The horizontal axis depicts the time period in which pairs of twins were separated. Black lines and circles depict the average Euclidean distance between the 10 000 focal individuals and their clones at the end of ontogeny (ranging from 0 to 20√2, scaled to a 0 to 1 range), divided by the maximum possible distance following separation. Gray bars correspond the average absolute distance in posteriors between those same simulated organisms at the end of ontogeny.**

As part of our sensitivity analysis, we explore whether resulting patterns of sensitive periods depend on our assumption of modeling phenotypic states alongside information states. Specifically, we compare patterns of plasticity in a phenotype-and-posterior model (black lines and circles, Figure 4), in which developmental decisions are shaped by phenotypic states that are coupled with posterior estimates, and a posterior-only model (gray bars, Figure 5), which assumes a one-to-one mapping between phenotypes and posteriors. Unfortunately, we are not able to meaningfully interpret the differences in magnitude of plasticity between both models, because differences in posterior estimates and differences in phenotypes are measured in different units. The former is computed as the difference in posterior probabilities while the latter is computed as the normalized Euclidean distance between phenotypes. Thus, we will only discuss qualitative differences in patterns of plasticity across models.

Qualitative patterns look largely similar across models. Across all parameter combinations, phenotypic plasticity tends to decline with age. However, we also observe differences: phenotypic distances across separated twins might increase when separation occurs later during ontogeny, while differences in posterior estimates between those same twins decrease or remain unchanged. To illustrate this difference, we plot the gradients of both plasticity curves in Supplementary Material 1 (Supplementary Figure A1.5). This result implies that natural selection favors mid-ontogeny increases in plasticity in the phenotype-and-posterior model but not in the posterior-only model. Both phenotypic state and information available thus act as selection pressures in our model when shaping mid-ontogeny sensitive periods. Thus, only modeling the information state of an organism is not sufficient to explain mid-ontogeny sensitive periods.

## DISCUSSION

We have modeled the evolution and development of sensitive periods when organisms construct their phenotypes incrementally and the reliability of cues varies across ontogeny. We used stochastic dynamic programming to compute optimal developmental policies across a range of evolutionary ecologies, varying the prior distribution of environments, the cue reliability pattern, and the mapping of phenotype onto fitness. From these optimal policies, we derived changes in phenotypic plasticity across ontogeny. We discuss five insights from our model and limitations and future directions.

### Mid-ontogeny sensitive periods may evolve when the reliability of cues increases

We find that sensitive periods evolve in mid-ontogeny when the reliability of cues is low at the onset and increases over, at least some portion of, ontogeny. Unlike previous models (Fischer et al. 2014; Stamps and Krishnan 2017) we find mid-ontogeny sensitive periods when prior and acquired information are consistent with each other, thus identifying increases in cue reliability as the cause of increases in plasticity in our model. Moreover, whereas the previous models find a relatively small plasticity bump at the beginning of ontogeny, our model produces bumps that extend across a substantial portion of ontogeny.

Fuhrmann et al. (2015) reviewed evidence for adolescence being a sensitive period of brain development in humans and distinguished three models of plasticity: a discrete and punctuated period of heightened plasticity in adolescence, a continuous and constant sensitive period across childhood and adolescence, or a continuous and gradual decline of plasticity across childhood and adolescence. Our modeling results suggest that natural selection can favor each of these three models, depending on the evolutionary ecology. For example, if cues were, on average, unreliable early and late in ontogeny, with a peak in mid-ontogeny, natural selection might favor a discrete period of heightened plasticity during mid-ontogeny. When cues are, at first, unreliable and gradually increase in reliability across ontogeny, natural selection might favor a continuous sensitive period across childhood. This pattern is especially favored when the distribution of environmental states is uniform and thus making it harder for developing organisms to predict their environment before having sampled any cues. Lastly, if cues are at first highly reliable and decline in reliability across ontogeny, natural selection might favor an initially high period of sensitivity with a continuous decline in plasticity across childhood.

### There are individual differences in the timing of sensitive periods

Using a similar modeling framework to this paper’s, Frankenhuis and Panchanathan (2011a, 2011b) and Panchanathan and Frankenhuis (2016) showed that individuals who sample more consistent cue sets might shed plasticity earlier in ontogeny. Here, we add that the opportunity to gather reliable information later in ontogeny might prolong sensitive periods beyond early ontogeny, even resulting in mid-ontogeny sensitive periods. However, organisms of the same population show inter-individual differences in the level of elevation of plasticity during these mid-ontogeny sensitive periods. Because cues are noisy, organisms of the same population will vary in the extent to which they are certain of the state of their environment due to sampling different sequences of cues, with inconsistent sequences of cues resulting in more uncertainty. The more uncertain organisms are when the opportunity to gather reliable information arises, the higher their peaks in mid-ontogeny sensitive periods. This finding suggests that empirical studies are most likely to observe mid-ontogeny sensitive periods if organisms start out uncertain (e.g. have experimentally evolved a uniform prior) and receive highly reliable cues midway ontogeny.

### Individual differences tend to stabilize across ontogeny

Kok et al. (2019) suggest that increased exposure to reliable cues across ontogeny might reduce organisms’ uncertainty about their environment, leading to fewer adjustments of phenotypic traits, such as exploration behavior. This process, they argue, might cause age-related increases in trait repeatability. When organisms in our model are initially uncertain about their environment due to a uniform prior distribution and the reliability of cues increases early in ontogeny, we observe such a pattern of increased trait repeatability. Trait repeatability develops earlier in ontogeny the earlier organisms have access to reliable cues.

Research on animal personality includes a focus on the repeatability of phenotypic traits across an organism’s lifetime (Sih et al. 2004). The typical pattern in this literature is that the repeatability of phenotypic traits increases across ontogeny in a variety of species, including humans (Sih et al. 2004; Fraley and Roberts 2005; Réale and Dingemanse 2012). However, not all studies find this pattern. Wuerz and Krüger (2015), for example, observed large variation in repeatability across different traits and life stages in Zebra finches. Some traits showed no repeatability, while others were only repeatable in some portions of ontogeny. In some cases repeatability even decreased across life stages. Although in our model trait repeatability usually increases, we do find, for instance, that with informative prior distributions (0.7 or 0.9) and increasing or triangular (first increasing, then decreasing) cue reliabilities, trait repeatability might decrease in mid-ontogeny rather than monotonically increase across ontogeny. Our model thus suggests hypotheses about the selection pressures that can result in the more common pattern of increasing trait repeatability and in the less common pattern of decreasing trait repeatability.

### Results are robust to study paradigm

When applying different paradigms to quantify phenotypic plasticity, we only observed changes in the overall magnitude of plasticity but no qualitative changes in the patterns of sensitive periods across ontogeny. We found greater magnitudes of plasticity across ontogeny when simulated twins were exposed to drastically different cues during their separation. Although we do not imply that our model findings are readily applicable to empirical studies of phenotypic plasticity, we do think that they raise two empirical questions that are surely worth exploring: first, whether patterns of plasticity observed in empirical studies using different experimental manipulations are comparable; and second, whether plasticity is larger and easier to detect in empirical studies using more extreme manipulations of individuals’ experiences. As a first step, studies might compare trajectories in plasticity derived from different study paradigms in the same species and for the same trait of interest.

### Sensitive periods depend on information and phenotypic state

Mid-ontogeny sensitive periods only emerge when the state of organisms includes both posteriors as well as phenotypes, not when state only includes posteriors, if plasticity is measured at the end of ontogeny. However, it is unknown to what extent this finding depends on our specific model assumptions. Thus, an open question is whether the inclusion of phenotypic states is generally, across models of sensitive periods, required for the evolution of mid-ontogeny sensitive periods.

Future work could systematically compare outcomes from posterior-only and phenotype-and-posterior models across different study paradigms (e.g. measuring plasticity at different time points) and model assumptions (e.g. fixed or varying cue reliabilities, stable or fluctuating environments) to study whether the inclusion of phenotypic states is necessary for sensitive periods to be favored in later developmental stages. We have made a small step in this direction by comparing both kinds of models, when differences in posteriors and phenotypes between simulated twins are measured right after their separation, rather than at the end of ontogeny (Supplementary Material 1, Figure A1.4). This measure quantifies the immediate phenotypic effects of the experimental manipulation. In that scenario, we find mid-ontogeny sensitive periods for both a posterior-only and a phenotype-and-posterior model.

### Limitations and future directions

We first discuss two specific limitations of our model and then two broader limitations of this class of models. We also suggest future directions that can address some of these limitations.

First, in our model, the environment remains stable within an individual’s lifetime. Whether this assumption is plausible for a given species depends on its generation time relative to the rate of environmental change (Botero et al. 2015). For long-lived organisms, it is less likely that the environment remains stable throughout their lifetime (Nettle et al. 2013). Also, in a seasonally changing environment, natural selection might increase plasticity at those times when learning and development, or changes in behavior, might enhance fitness. For instance, seasonally breeding adult songbirds exhibit seasonal plasticity in song behavior and the associated brain regions (Tramontin and Brenowitz 2000). Our model assumes a stable environment throughout the organism’s lifespan and so is not designed to capture such phenomena.

Second, we constrained ontogeny to a fixed time horizon. In nature, however, different individuals of the same species might mature at different times as a result of phenotypic plasticity or other processes. In our model, the duration of ontogeny is fixed and fitness is accrued at the end of ontogeny. Plasticity might terminate towards the end of ontogeny, because the remaining time is too short to revise estimates and switch developmental trajectories. Future modeling might explore the evolution of sensitive periods when the time horizon is uncertain (e.g. in each time period, there is some fixed or increasing probability of extrinsic mortality; Mangel and Clark 1988).

Third, models like ours are agnostic about mechanism. They exclusively consider the impact of experience on phenotype (Frankenhuis and Walasek 2020). As a consequence, such models cannot be used to make predictions about the physiological processes that guide changes in plasticity across ontogeny. Nonetheless, models can help focus research efforts on hypotheses about mechanisms that produce the patterns generated by qualitative models that themselves do not incorporate mechanism.

Fourth, in models like ours, the environmental state is typically the only unknown quantity and organisms know how to optimally respond to it. Organisms learn about the state of their environment based on cues and know the optimal developmental decision given their current state. However, real organisms might need to learn about other environmental quantities, such as the reliability of cues, or about the optimal adaptive response to different states. A large body of research on reversal learning shows that organisms are capable to infer the reliability of cues in nature (Izquierdo et al. 2017). Trout, for example, learn to recognize the sight or smell of potential predators (Behrens et al. 2007; Horn et al. 2019). When studying phenotypic plasticity in cases where the optimal response is known to the organism, we study so-called “switch-like” plasticity (Snell-Rood 2012; Frankenhuis et al. 2019). This captures a variety of traits and species. The development of defensive armor in Daphnia in response to chemical predator cues is a well-known example (Agrawal et al. 1999). However, in other cases, organisms learn to respond adaptively via trial-and-error. For example, the circulatory, nervous, and immune systems are able to learn some adaptive responses from feedback (Snell-Rood 2012). Natural selection has, in these cases, equipped organisms with the ability to learn the adaptive response based on trial-and-error (i.e. developmental selection) (Snell-Rood 2012; Frankenhuis et al. 2019).

Future models of the evolution of sensitive periods might vary the environmental state within the lifetime of an organism, explore the consequences of a probabilistic time horizon on the cessation of ontogeny, or integrate known proximate mechanisms (McNamara and Houston 2009; Kacelnik 2012; Trimmer et al. 2012; Frankenhuis et al. 2019; Taborsky et al. 2020). Incorporating additional learning mechanisms, such as trial-and-error, represents another interesting future direction that might make models like ours more relevant to empirical studies of phenotypic plasticity. Specifically, organisms might learn about the reliability of cues or the adaptive response to different states. Reinforcement learning models are promising tools to approach both types of learning problems (Frankenhuis et al. 2019).

## CONCLUSION

By showing that sensitive periods can be favored by natural selection beyond early life if the reliability of cues increases across ontogeny, our model contributes to a growing set of models exploring the selection pressures shaping the evolution of sensitive periods in development. Together, this family of models has the potential to develop into an integrative theoretical framework of the evolution and development of sensitive periods, which is firmly anchored in a classic and well-developed body of theory exploring the evolution of phenotypic plasticity more generally.

## Supplementary Material

arab113_suppl_Supplementary_Material

## Data Availability

Analyses reported in this article can be reproduced using the code provided by Walasek (2021).
