# Supplementary material for: An evolutionary model of sensitive periods when the reliability of cues varies across ontogeny
Source: Behav Ecol. 2021 Oct 25;33(1):101–14. doi: 10.1093/beheco/arab113 (PMC8857937; doi:10.1093/beheco/arab113)
Supplement: arab113_suppl_Supplementary_Material [file arab113_suppl_supplementary_material.pdf]

Supplementary Material

An evolutionary model of sensitive periods when the reliability of cues varies across ontogeny

SM 1	Additional plots for 20 time steps	Maximal cue reliability 0.75	3
		Mature phenotypes	5
		Fitness of mature phenotypes	6
		Phenotypic plasticity and plasticity in belief	7
		Gradients of plasticity curves	8
SM 2	Dynamic programming	Environmental variables and state of an organism	9
		Bayesian inference	10
		Fitness functions	11-12
		Optimal decisions	13
SM 3	Bayesian inference visualized		14-17
SM 4	Measuring changes in plasticity across ontogeny		18-22
SM 5	Main plots for all penalty and reward functions (10 time steps)	Plasticity across ontogeny	24-32
		Optimal developmental policies	33-41
		Rank-order stability	42-50
		Mature phenotypes	51-59
		Fitness of mature phenotypes	60-68

SM 1 – Additional plots for 20 time steps

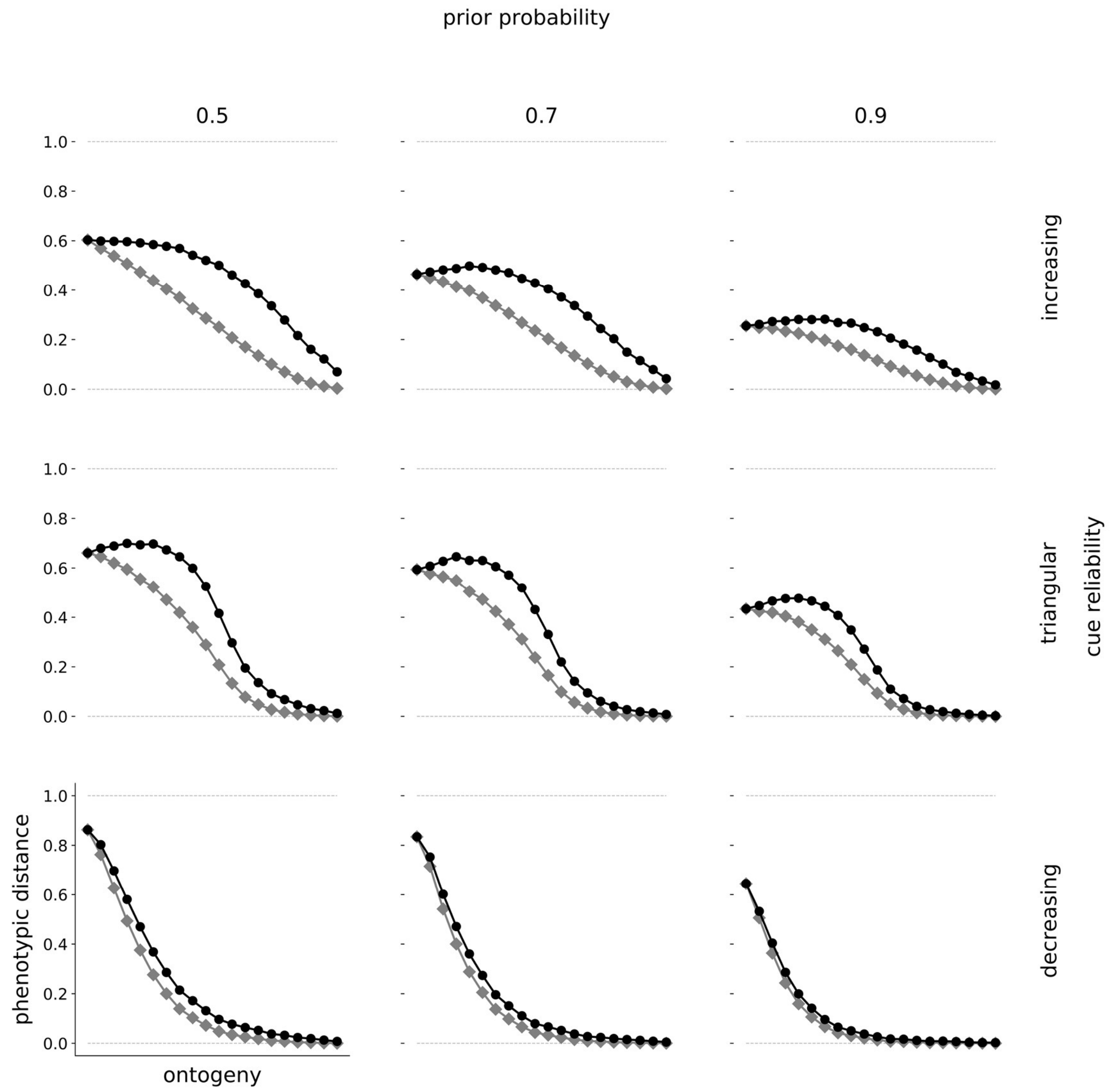

Figure A1.1. Plasticity across ontogeny – maximal cue reliability of 0.75. The fitness rewards for correct specializations and fitness penalties for incorrect specializations are linear across all panels. The prior probability of E_1 varies across columns and the cue reliability pattern varies across rows. Each panel represents T experimental 'twin studies', one for each $t \in \{1, T\}$. Outcomes of each twin study are marked by a grey diamond and a black circle. For each study we simulate 10,000 pairs of identical twins who follow the optimal policy and track their development across ontogeny. The environmental state is fixed to E_1. For each pair of twins, one individual (the 'focal') receives a set of environmental cues across ontogeny simulated from the prior probability and cue reliability pattern. Its clone receives the same cues until the moment of separation in time period t after which it begins to receive reciprocal, opposite cues, which lasts until the end of ontogeny. The vertical axis within each panel depicts the phenotypic distance between focal individuals and their clones. The horizontal axis depicts the time period in which pairs of twins were separated. The phenotypic distance at the end of ontogeny between a focal individual and its clone corresponds to the Euclidean distance between their phenotypes. Grey lines and diamonds depict 'absolute' phenotypic distance, the average distance between the 10,000 focal individuals and their clones at the end of ontogeny (ranging from 0 to $20\sqrt{2}$, scaled to a 0 to 1 range). Black lines and circles depict 'proportional' distance, the average absolute distance divided by the maximum possible distance following separation.

Here, we additionally show distributions of mature phenotypes and compare the fitness of the optimal policy with the fitness of two non-plastic strategies, a generalist and a specialist, to get a sense of whether and by how much the optimal policy outperforms simpler strategies. We do not discuss results from these analyses in the main text, as the results are qualitatively similar to those of a model with fixed cue reliabilities (Panchanathan & Frankenhuis, 2016[1]). The main text focuses on those results that are qualitatively different when cue reliabilities are variable rather than fixed across ontogeny.

[1] Panchanathan K, Frankenhuis WE. 2016 The evolution of sensitive periods in a model of incremental development. *Proc. Royal Soc. B* **283**, 20152439. (doi:10.1098/rspb.2015.2439)

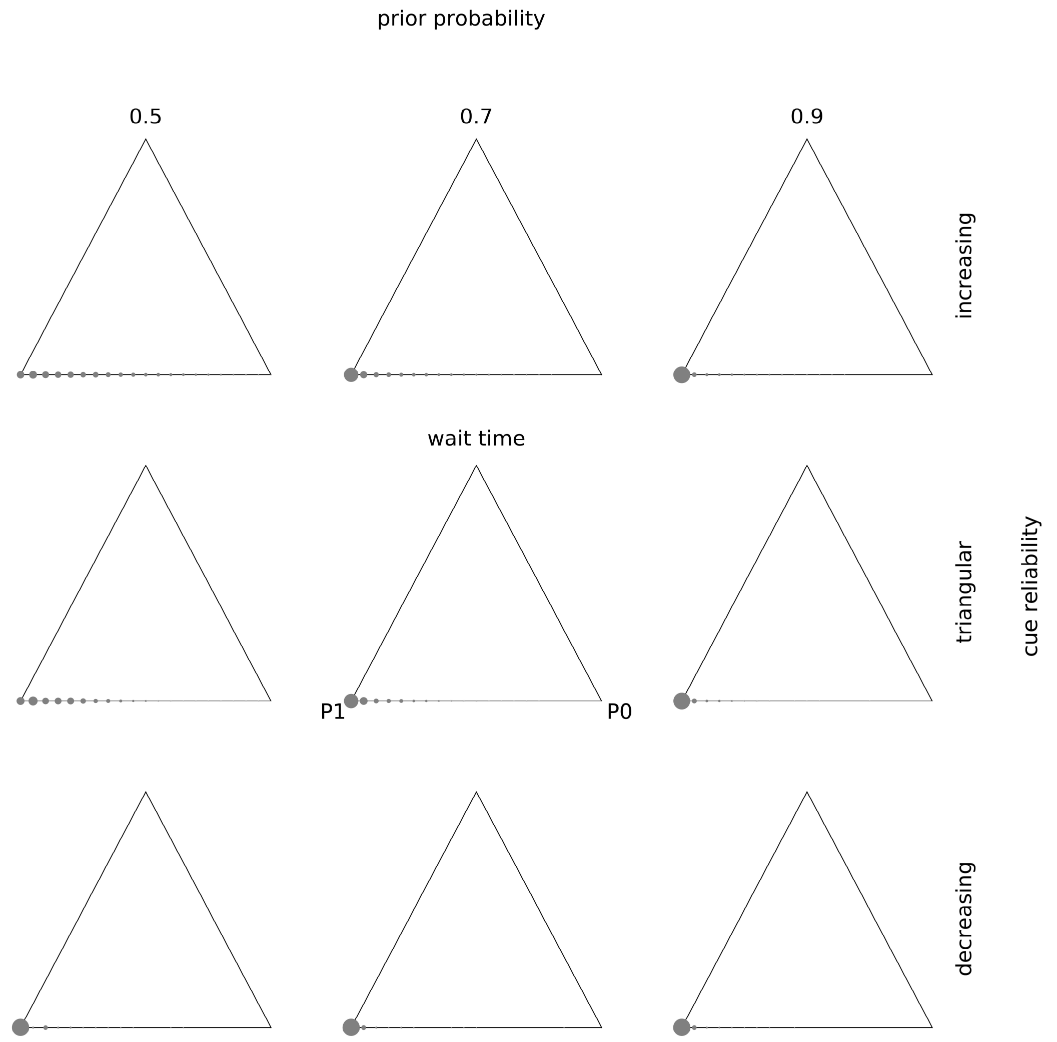

Figure A1.2. Distributions of mature phenotypes. The fitness rewards for correct specializations and fitness penalties for incorrect specializations are linear across all panels. The prior probability of E_1 varies across columns and the cue reliability pattern varies across rows. Each panel represents a simulation study. For each study we simulate 10,000 organisms who follow the optimal policy and track their development across ontogeny. The environmental state is fixed to E_1. Each triangle plots the distribution of phenotypes at the end of ontogeny. The number of time periods waited, time periods specialized towards P_1 and time periods specialized towards P_0 make up a phenotype. The position of a circle indicates the composition of mature phenotypes. The left and right vertices represent organisms that only specialized towards P_1 and P_0, respectively. The top vertex represents organisms that only waited. Circles on the outer boundary indicate a mixture of two phenotypic decisions, while circles within the triangle indicate a mixture of all three decisions. The area of a circle is proportional to the fraction of simulated organisms that developed the same phenotype.

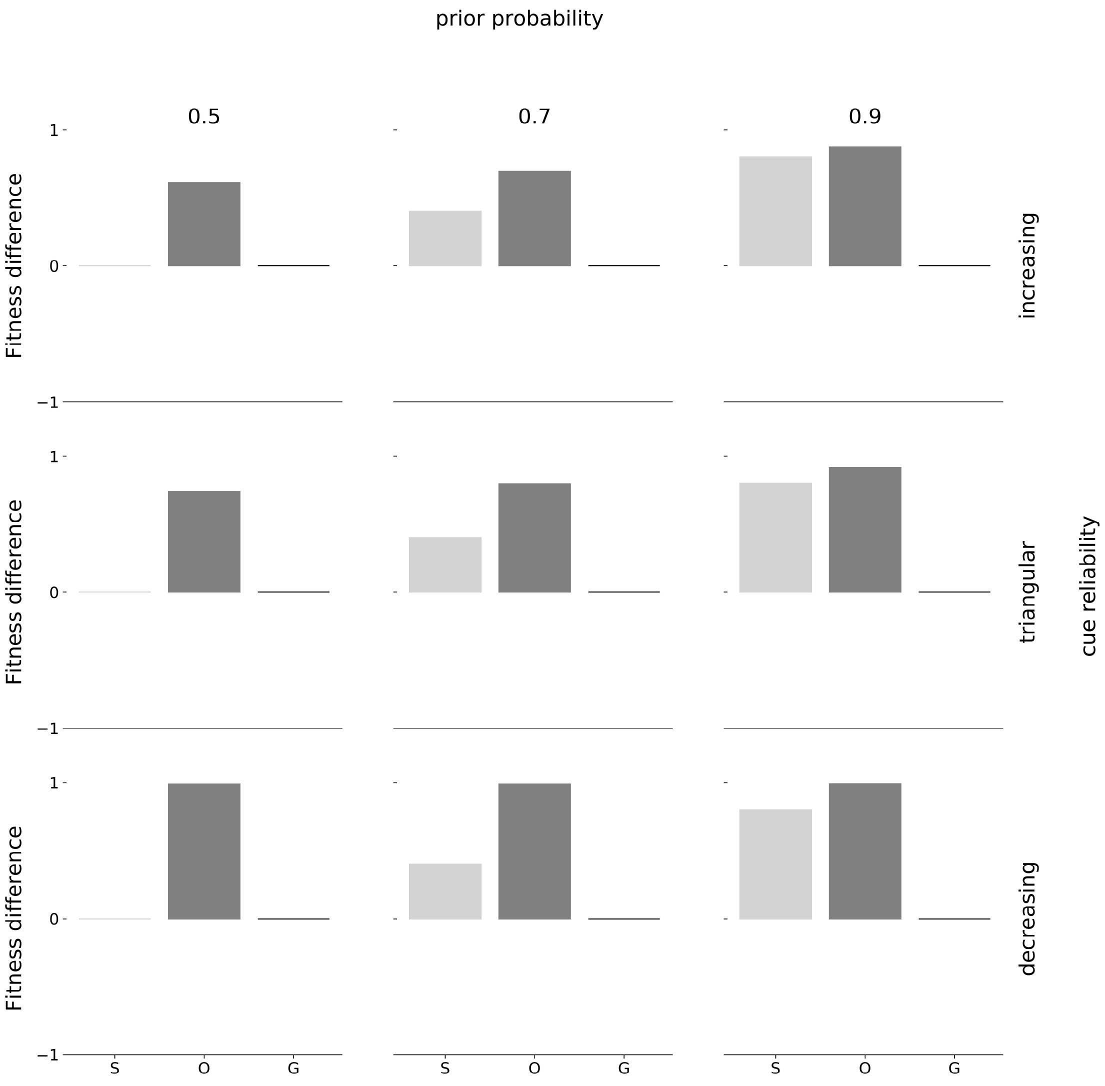

Figure A1.3. Fitness of mature phenotypes. The fitness rewards for correct specializations and fitness penalties for incorrect specializations are linear across all panels. The prior probability of E_1 varies across columns and the cue reliability pattern varies across rows. Each panel represents a simulation study. For each study we simulate 10,000 organisms who follow the optimal policy and track their development across ontogeny. For half of the population the environmental state is fixed to E_0 and for the other half to E_1 (5,000 organisms per environment).We then compare the average fitness across organisms following the optimal policy ('O'; center, dark-grey bar) to two non-plastic strategies: generalists ('G'; right, black bar) and specialists ('S'; left, light-grey bar). Generalists always specialize halfway towards each phenotypic target, while specialists specialize towards the phenotypic target that is more likely according to the prior. If the prior is 0.5 organisms choose a target at random. Bars indicate the expected fitness difference from baseline (marked as 0) of the three strategies, normalized to range between -1 and 1 with 1 indicating a perfect match to the environment. Fitness differences can be negative when mismatch penalties exceed rewards for correct matches.

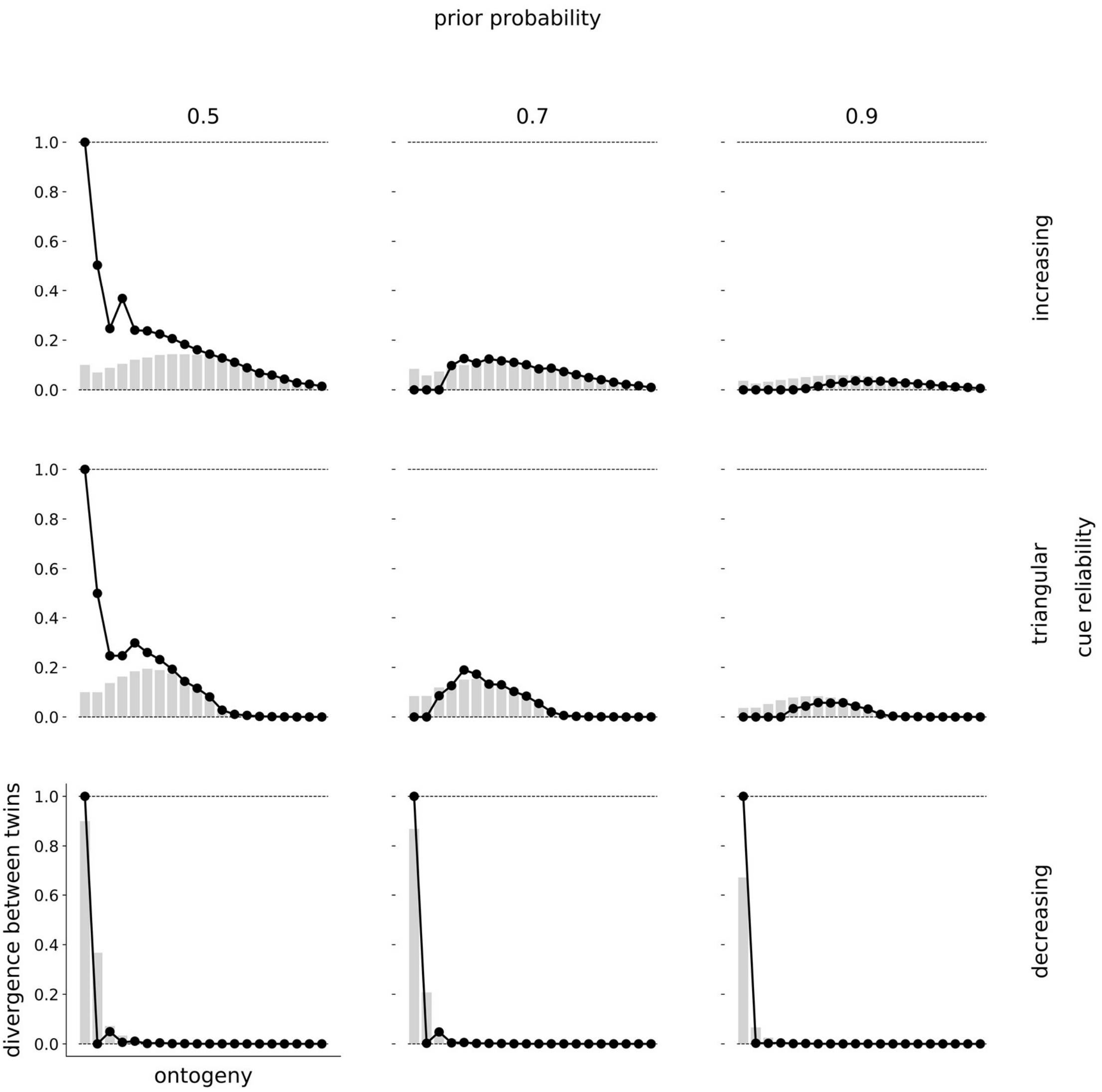

Figure A1.4. Plasticity in phenotype and posterior estimate. The fitness rewards for correct specializations and fitness penalties for incorrect specializations are linear across all panels. The prior probability of E_1 varies across columns and the cue reliability pattern varies across rows. Each panel represents T experimental 'twin studies', one for each $t \in \{1, T\}$. Black circles correspond to the phenotype-and-posterior and gray bars to the posterior-only model. For each study we simulate 10,000 pairs of identical twins who follow the optimal policy and track their development across ontogeny. The environmental state is fixed to E_1. For each pair of twins, one individual (the 'focal') receives a set of environmental cues across ontogeny simulated from the prior probability and cue reliability pattern. Its clone receives the same cues until the moment of separation in time period t after which it receives one reciprocal, opposite cue, and then continues normal development with its twin until the end of ontogeny. The vertical axis within each panel depicts the difference between focal individuals and their clones in the phenotype-and-posterior model and the posterior-only model. The horizontal axis depicts the time period in which pairs of twins were separated. Black lines and circles depict the average Euclidean distance between the 10,000 focal individuals and their clones after their separation (scaled to a 0 to 1 range), divided by the maximum possible distance attainable within one time period. Gray bars correspond the average absolute distance in posteriors between those same simulated organisms after their separation.

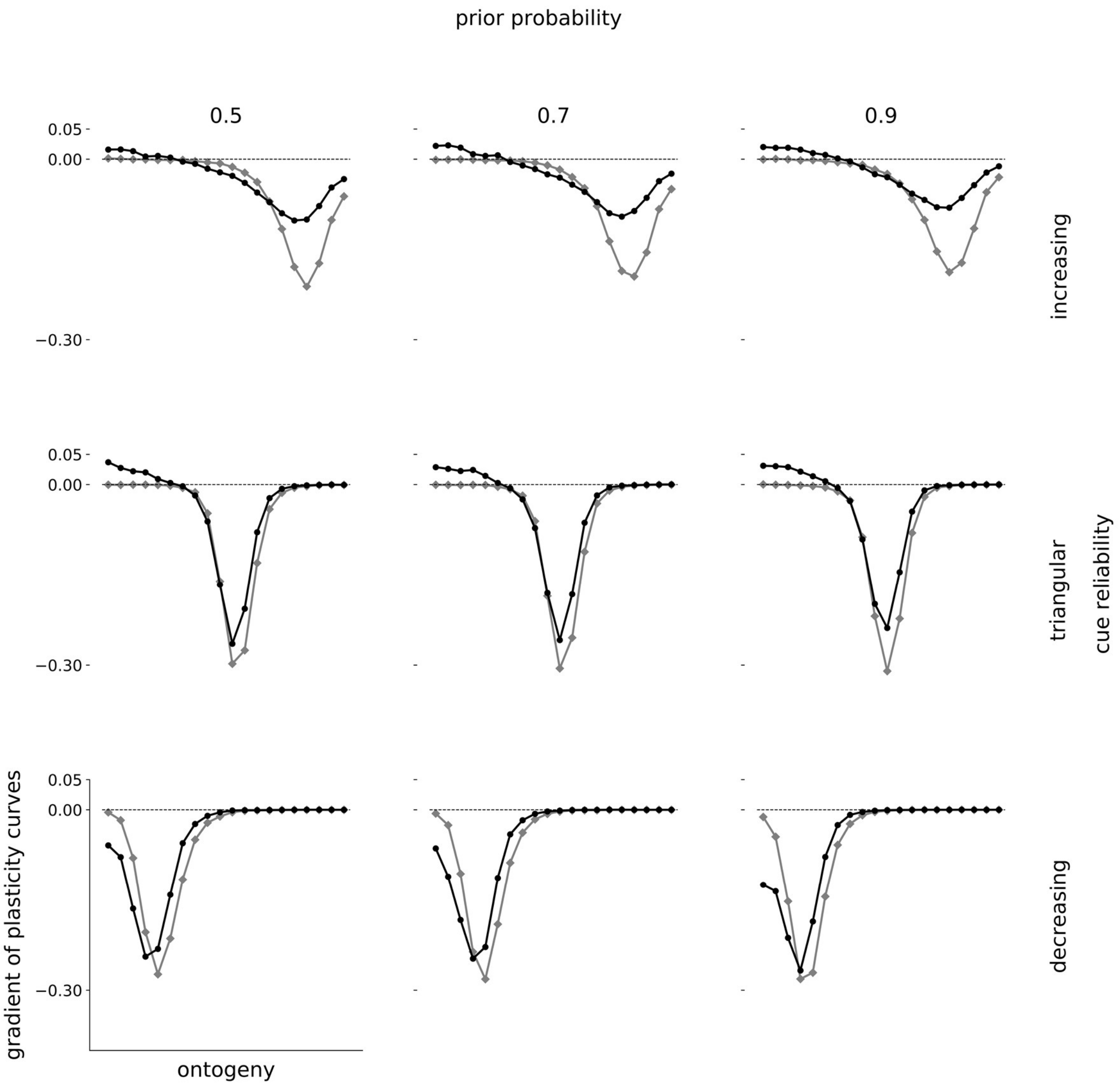

Figure A1.5. Gradients of phenotypic plasticity and plasticity in posterior estimates. The fitness rewards for correct specializations and fitness penalties for incorrect specializations are linear across all panels. The prior probability of E_1 varies across columns and the cue reliability pattern varies across rows. Each panel represents T experimental 'twin studies', one for each $t \in \{1, T\}$. Black lines and circles correspond to the phenotype-and-posterior and gray lines and diamonds to the posterior-only model. For each study we simulate 10,000 pairs of identical twins who follow the optimal policy and track their development across ontogeny. The environmental state is fixed to E_1. For each pair of twins, one individual (the 'focal') receives a set of environmental cues across ontogeny simulated from the prior probability and cue reliability pattern. Its clone receives the same cues until the moment of separation in time period t after which it begins to receive reciprocal, opposite cues, which lasts until the end of ontogeny. Within each panel we compute the difference between focal individuals and their clones in the phenotype-and-posterior model and the posterior-only model. In the phenotype-and-posterior model this difference is the average Euclidean distance between the 10,000 focal individuals and their clones at the end of ontogeny (ranging from 0 to $20\sqrt{2}$, scaled to a 0 to 1 range), divided by the maximum possible distance following separation. In the posterior-only model this difference is the average absolute distance in posteriors between those same simulated organisms at the end of ontogeny. The horizontal axis depicts the time period in which pairs of twins were separated. The vertical axis within each panel depicts the gradients of the resulting plasticity trajectories from the phenotype-and-posterior and the posterior-only model.

SM 2 – Dynamic programming equations

a) Environmental variables and state of an organism

Environmental variable	Explanation
E_0	Environment 0
E_1	Environment 1
P_0	Optimal phenotype for E_0
P_1	Optimal phenotype for E_1
C_0	Cue indicating E_0
C_1	Cue indicating E_1
T	End of ontogeny, i.e., 20 for the results in the main text

The state of an organism is characterized by a 5-tuple (D_t, y_0, y_1, y_w, t). In each time step (from 1 until T) organisms first sample a cue and then make a phenotypic decision. D_t denotes the sequence of cues that an organism has sampled by time period t.

Variable	Explanation
D_t	$D_t = \{x_{1,} \quad x_{2,} \quad \cdots \quad x_{t,}\}$, where x_1, x_2 up until x_t denote the kind of cue (C_0 or C_1) received in each time period
y_0	Number of specialization steps towards P_0
y_1	Number of specialization steps towards P_1
y_w	Number of time steps spent waiting
t	Current time period in ontogeny

b) Bayesian inference

Organisms use Bayesian inference to update their initial prior estimate of the environmental state based on the sampled cues.

Parameters for Bayesian inference	Explanation
$P(E_0)$	Prior probability of E_0
$P(E_1)$	Prior probability of E_1
$P(C_{0,t}\|E_0)$	Cue reliability; conditional probability of receiving C_0 in E_0 at t
$P(C_{1,t}\|E_1)$	Cue reliability; conditional probability of receiving C_1 in E_1 at t
$P(E_0\|D_t)$	Posterior probability of E_0 after having sampled D_t
$P(E_1\|D_t)$	Posterior probability of E_1 after having sampled D_t

According to the laws of probability it holds that:

$$P(E_0) + P(E_1) = 1$$

$$P(E_0|D_t) + P(E_1|D_t) = 1$$

$$P\left(C_{0,t}|E_0\right) + P\left(C_{1,t}|E_0\right) = 1$$

$$P\left(C_{1,t}|E_1\right) + P\left(C_{0,t}|E_1\right) = 1$$

Further, we assume that $P\left(C_{0,t}|E_0\right) = P\left(C_{1,t}|E_1\right)$.

We assume that organisms are Bayesian learners, using the fixed distribution of patches as the prior estimate of the environmental state and the time-dependent cue reliabilities to update these estimates. To see how this works, suppose an organism has sampled a specific sequence of cues $D_{t=3} = \{x_1 = C_0, \quad x_2 = C_1, \quad \dots \quad x_3 = C_0\}$.

According to Bayes' theorem, its posterior estimate after the first cue is:

$$P(E_0|C_0) = \frac{P(C_0|E_0) \bullet P(E_0)}{P(C_0|E_0) \bullet P(E_0) + P(C_0|E_1) \bullet P(E_1)}$$

$$P(E_1|C_0) = 1 - P(E_0|C_0)$$

To compute the posteriors $P(E_0|D_t)$ and $P(E_1|D_t)$ after the whole sequence of cues, we have to reapply Bayes' theorem for each cue using the previous posterior as the new prior.

c) Fitness functions

We denote the mature phenotype at the end of ontogeny by $Y_{mat} = (y_0, y_1, T)$.

Functions and constants	Explanation
$\phi(Y_{mat})$	Expected, additive fitness reward at the end of ontogeny
$\psi(Y_{mat})$	Expected, additive fitness penalty at the end of ontogeny
$\pi(Y_{mat})$	Expected fitness at the end of ontogeny
π_0	Baseline fitness
$f(y)$	Mapping between phenotypic increments and fitness rewards (or penalties)

Fitness consequences of phenotypic decisions are not accrued throughout ontogeny but only at the end of ontogeny. The fitness difference from baseline at the end of ontogeny corresponds to the total rewards for correct specializations minus penalties from incorrect specializations, where each correct increment results in a marginal gain and each incorrect increment results in a marginal penalty. We studied three mappings between correct (or incorrect) phenotypic development and fitness rewards (or penalties).

Suppose a mature organism is in the following state at the end of ontogeny (D_T, y_0, y_1, y_w, T) having sampled the cue sequence D_T and developed the mature phenotype $Y_{mat} = \{y_0, y_1, T\}$. Developing organisms aim to maximize expected fitness at the end of ontogeny. Expected fitness $\pi(Y_{mat})$ corresponds to the sum of expected rewards and penalties, in addition to the baseline fitness:

$$\pi(Y_{mat}) = \pi_0 + \phi(Y_{mat}) + \psi(Y_{mat}).$$

Suppose that an organism has sampled a specific sequence of cues, D_t, throughout ontogeny. Its posterior estimates $P(E_0|D_{t=T})$ and $P(E_1|D_{t=T})$ reflect the probabilities of being in either environmental state at the end of ontogeny. Thus, to compute rewards and penalties, we need to compute the expectation across both environmental states, weighted by how likely each state is as indicated by the posterior estimates at the end of ontogeny. We denote the mapping from phenotypic increments to rewards and penalties by $f(y)$, where y can refer to both y_0 and y_1, and derive the following expressions for expected rewards and penalties:

$$\phi(Y_{mat}) = P(E_0|D_{t=T}) \bullet f(y_0) + P(E_1|D_{t=T}) \bullet f(y_1)$$
$$\psi(Y_{mat}) = -\left(P(E_0|D_{t=T}) \bullet f(y_1) + P(E_1|D_{t=T}) \bullet f(y_0)\right)$$

Lastly, we present the three functional mappings between the realized phenotype and fitness rewards and penalties:

Returns on fitness - $f(y)$	Formula	Parameter settings to ensure that maximal rewards and penalties correspond to $T-1$
linear	$f(y) = y$	-
diminishing	$f(y) = \alpha(1 - e^{-\beta y})$	$\beta = 0.2, \alpha = \frac{T-1}{1 - e^{-\beta(T-1)}}$
increasing	$f(y) = \alpha(e^{\beta y} - 1)$	$\beta = 0.2, \alpha = \frac{T-1}{e^{\beta(T-1)-1}}$

d) Optimal decisions

In each time period, a developing organism can choose one of three options: increment one step on P_0 , one step on P_1 or wait and forgo specialization. It chooses the option with the highest expected fitness at the end of ontogeny at $t = T$. In the event of a tie between two or all of the options the organism chooses amongst the current alternatives with equal probability.

$F(D_t, y_0, y_1, y_w, t, T)$ denotes the maximum expected fitness that can be attained as a result of decisions made between t and T, when the organism's current state after the last cue sampled is (D_t, y_0, y_1, y_w, T) and the organisms chooses option a, so that:

$$F(D_t, y_0, y_1, y_w, t, T) = \max_{a \epsilon \{0,1,w\}} F_a \text{, where}$$

$$F_0 = F(D_t, y_0 + 1, y_1, y_w, t + 1, T),$$
$$F_1 = F(D_t, y_0, y_1 + 1, y_w, t + 1, T),$$
$$F_w = F(D_t, y_0, y_1, y_w + 1, t + 1, T).$$

We apply backwards induction to solve the dynamic programming equation $F(D_t, y_0, y_1, y_w, t, T)$ for all t. We start with $t = T$:

$$F(D_t, y_0, y_1, y_w, T, T) = \pi(y_0, y_1, T).$$

After calculating expected fitness at the end of ontogeny we continue by decrementing t. For each $t < T$ we compute the a, which maximizes $F(D_t, y_0, y_1, y_w, t + 1, T)$ in time period t.

SM 3 - Bayesian inference visualized

The following figures show how the posterior estimate is updated across ontogeny when organisms receive cues that follow different cue reliability patterns (either fixed across ontogeny or variable). In relation to our model the plotted trajectories of posterior estimates correspond to an organism who encounters cues to the correct environment at each time point. We calculate posterior estimates according to Bayes' theorem, where $P(E_0)$ and $P(E_1)$ correspond to the initial priors for the possible environmental states and $P(C|E_0)$ corresponds to the cue reliability, and $P(E_0|C)$ and $P(E_1|C)$ to the updated posteriors:

$$P(E_0|C) = \frac{P(C|E_0) \bullet P(E_0)}{P(C|E_0) \bullet P(E_0) + (1 - P(C|E_0)) \bullet P(E_1)},$$

$$P(E_1|C) = 1 - P(E_0|C).$$

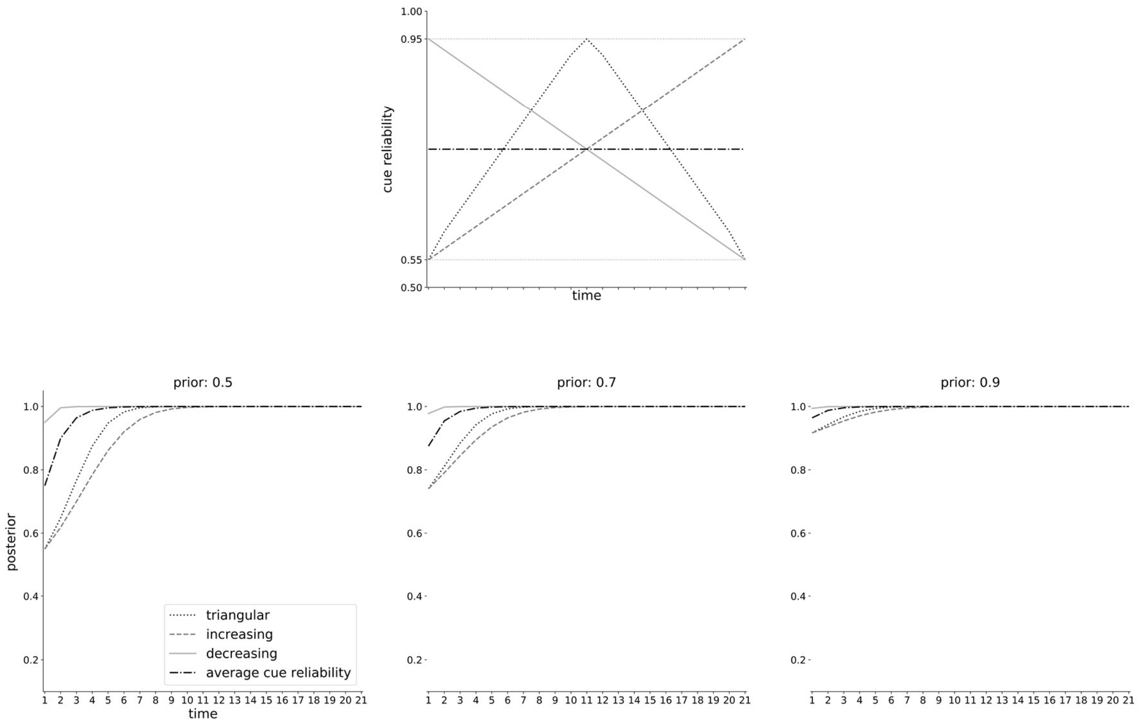

Figure A3.1. Bayesian updating for a prior of 0.5 and priors that are congruent with the true environmental state (i.e. 0.7 and 0.9) when cue reliabilities vary (with a maximum of 0.95) and for a fixed cue reliability. The top panel shows the three varying cue reliability patterns as well as the average cue reliability pattern, i.e., a fixed value of 0.75. The bottom three panels show how the posterior develops across time following Bayesian updating with.

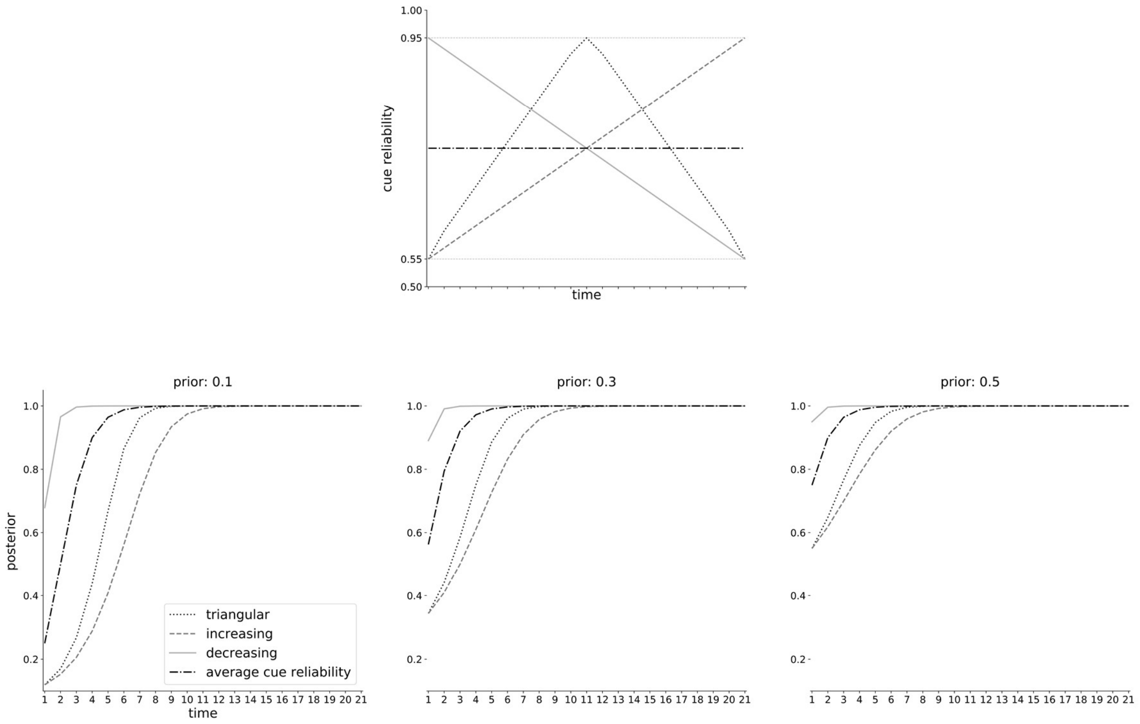

Figure A3.2. Bayesian updating for a prior of 0.5 and priors that are incongruent with the true environmental state (i.e. 0.1 and 0.3) when cue reliabilities vary (with a maximum of 0.95) and for a fixed cue reliability. The top panel shows the three varying cue reliability patterns as well as the average cue reliability pattern, i.e., a fixed value of 0.75. The bottom three panels show how the posterior develops across time following Bayesian updating with.

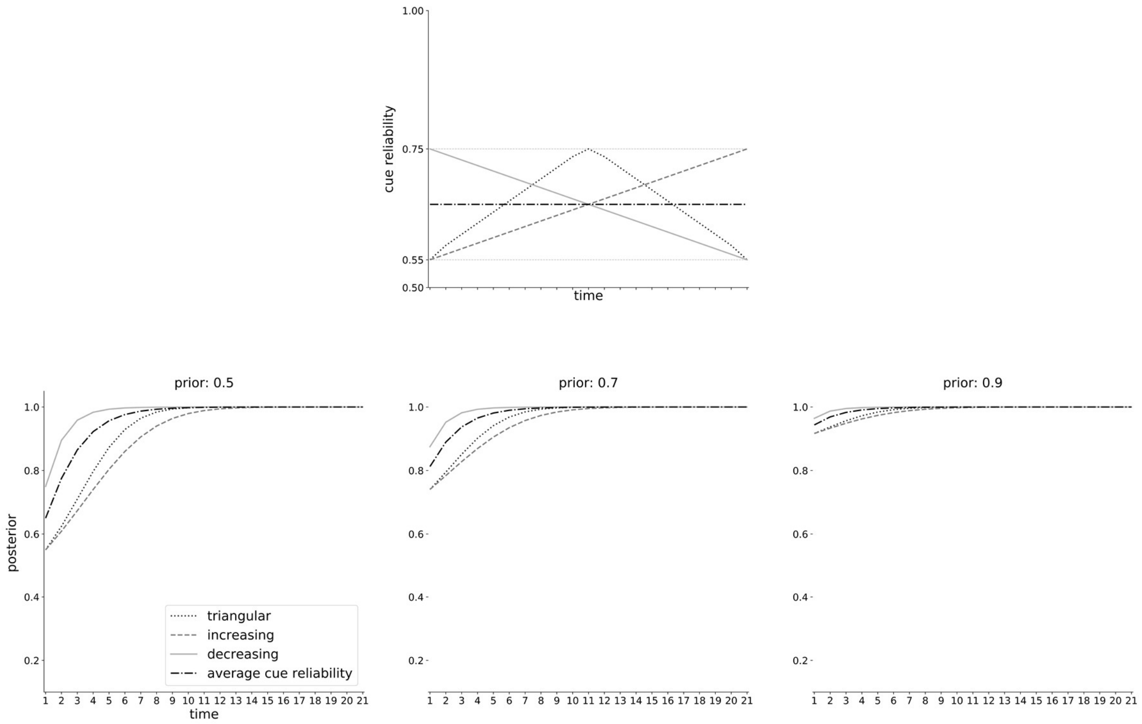

Figure A3.3. Bayesian updating for a prior of 0.5 and priors that are congruent with the true environmental state (i.e. 0.7 and 0.9) when cue reliabilities vary (with a maximum of 0.75) and for a fixed cue reliability. The top panel shows the three varying cue reliability patterns as well as the average cue reliability pattern, i.e., a fixed value of 0.65. The bottom three panels show how the posterior develops across time following Bayesian updating with.

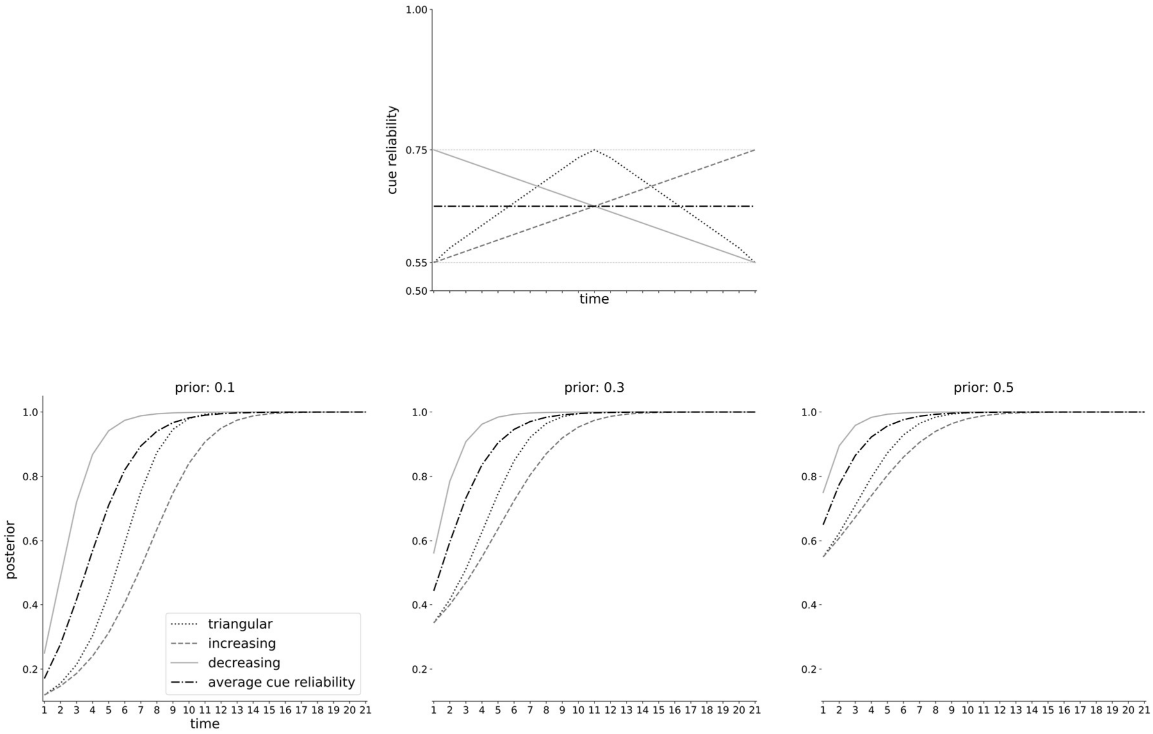

Figure A3.4. Bayesian updating for a prior of 0.5 and priors that are incongruent with the true environmental state (i.e. 0.1 and 0.3) when cue reliabilities vary (with a maximum of 0.75) and for a fixed cue reliability. The top panel shows the three varying cue reliability patterns as well as the average cue reliability pattern, i.e., a fixed value of 0.65. The bottom three panels show how the posterior develops across time following Bayesian updating with.

SM 4 – Measuring changes in plasticity across ontogeny

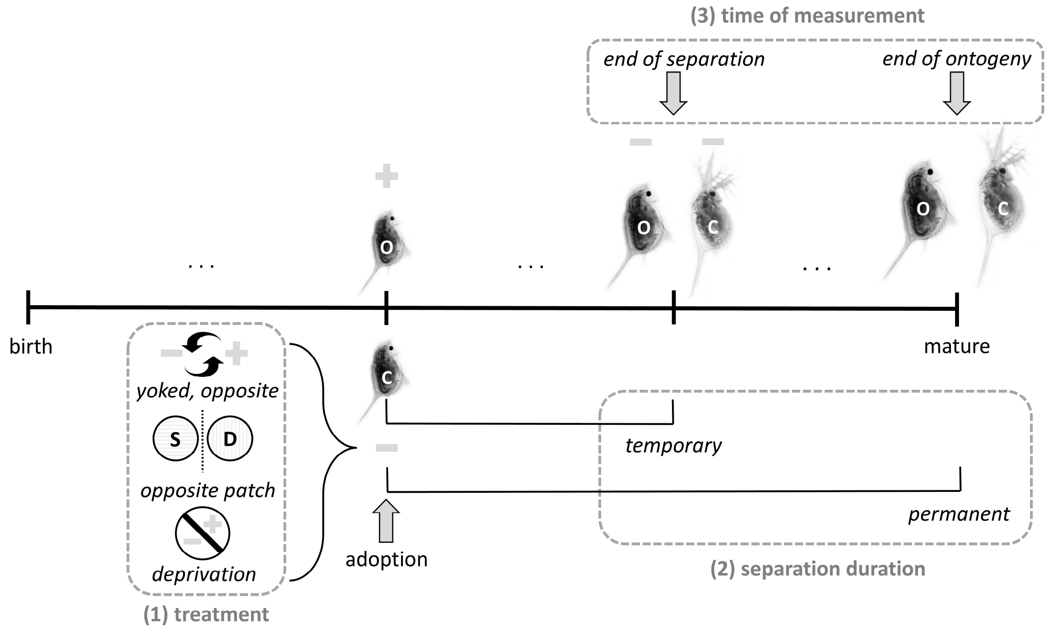

Figure A4.1 Measuring changes in plasticity across ontogeny (adapted from Frankenhuis & Walasek, 2020[2]). We separate twins (original, denoted with O, and clone, denoted with C) at different ages. We vary three dimensions: treatment, separation duration, and time of measurement. (1) Treatment refers to how the experiences of the original and clone differ during their separation. The clone might experience yoked, opposite cues; cues from the opposite patch; or deprivation. With yoked opposite cues, the clone always samples the opposite cue of the original: if the original samples a minus cue [-], the clone samples a plus cue [+]. With cues from the opposite patch, the clone samples a sequence of cues typical of the opposite patch: if the original tends to sample more minus cues, the clone tends to sample more plus cues. In our figure, the original and the clone are both in the dangerous patch (denoted with D), but the clone receives cues typical of the safe patch (denoted with S). With deprivation, the clone is equally likely to sample a plus or a minus cue; thus preventing learning about the environment. (2) Separation duration refers to whether the separation of twins is permanent or temporary. Permanent separation occurs if twins experience different conditions from their separation until the end of ontogeny (maturity). Temporary separation occurs if twins are reunited before the end of ontogeny. (3) Time of measurement refers to when differences in the phenotypes of twins are measured. We measure differences in phenotypes of twins at two different time points: at the end of their separation and at the end of ontogeny. Our results show that different treatments tend to produce (qualitatively) similar patterns of plasticity. Our predictions are therefore similar for different treatments and for different measurement times used in empirical research. Copyright: we have used the images of Daphnia with permission from Dr. Linda Weiss (2018).

Note, that we have changed the term “yoked” to “reciprocal” in the remainder of the SM and the main manuscript. We left the original figure and legend unaltered compared to its original source.

[2] Frankenhuis WE, Walasek N. 2020 Modeling the evolution of sensitive periods. *Dev. Cogn. Neurosci.* **41**, 100715. (doi:10.1016/j.dcn.2019.100715)

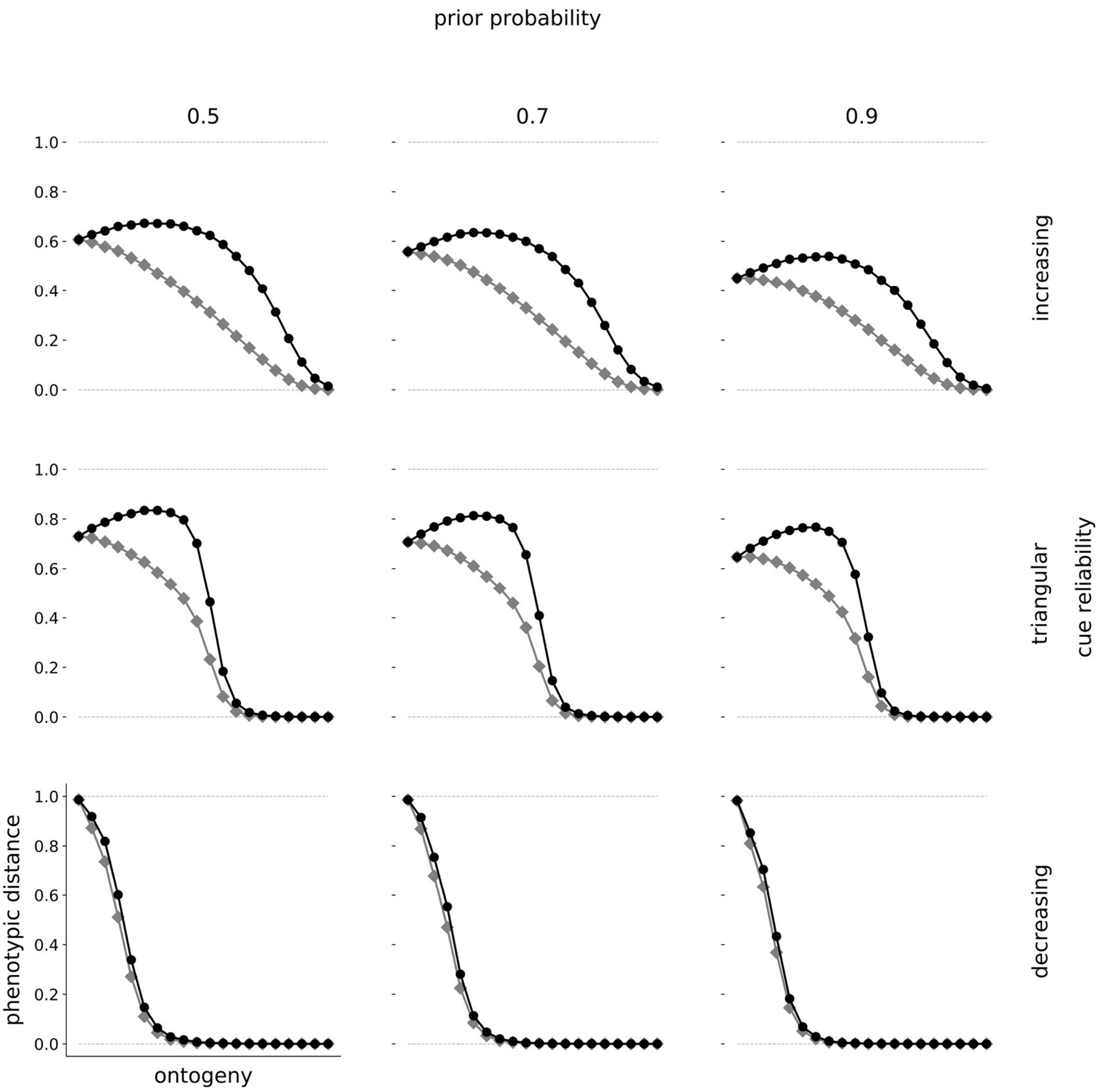

Figure A4.2. Plasticity across ontogeny – opposite patch cues. The fitness rewards for correct specializations and fitness penalties for incorrect specializations are linear across all panels. The prior probability of E_1 varies across columns and the cue reliability pattern varies across rows. Each panel represents T experimental 'twin studies', one for each $t \in \{1, T\}$. Outcomes of each twin study are marked by a grey diamond and a black circle. For each study we simulate 10,000 pairs of identical twins who follow the optimal policy and track their development across ontogeny. The environmental state is fixed to E_1. For each pair of twins, one individual (the 'focal') receives a set of environmental cues across ontogeny simulated from the prior probability and cue reliability pattern. Its clone receives the same cues until the moment of separation in time period t after which it begins to receive cues indicating the opposite patch, which lasts until the end of ontogeny. The vertical axis within each panel depicts the phenotypic distance between focal individuals and their clones. The horizontal axis depicts the time period in which pairs of twins were separated. The phenotypic distance at the end of ontogeny between a focal individual and its clone corresponds to the Euclidean distance between their phenotypes. Grey lines and diamonds depict 'absolute' phenotypic distance, the average distance between the 10,000 focal individuals and their clones at the end of ontogeny (ranging from 0 to $20\sqrt{2}$, scaled to a 0 to 1 range). Black lines and circles depict 'proportional' distance, the average absolute distance divided by the maximum possible distance following separation.

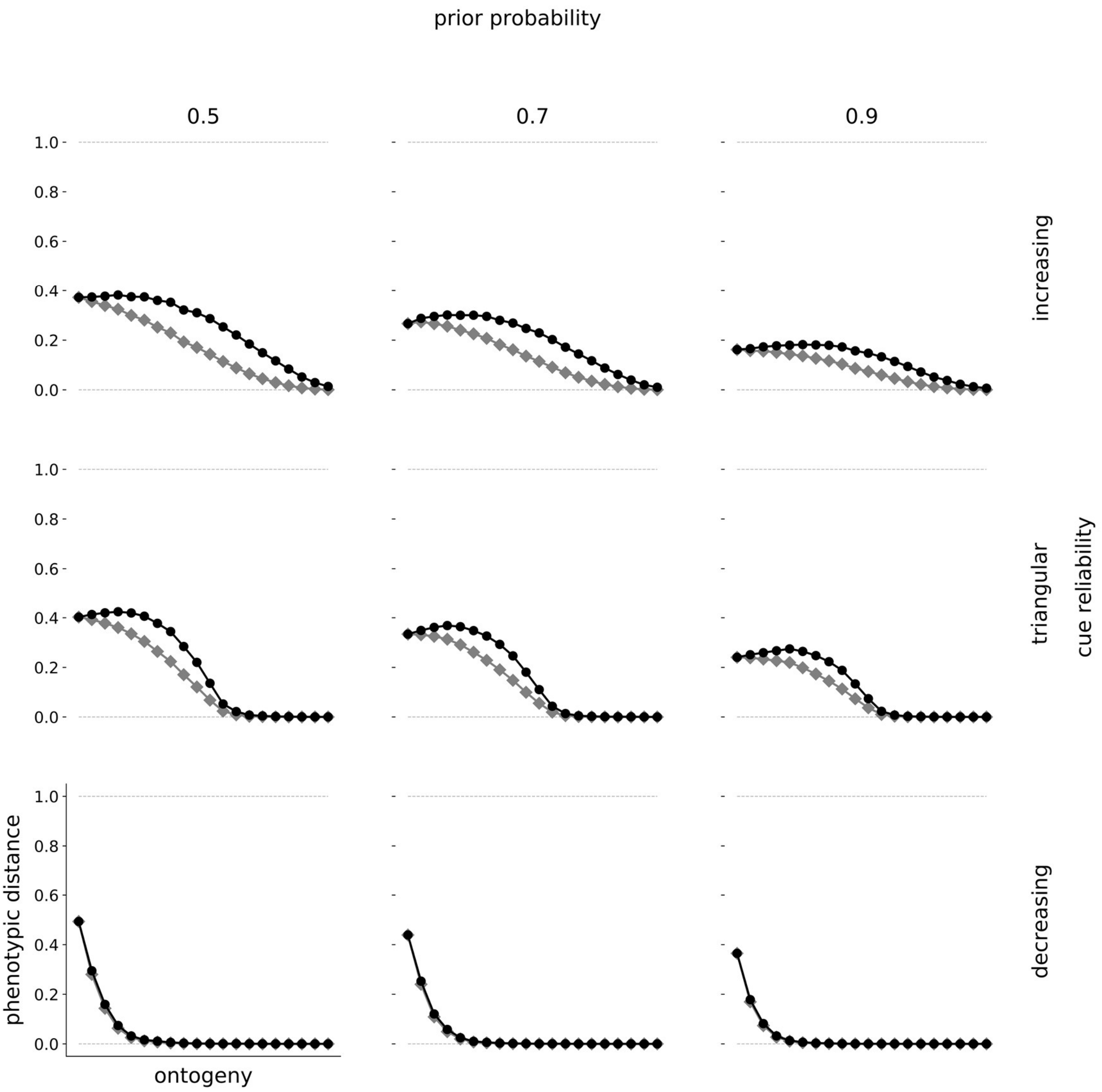

Figure A4.3. Plasticity across ontogeny - deprivation. The fitness rewards for correct specializations and fitness penalties for incorrect specializations are linear across all panels. The prior probability of E_1 varies across columns and the cue reliability pattern varies across rows. Each panel represents T experimental 'twin studies', one for each $t \in \{1, T\}$. Outcomes of each twin study are marked by a grey diamond and a black circle. For each study we simulate 10,000 pairs of identical twins who follow the optimal policy and track their development across ontogeny. The environmental state is fixed to E_1. For each pair of twins, one individual (the 'focal') receives a set of environmental cues across ontogeny simulated from the prior probability and cue reliability pattern. Its clone receives the same cues until the moment of separation in time period t after which it begins to experience deprivation, meaning that cues from both environmental states are equally likely, which lasts until the end of ontogeny. The vertical axis within each panel depicts the phenotypic distance between focal individuals and their clones. The horizontal axis depicts the time period in which pairs of twins were separated. The phenotypic distance at the end of ontogeny between a focal individual and its clone corresponds to the Euclidean distance between their phenotypes. Grey lines and diamonds depict 'absolute' phenotypic distance, the average distance between the 10,000 focal individuals and their clones at the end of ontogeny (ranging from 0 to $20\sqrt{2}$, scaled to a 0 to 1 range). Black lines and circles depict 'proportional' distance, the average absolute distance divided by the maximum possible distance following separation.

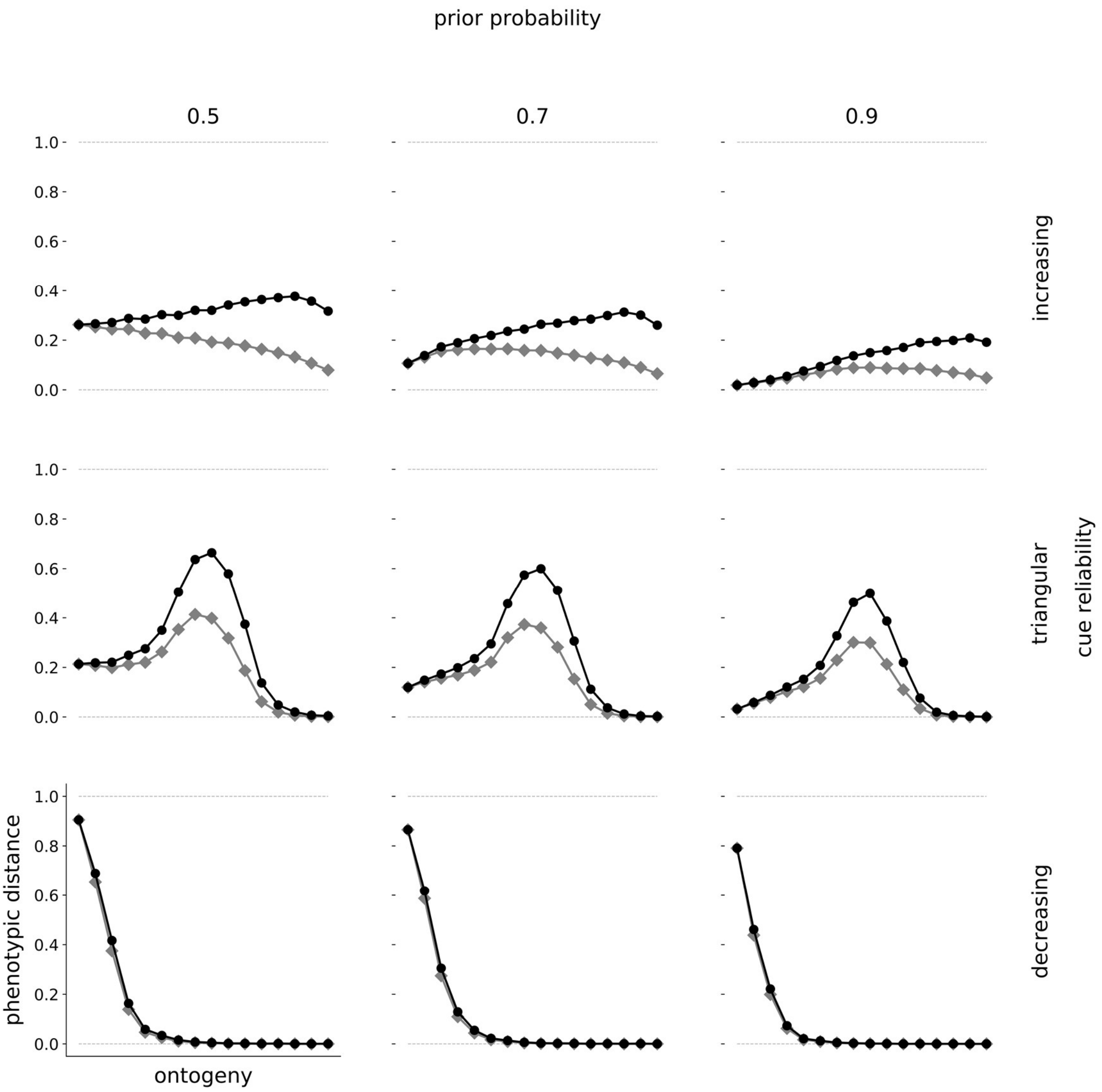

Figure A4.4. Plasticity across ontogeny – temporary separation and time of measurement is at the end of ontogeny. The fitness rewards for correct specializations and fitness penalties for incorrect specializations are linear across all panels. The prior probability of E_1 varies across columns and the cue reliability pattern varies across rows. Each panel represents T experimental 'twin studies', one for each $t \in \{1, T\}$. Outcomes of each twin study are marked by a grey diamond and a black circle. For each study we simulate 10,000 pairs of identical twins who follow the optimal policy and track their development across ontogeny. The environmental state is fixed to E_1. For each pair of twins, one individual (the 'focal') receives a set of environmental cues across ontogeny simulated from the prior probability and cue reliability pattern. Its clone receives the same cues until the moment of separation in time period t after which it begins to receive reciprocal, opposite cues, which lasts temporarily (for 5 discrete time points) before twins continue development together until the end of ontogeny. The vertical axis within each panel depicts the phenotypic distance between focal individuals and their clones. The horizontal axis depicts the time period in which pairs of twins were separated. The phenotypic distance at the end of ontogeny between a focal individual and its clone corresponds to the Euclidean distance between their phenotypes. Grey lines and diamonds depict 'absolute' phenotypic distance, the average distance between the 10,000 focal individuals and their clones at the end of ontogeny (ranging from 0 to $20\sqrt{2}$, scaled to a 0 to 1 range). Black lines and circles depict 'proportional' distance, the average absolute distance divided by the maximum possible distance following separation.

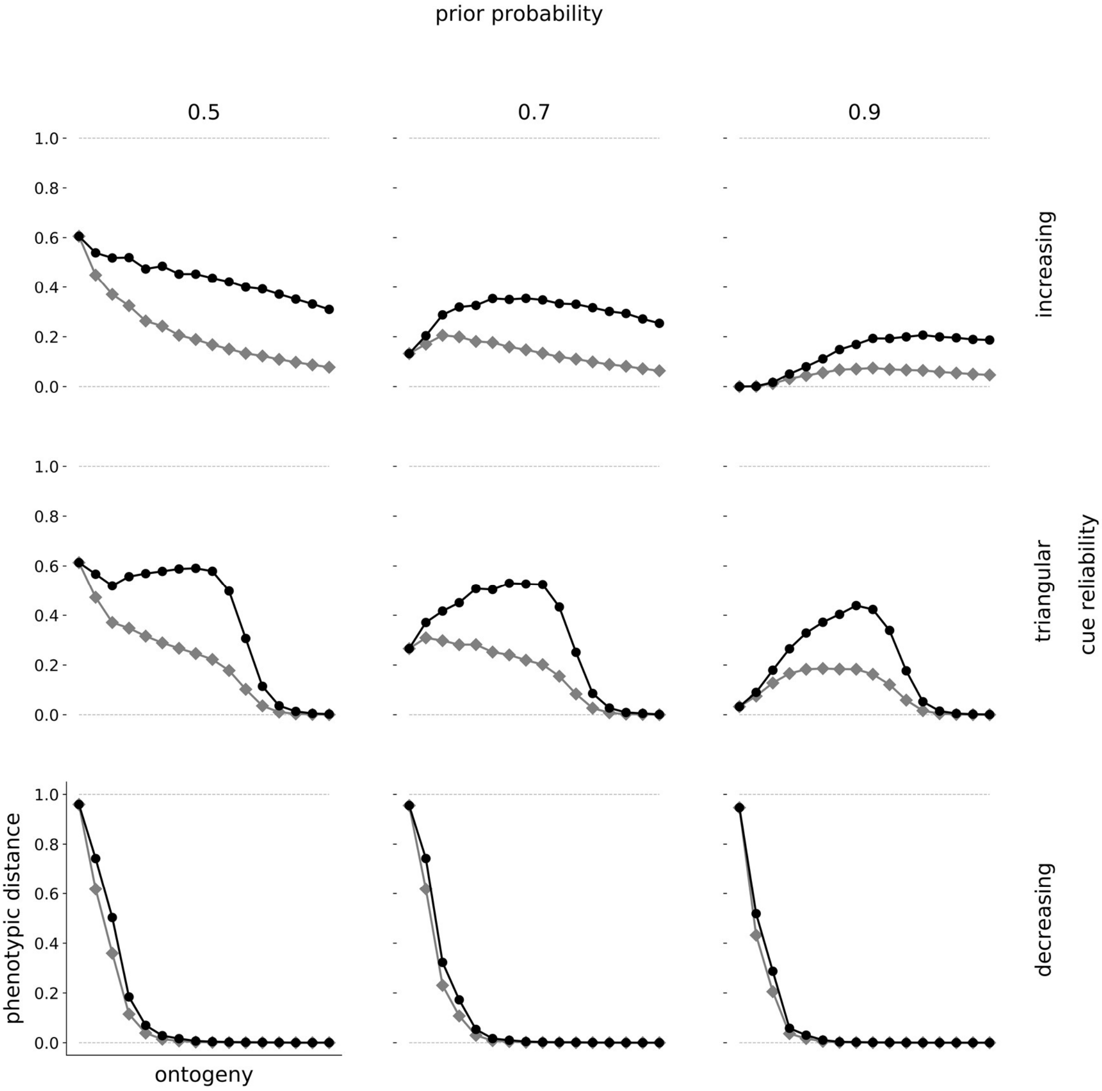

Figure A4.5. Plasticity across ontogeny – temporary separation and time of measurement is at the end of the separation. The fitness rewards for correct specializations and fitness penalties for incorrect specializations are linear across all panels. The prior probability of E_1 varies across columns and the cue reliability pattern varies across rows. Each panel represents T experimental 'twin studies', one for each $t \in \{1, T\}$. Outcomes of each twin study are marked by a grey diamond and a black circle. For each study we simulate 10,000 pairs of identical twins who follow the optimal policy and track their development across ontogeny. The environmental state is fixed to E_1. For each pair of twins, one individual (the 'focal') receives a set of environmental cues across ontogeny simulated from the prior probability and cue reliability pattern. Its clone receives the same cues until the moment of separation in time period t after which it begins to receive reciprocal, opposite cues, which lasts temporarily (for 5 discrete time points) before twins continue development together until the end of ontogeny. The vertical axis within each panel depicts the phenotypic distance between focal individuals and their clones. The horizontal axis depicts the time period in which pairs of twins were separated. The phenotypic distance after the separation between a focal individual and its clone corresponds to the Euclidean distance between their phenotypes. Grey lines and diamonds depict 'absolute' phenotypic distance, the average distance between the 10,000 focal individuals and their clones after the separation (ranging from 0 to $20\sqrt{2}$, scaled to a 0 to 1 range). Black lines and circles depict 'proportional' distance, the average absolute distance divided by the maximum possible distance during the separation.

SM 5 – Main plots for all penalty and reward functions (10 time steps)

With varying cue reliabilities the state space increases exponentially, which also exponentially increases computation time. Therefore, we have presented our main findings for 20 time steps but show results for other parameter settings for 10 time steps. This restriction does not qualitatively change our results.

a) Plasticity across ontogeny

Linear rewards & linear penalties

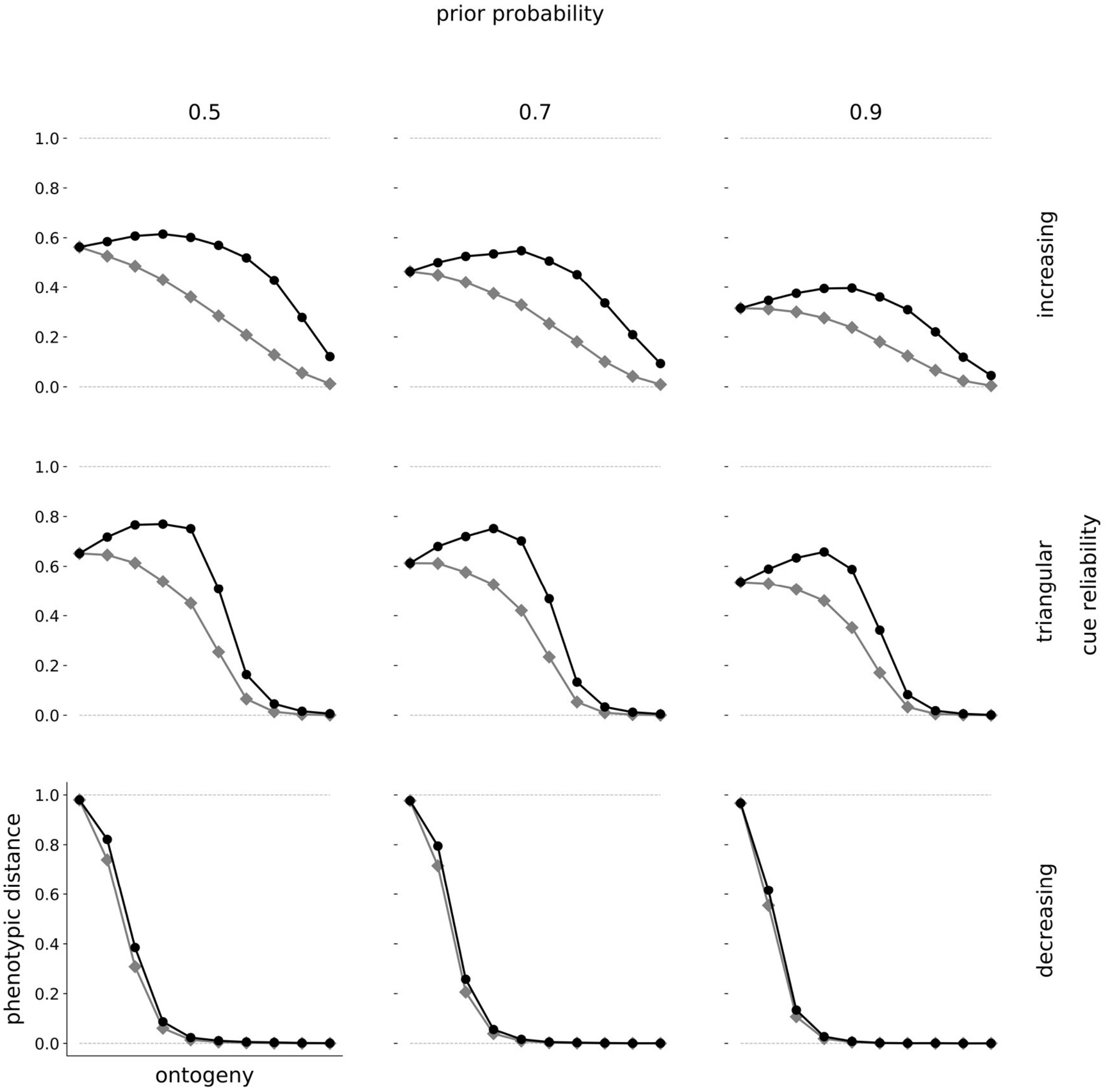

Figure A5.1. Plasticity across ontogeny. The fitness rewards for correct specializations are **linear** and fitness penalties for incorrect specializations are **linear** across all panels. The prior probability of E_1 varies across columns and the cue reliability pattern varies across rows. Each panel represents T experimental 'twin studies', one for each $t \in \{1, T\}$. Outcomes of each twin study are marked by a grey diamond and a black circle. For each study we simulate 10,000 pairs of identical twins who follow the optimal policy and track their development across ontogeny. The environmental state is fixed to E_1. For each pair of twins, one individual (the 'focal') receives a set of environmental cues across ontogeny simulated from the prior probability and cue reliability pattern. Its clone receives the same cues until the moment of separation in time period t after which it begins to receive reciprocal, opposite cues, which lasts until the end of ontogeny. The vertical axis within each panel depicts the phenotypic distance between focal individuals and their clones. The horizontal axis depicts the time period in which pairs of twins were separated. The phenotypic distance at the end of ontogeny between a focal individual and its clone corresponds to the Euclidean distance between their phenotypes. Grey lines and diamonds depict 'absolute' phenotypic distance, the average distance between the 10,000 focal individuals and their clones at the end of ontogeny (ranging from 0 to $20\sqrt{2}$, scaled to a 0 to 1 range). Black lines and circles depict 'proportional' distance, the average absolute distance divided by the maximum possible distance following separation.

Linear reward & increasing penalties

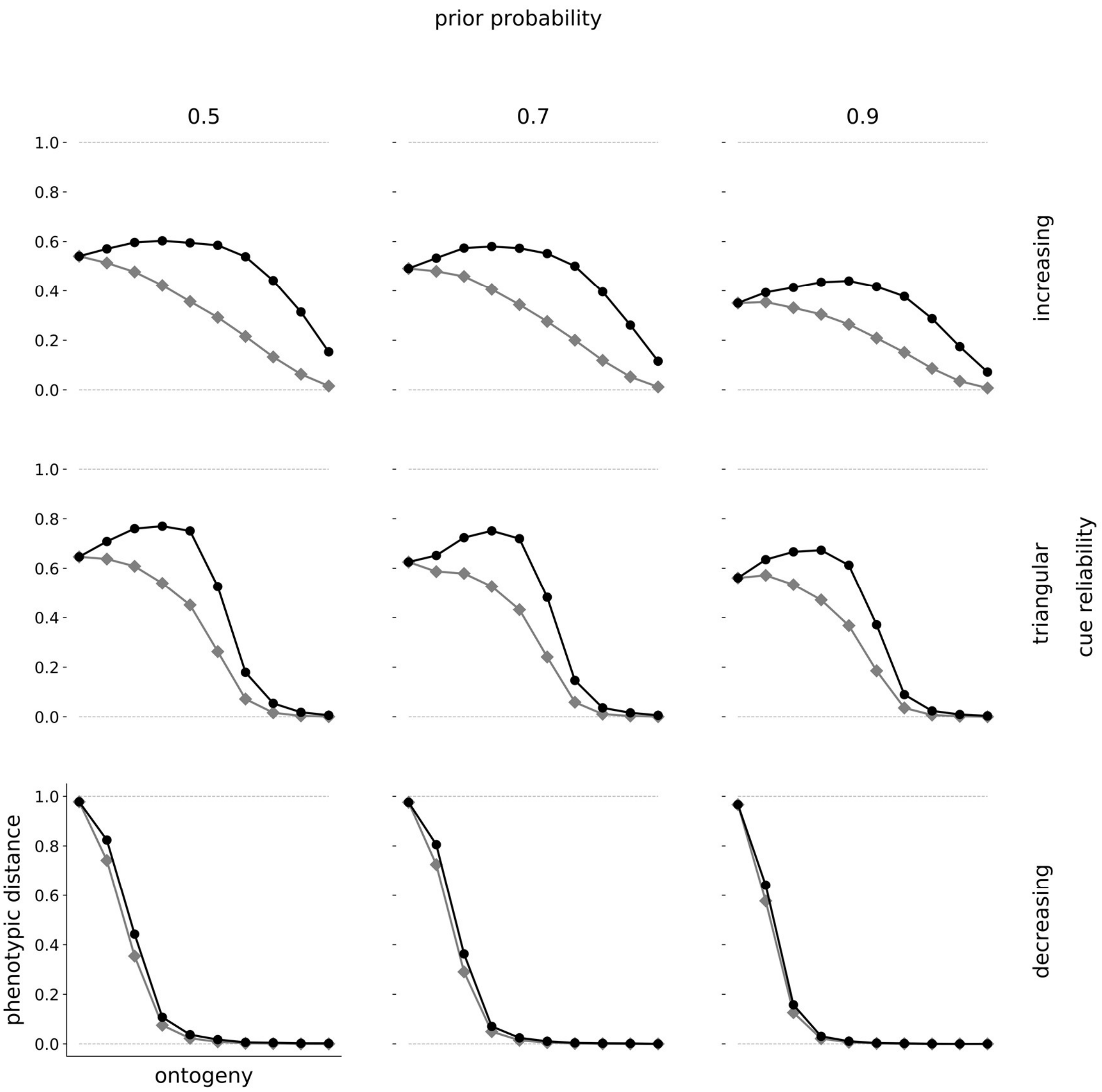

Figure A5.2. Plasticity across ontogeny. The fitness rewards for correct specializations are **linear** and fitness penalties for incorrect specializations are **increasing** across all panels. The prior probability of E_1 varies across columns and the cue reliability pattern varies across rows. Each panel represents T experimental 'twin studies', one for each $t \in \{1, T\}$. Outcomes of each twin study are marked by a grey diamond and a black circle. For each study we simulate 10,000 pairs of identical twins who follow the optimal policy and track their development across ontogeny. The environmental state is fixed to E_1. For each pair of twins, one individual (the 'focal') receives a set of environmental cues across ontogeny simulated from the prior probability and cue reliability pattern. Its clone receives the same cues until the moment of separation in time period t after which it begins to receive reciprocal, opposite cues, which lasts until the end of ontogeny. The vertical axis within each panel depicts the phenotypic distance between focal individuals and their clones. The horizontal axis depicts the time period in which pairs of twins were separated. The phenotypic distance at the end of ontogeny between a focal individual and its clone corresponds to the Euclidean distance between their phenotypes. Grey lines and diamonds depict 'absolute' phenotypic distance, the average distance between the 10,000 focal individuals and their clones at the end of ontogeny (ranging from 0 to $20\sqrt{2}$, scaled to a 0 to 1 range). Black lines and circles depict 'proportional' distance, the average absolute distance divided by the maximum possible distance following separation.

Linear rewards & diminishing penalties

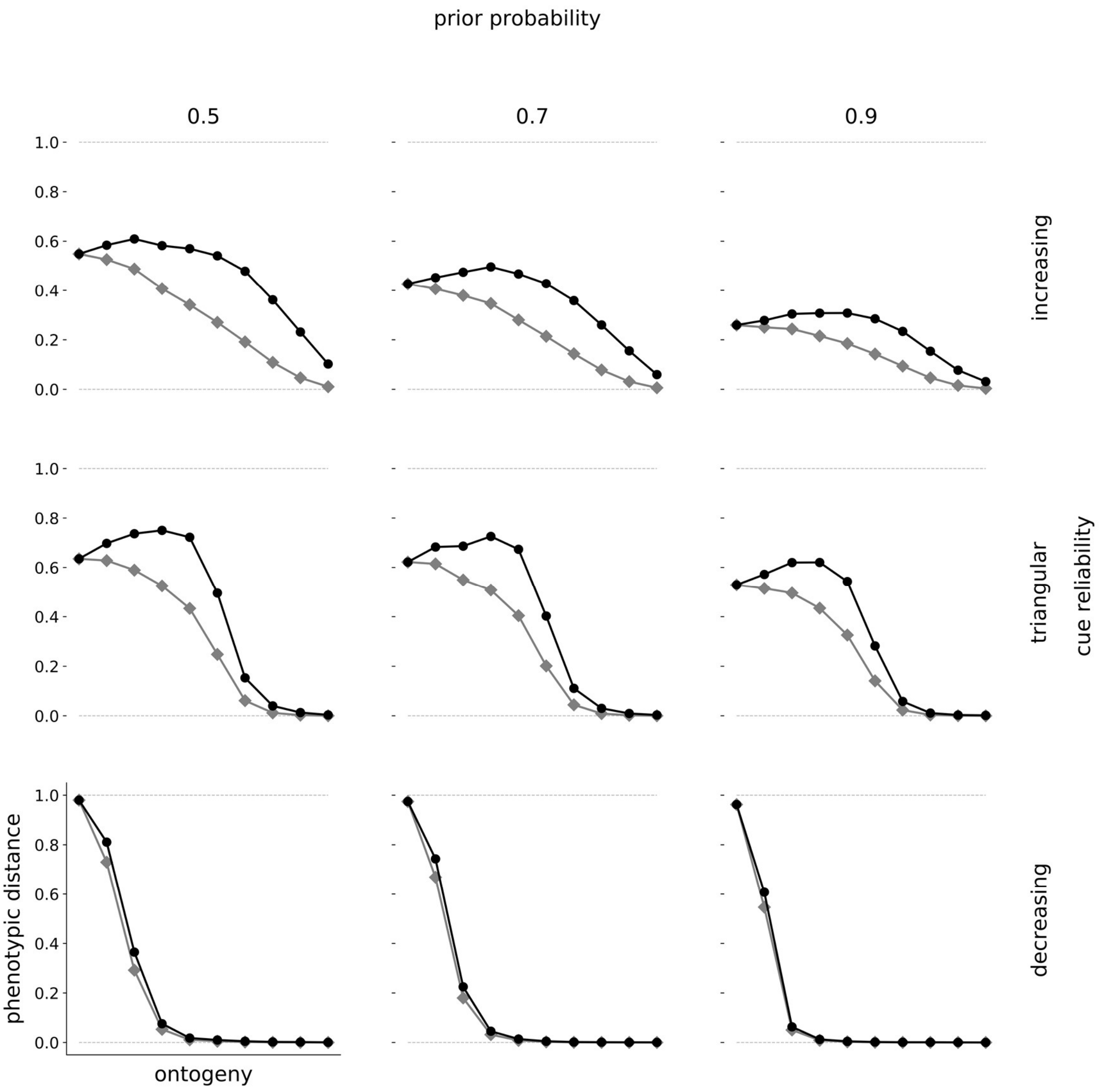

Figure A5.3. Plasticity across ontogeny. The fitness rewards for correct specializations are **diminishing** and fitness penalties for incorrect specializations are **linear** across all panels. The prior probability of E_1 varies across columns and the cue reliability pattern varies across rows. Each panel represents T experimental 'twin studies', one for each $t \in \{1, T\}$. Outcomes of each twin study are marked by a grey diamond and a black circle. For each study we simulate 10,000 pairs of identical twins who follow the optimal policy and track their development across ontogeny. The environmental state is fixed to E_1. For each pair of twins, one individual (the 'focal') receives a set of environmental cues across ontogeny simulated from the prior probability and cue reliability pattern. Its clone receives the same cues until the moment of separation in time period t after which it begins to receive reciprocal, opposite cues, which lasts until the end of ontogeny. The vertical axis within each panel depicts the phenotypic distance between focal individuals and their clones. The horizontal axis depicts the time period in which pairs of twins were separated. The phenotypic distance at the end of ontogeny between a focal individual and its clone corresponds to the Euclidean distance between their phenotypes. Grey lines and diamonds depict 'absolute' phenotypic distance, the average distance between the 10,000 focal individuals and their clones at the end of ontogeny (ranging from 0 to $20\sqrt{2}$, scaled to a 0 to 1 range). Black lines and circles depict 'proportional' distance, the average absolute distance divided by the maximum possible distance following separation.

Increasing rewards & linear penalties

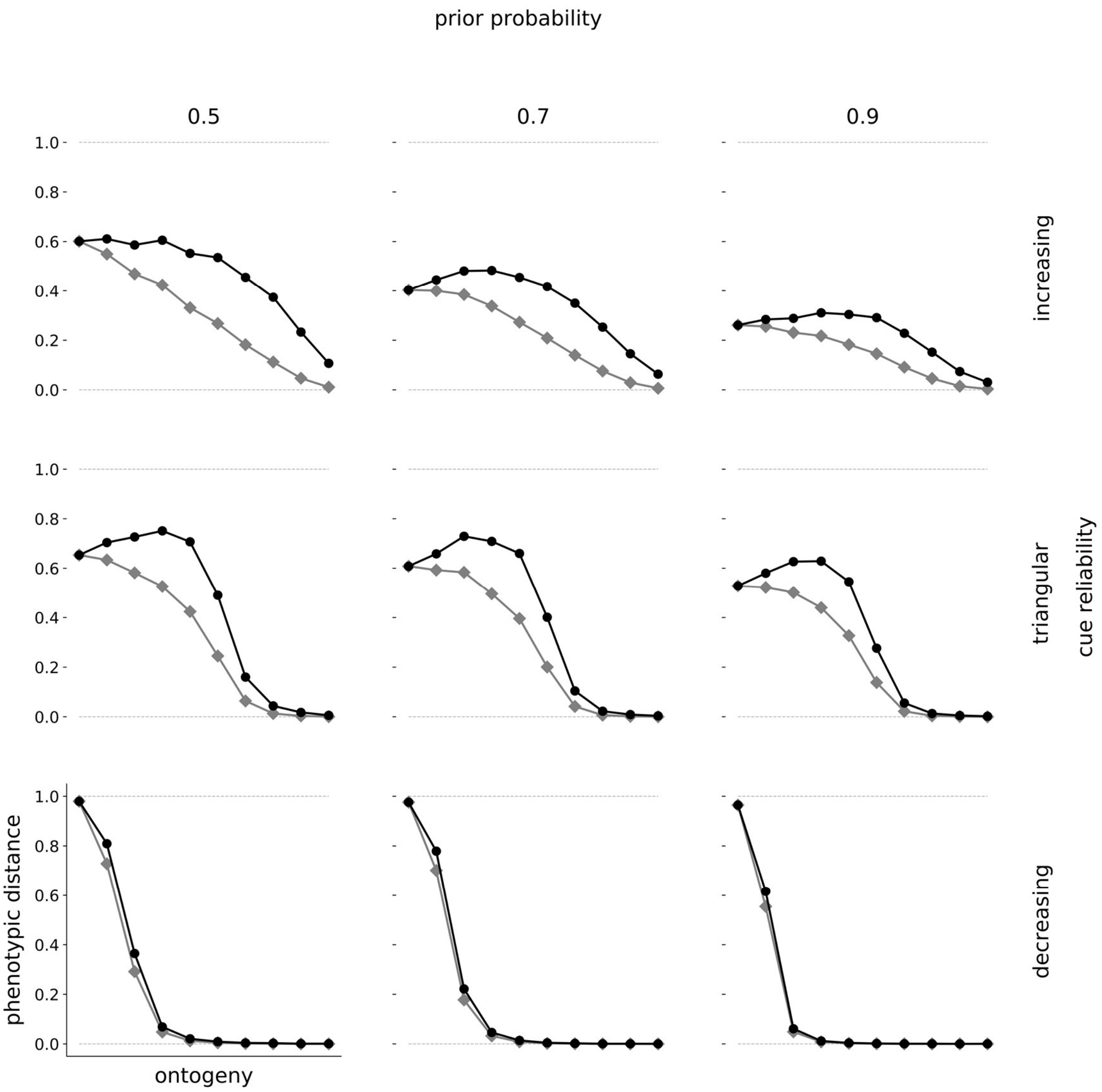

Figure A5.4. Plasticity across ontogeny. The fitness rewards for correct specializations are **increasing** and fitness penalties for incorrect specializations are **linear** across all panels. The prior probability of E_1 varies across columns and the cue reliability pattern varies across rows. Each panel represents T experimental 'twin studies', one for each $t \in \{1, T\}$. Outcomes of each twin study are marked by a grey diamond and a black circle. For each study we simulate 10,000 pairs of identical twins who follow the optimal policy and track their development across ontogeny. The environmental state is fixed to E_1. For each pair of twins, one individual (the 'focal') receives a set of environmental cues across ontogeny simulated from the prior probability and cue reliability pattern. Its clone receives the same cues until the moment of separation in time period t after which it begins to receive reciprocal, opposite cues, which lasts until the end of ontogeny. The vertical axis within each panel depicts the phenotypic distance between focal individuals and their clones. The horizontal axis depicts the time period in which pairs of twins were separated. The phenotypic distance at the end of ontogeny between a focal individual and its clone corresponds to the Euclidean distance between their phenotypes. Grey lines and diamonds depict 'absolute' phenotypic distance, the average distance between the 10,000 focal individuals and their clones at the end of ontogeny (ranging from 0 to $20\sqrt{2}$, scaled to a 0 to 1 range). Black lines and circles depict 'proportional' distance, the average absolute distance divided by the maximum possible distance following separation.

Increasing reward & increasing penalties

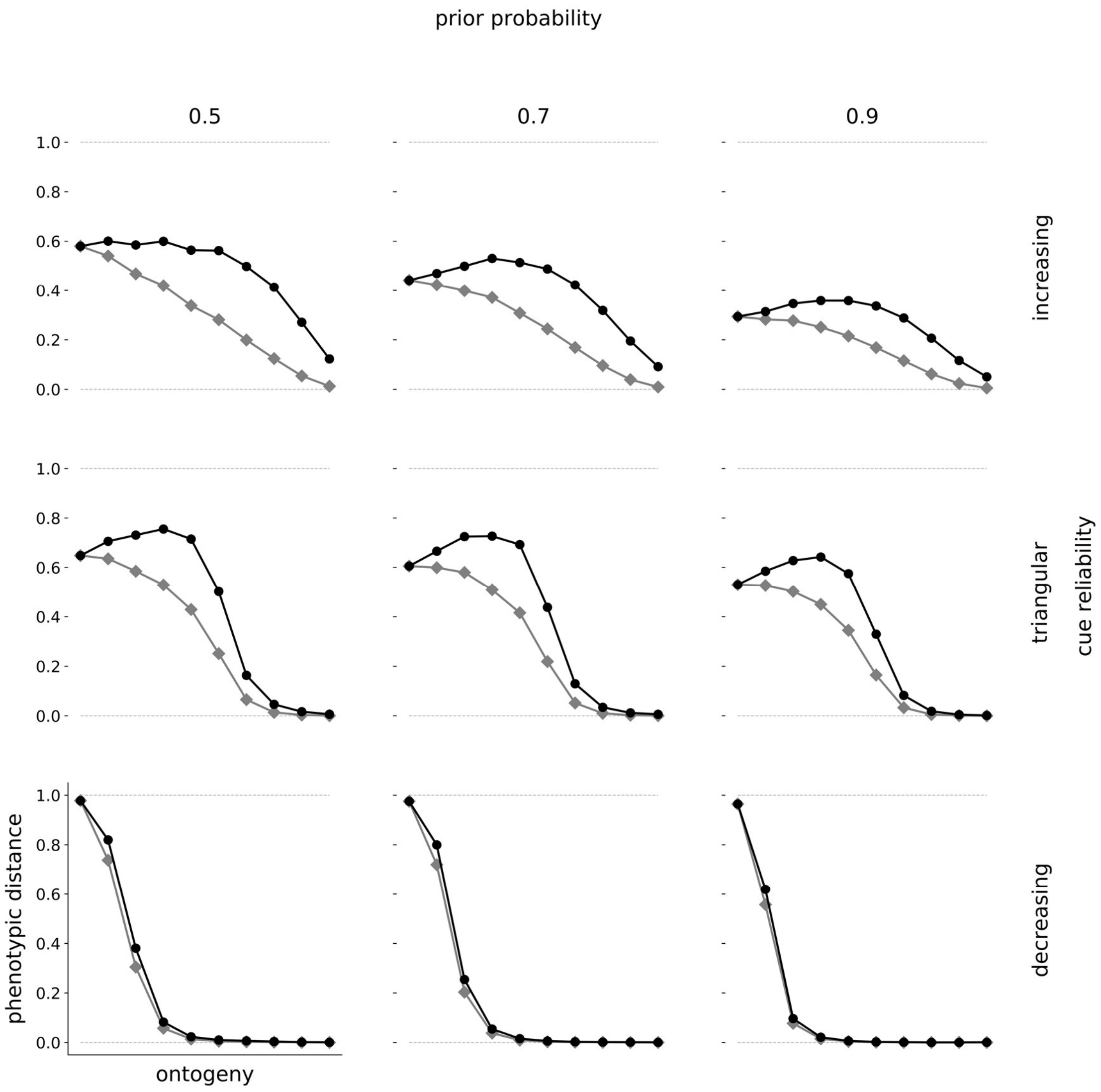

Figure A5.5. Plasticity across ontogeny. The fitness rewards for correct specializations are **increasing** and fitness penalties for incorrect specializations are **increasing** across all panels. The prior probability of E_1 varies across columns and the cue reliability pattern varies across rows. Each panel represents T experimental 'twin studies', one for each $t \in \{1, T\}$. Outcomes of each twin study are marked by a grey diamond and a black circle. For each study we simulate 10,000 pairs of identical twins who follow the optimal policy and track their development across ontogeny. The environmental state is fixed to E_1. For each pair of twins, one individual (the 'focal') receives a set of environmental cues across ontogeny simulated from the prior probability and cue reliability pattern. Its clone receives the same cues until the moment of separation in time period t after which it begins to receive reciprocal, opposite cues, which lasts until the end of ontogeny. The vertical axis within each panel depicts the phenotypic distance between focal individuals and their clones. The horizontal axis depicts the time period in which pairs of twins were separated. The phenotypic distance at the end of ontogeny between a focal individual and its clone corresponds to the Euclidean distance between their phenotypes. Grey lines and diamonds depict 'absolute' phenotypic distance, the average distance between the 10,000 focal individuals and their clones at the end of ontogeny (ranging from 0 to $20\sqrt{2}$, scaled to a 0 to 1 range). Black lines and circles depict 'proportional' distance, the average absolute distance divided by the maximum possible distance following separation.

Increasing rewards & diminishing penalties

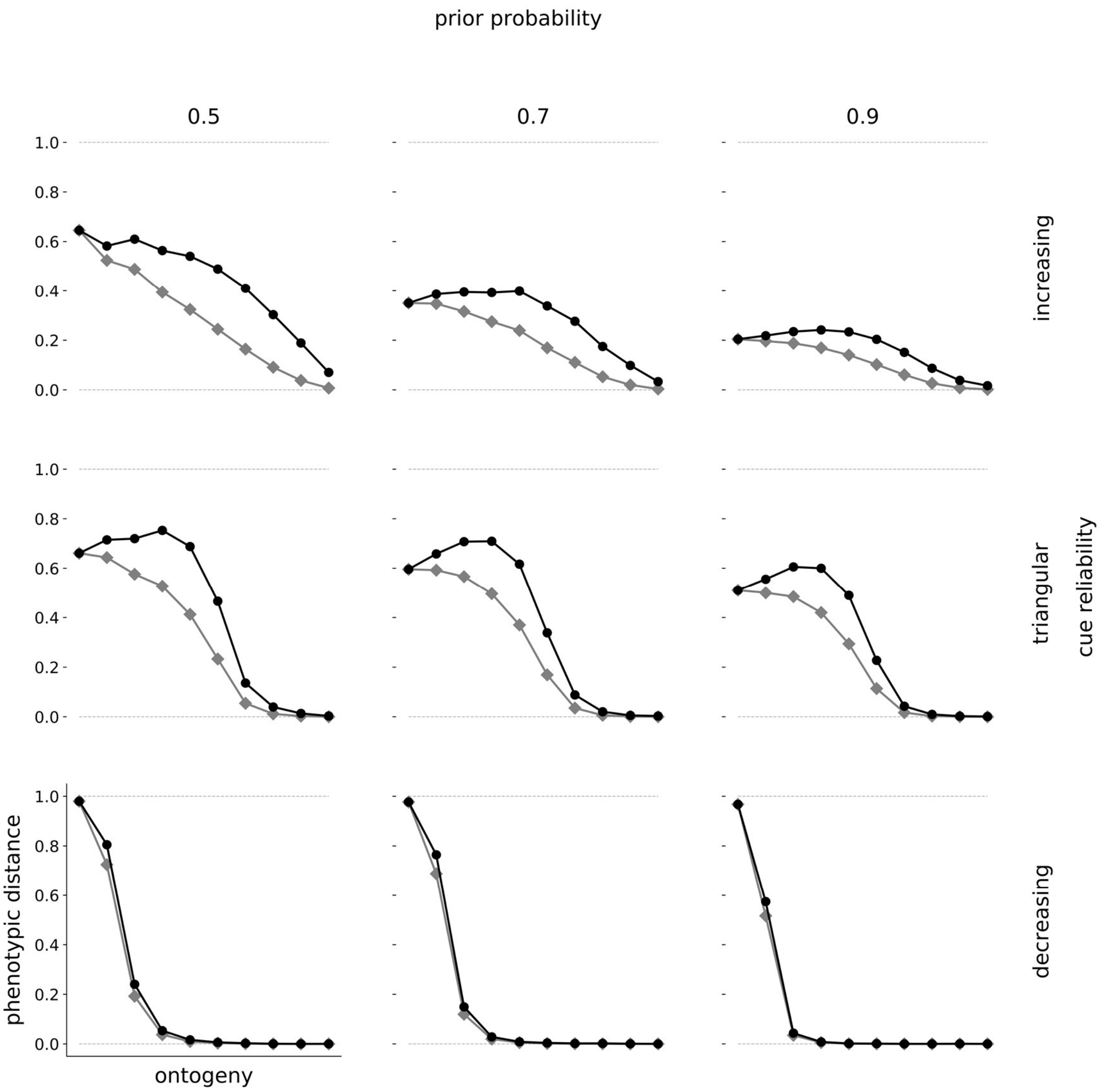

Figure A5.6. Plasticity across ontogeny. The fitness rewards for correct specializations are **increasing** and fitness penalties for incorrect specializations are **diminishing** across all panels. The prior probability of E_1 varies across columns and the cue reliability pattern varies across rows. Each panel represents T experimental 'twin studies', one for each $t \in \{1, T\}$. Outcomes of each twin study are marked by a grey diamond and a black circle. For each study we simulate 10,000 pairs of identical twins who follow the optimal policy and track their development across ontogeny. The environmental state is fixed to E_1. For each pair of twins, one individual (the 'focal') receives a set of environmental cues across ontogeny simulated from the prior probability and cue reliability pattern. Its clone receives the same cues until the moment of separation in time period t after which it begins to receive reciprocal, opposite cues, which lasts until the end of ontogeny. The vertical axis within each panel depicts the phenotypic distance between focal individuals and their clones. The horizontal axis depicts the time period in which pairs of twins were separated. The phenotypic distance at the end of ontogeny between a focal individual and its clone corresponds to the Euclidean distance between their phenotypes. Grey lines and diamonds depict 'absolute' phenotypic distance, the average distance between the 10,000 focal individuals and their clones at the end of ontogeny (ranging from 0 to $20\sqrt{2}$, scaled to a 0 to 1 range). Black lines and circles depict 'proportional' distance, the average absolute distance divided by the maximum possible distance following separation.

Diminishing rewards & linear penalties

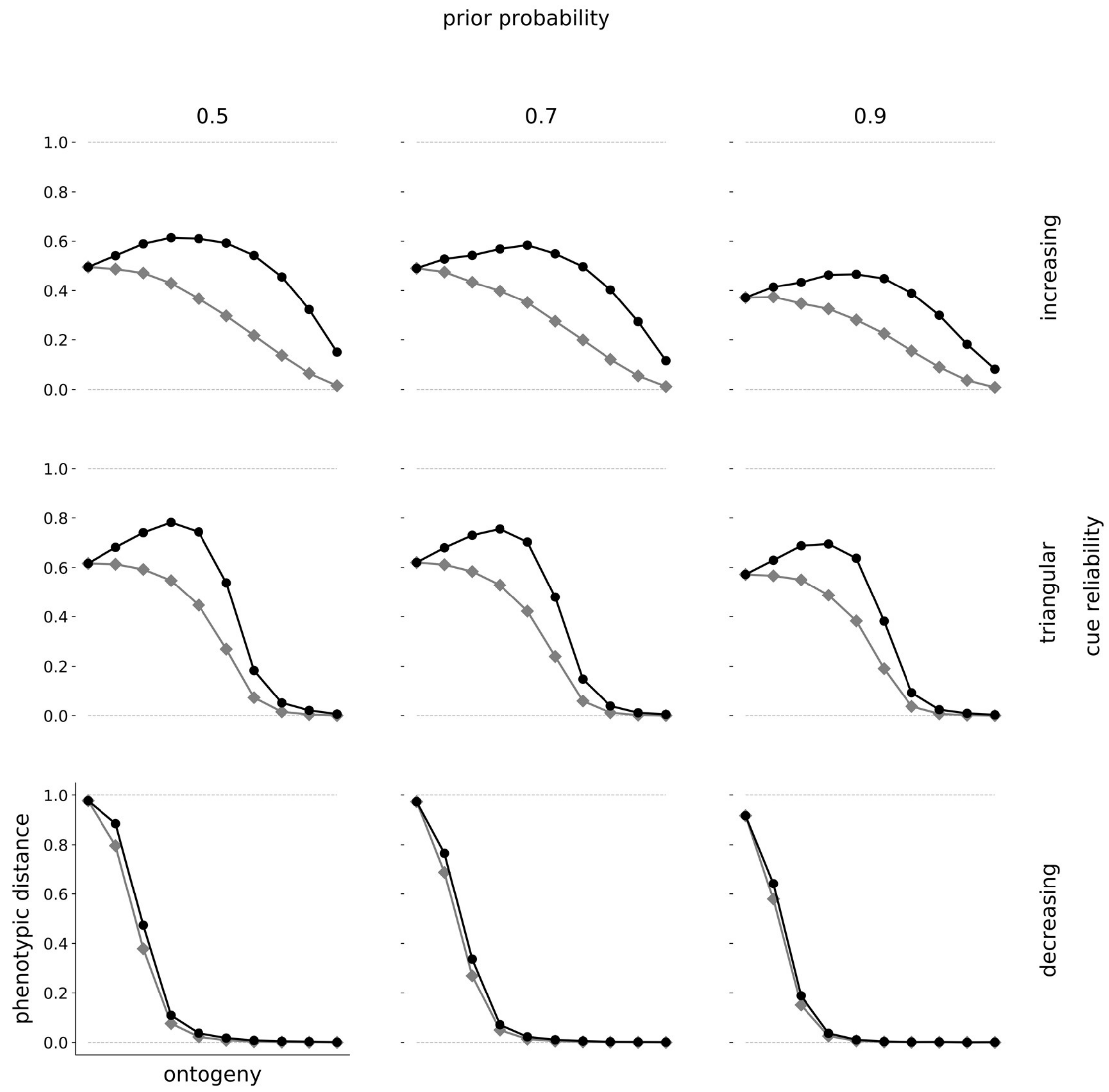

Figure A5.7. Plasticity across ontogeny. The fitness rewards for correct specializations are **diminishing** and fitness penalties for incorrect specializations are **linear** across all panels. The prior probability of E_1 varies across columns and the cue reliability pattern varies across rows. Each panel represents T experimental 'twin studies', one for each $t \in \{1, T\}$. Outcomes of each twin study are marked by a grey diamond and a black circle. For each study we simulate 10,000 pairs of identical twins who follow the optimal policy and track their development across ontogeny. The environmental state is fixed to E_1. For each pair of twins, one individual (the 'focal') receives a set of environmental cues across ontogeny simulated from the prior probability and cue reliability pattern. Its clone receives the same cues until the moment of separation in time period t after which it begins to receive reciprocal, opposite cues, which lasts until the end of ontogeny. The vertical axis within each panel depicts the phenotypic distance between focal individuals and their clones. The horizontal axis depicts the time period in which pairs of twins were separated. The phenotypic distance at the end of ontogeny between a focal individual and its clone corresponds to the Euclidean distance between their phenotypes. Grey lines and diamonds depict 'absolute' phenotypic distance, the average distance between the 10,000 focal individuals and their clones at the end of ontogeny (ranging from 0 to $20\sqrt{2}$, scaled to a 0 to 1 range). Black lines and circles depict 'proportional' distance, the average absolute distance divided by the maximum possible distance following separation.

Diminishing reward & increasing penalties

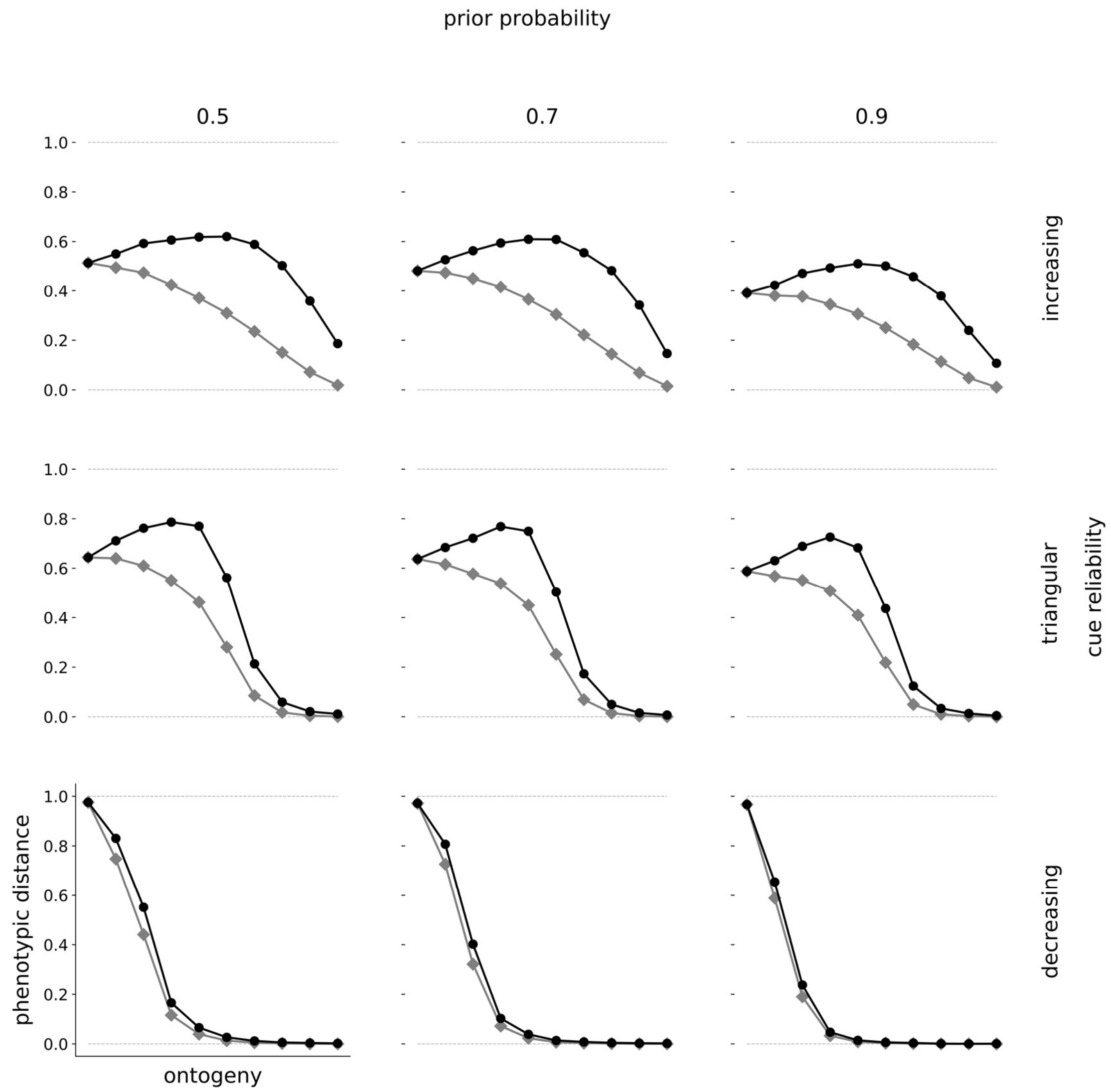

Figure A5.8. Plasticity across ontogeny. The fitness rewards for correct specializations are **diminishing** and fitness penalties for incorrect specializations are **increasing** across all panels. The prior probability of E_1 varies across columns and the cue reliability pattern varies across rows. Each panel represents T experimental 'twin studies', one for each $t \in \{1, T\}$. Outcomes of each twin study are marked by a grey diamond and a black circle. For each study we simulate 10,000 pairs of identical twins who follow the optimal policy and track their development across ontogeny. The environmental state is fixed to E_1. For each pair of twins, one individual (the 'focal') receives a set of environmental cues across ontogeny simulated from the prior probability and cue reliability pattern. Its clone receives the same cues until the moment of separation in time period t after which it begins to receive reciprocal, opposite cues, which lasts until the end of ontogeny. The vertical axis within each panel depicts the phenotypic distance between focal individuals and their clones. The horizontal axis depicts the time period in which pairs of twins were separated. The phenotypic distance at the end of ontogeny between a focal individual and its clone corresponds to the Euclidean distance between their phenotypes. Grey lines and diamonds depict 'absolute' phenotypic distance, the average distance between the 10,000 focal individuals and their clones at the end of ontogeny (ranging from 0 to $20\sqrt{2}$, scaled to a 0 to 1 range). Black lines and circles depict 'proportional' distance, the average absolute distance divided by the maximum possible distance following separation.

Diminishing rewards & diminishing penalties

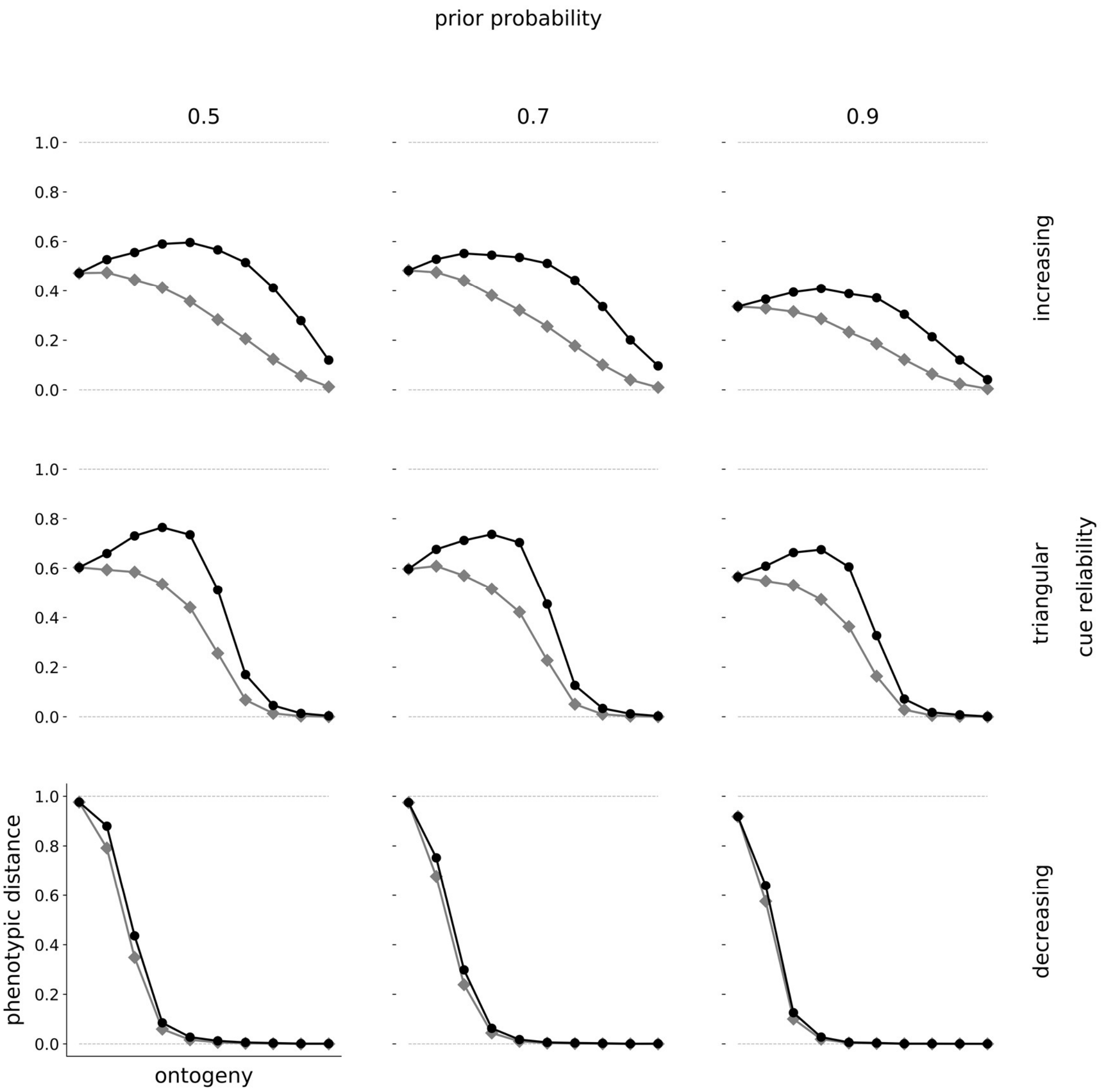

Figure A5.9. Plasticity across ontogeny. The fitness rewards for correct specializations are **diminishing** and fitness penalties for incorrect specializations are **diminishing** across all panels. The prior probability of E_1 varies across columns and the cue reliability pattern varies across rows. Each panel represents T experimental 'twin studies', one for each $t \in \{1, T\}$. Outcomes of each twin study are marked by a grey diamond and a black circle. For each study we simulate 10,000 pairs of identical twins who follow the optimal policy and track their development across ontogeny. The environmental state is fixed to E_1. For each pair of twins, one individual (the 'focal') receives a set of environmental cues across ontogeny simulated from the prior probability and cue reliability pattern. Its clone receives the same cues until the moment of separation in time period t after which it begins to receive reciprocal, opposite cues, which lasts until the end of ontogeny. The vertical axis within each panel depicts the phenotypic distance between focal individuals and their clones. The horizontal axis depicts the time period in which pairs of twins were separated. The phenotypic distance at the end of ontogeny between a focal individual and its clone corresponds to the Euclidean distance between their phenotypes. Grey lines and diamonds depict 'absolute' phenotypic distance, the average distance between the 10,000 focal individuals and their clones at the end of ontogeny (ranging from 0 to $20\sqrt{2}$, scaled to a 0 to 1 range). Black lines and circles depict 'proportional' distance, the average absolute distance divided by the maximum possible distance following separation.

b) Optimal developmental policies

Linear rewards & linear penalties

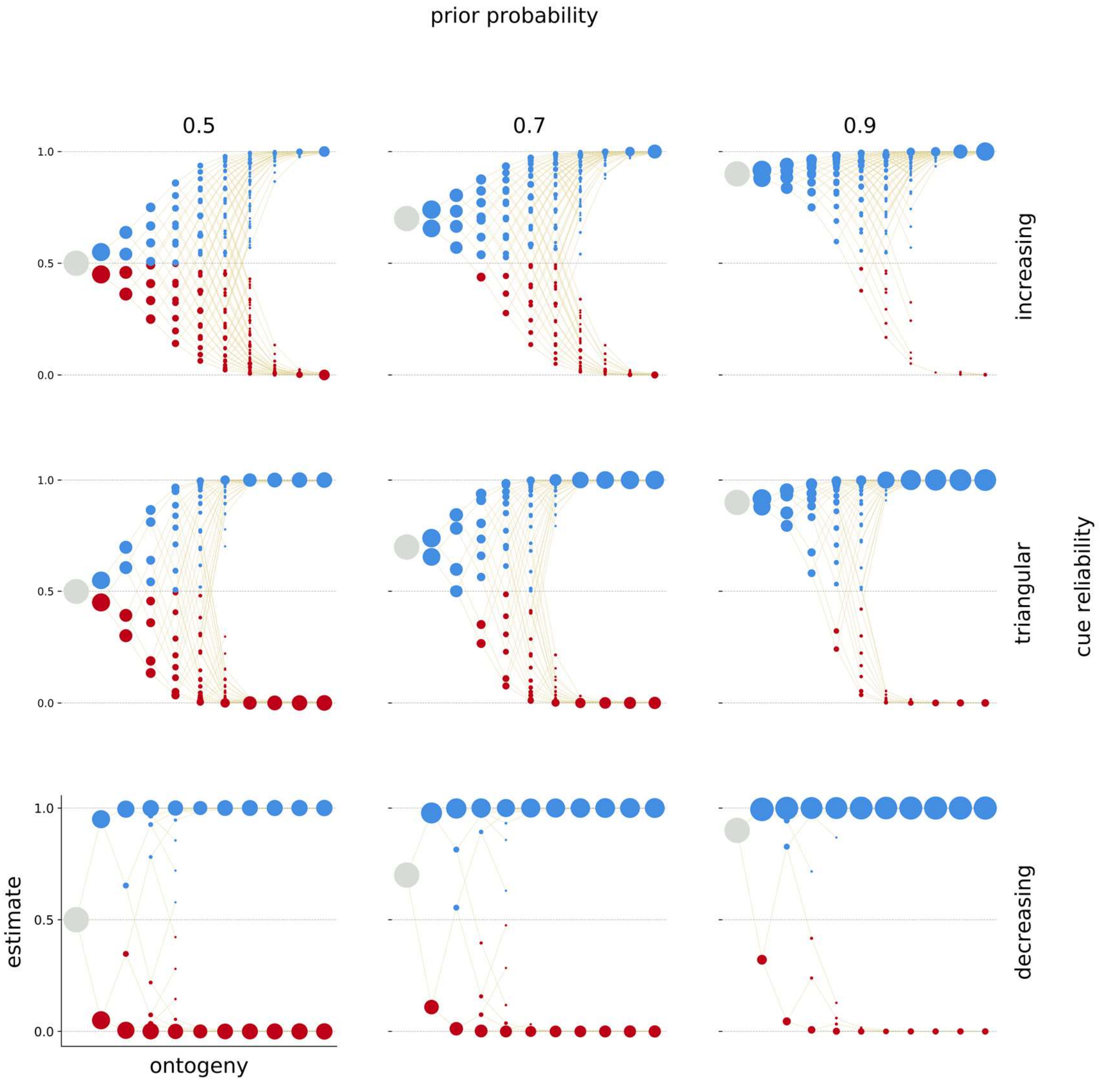

Figure A5.10. Optimal developmental policies. The fitness rewards for correct specializations are **linear** and fitness penalties for incorrect specializations are **linear** across all panels. The prior probability of E_1 varies across columns and the cue reliability pattern varies across rows. Each panel depicts the optimal developmental policy for the corresponding parameter values as well as information about the probability of reaching each possible state. The horizontal axis shows developmental time and the vertical axis shows an organism's estimate of being in E_1. Each organism begins ontogeny with the same prior (large grey circle) and then, in each time period, samples a cue, updates its posterior, and makes a phenotypic decision. Beige lines represent possible changes in posteriors across development, tracking possible developmental trajectories. Colored circles represent phenotypic decisions: black indicates waiting, red specializing towards P_0, and blue specializing towards P_1. The area of a circle is proportional to the probability of reaching the corresponding state. These probabilities sum to one within a time period. We only show states that have a probability of more than 0.5% of being reached.

Linear reward & increasing penalties

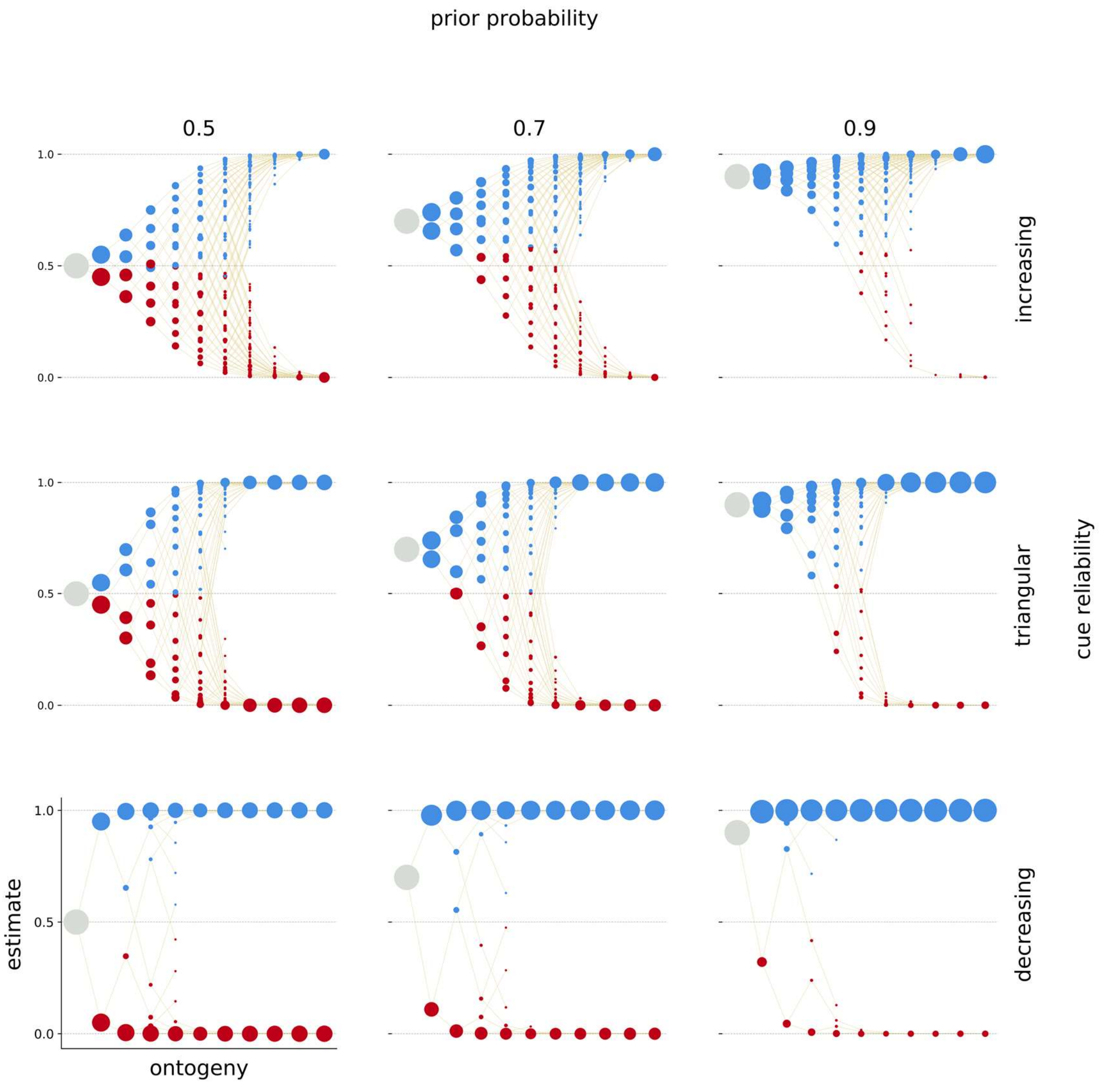

Figure A5.11. Optimal developmental policies. The fitness rewards for correct specializations are **linear** and fitness penalties for incorrect specializations are **increasing** across all panels. The prior probability of E_1 varies across columns and the cue reliability pattern varies across rows. Each panel depicts the optimal developmental policy for the corresponding parameter values as well as information about the probability of reaching each possible state. The horizontal axis shows developmental time and the vertical axis shows an organism's estimate of being in E_1. Each organism begins ontogeny with the same prior (large grey circle) and then, in each time period, samples a cue, updates its posterior, and makes a phenotypic decision. Beige lines represent possible changes in posteriors across development, tracking possible developmental trajectories. Colored circles represent phenotypic decisions: black indicates waiting, red specializing towards P_0, and blue specializing towards P_1. The area of a circle is proportional to the probability of reaching the corresponding state. These probabilities sum to one within a time period. We only show states that have a probability of more than 0.5% of being reached.

Linear rewards & diminishing penalties

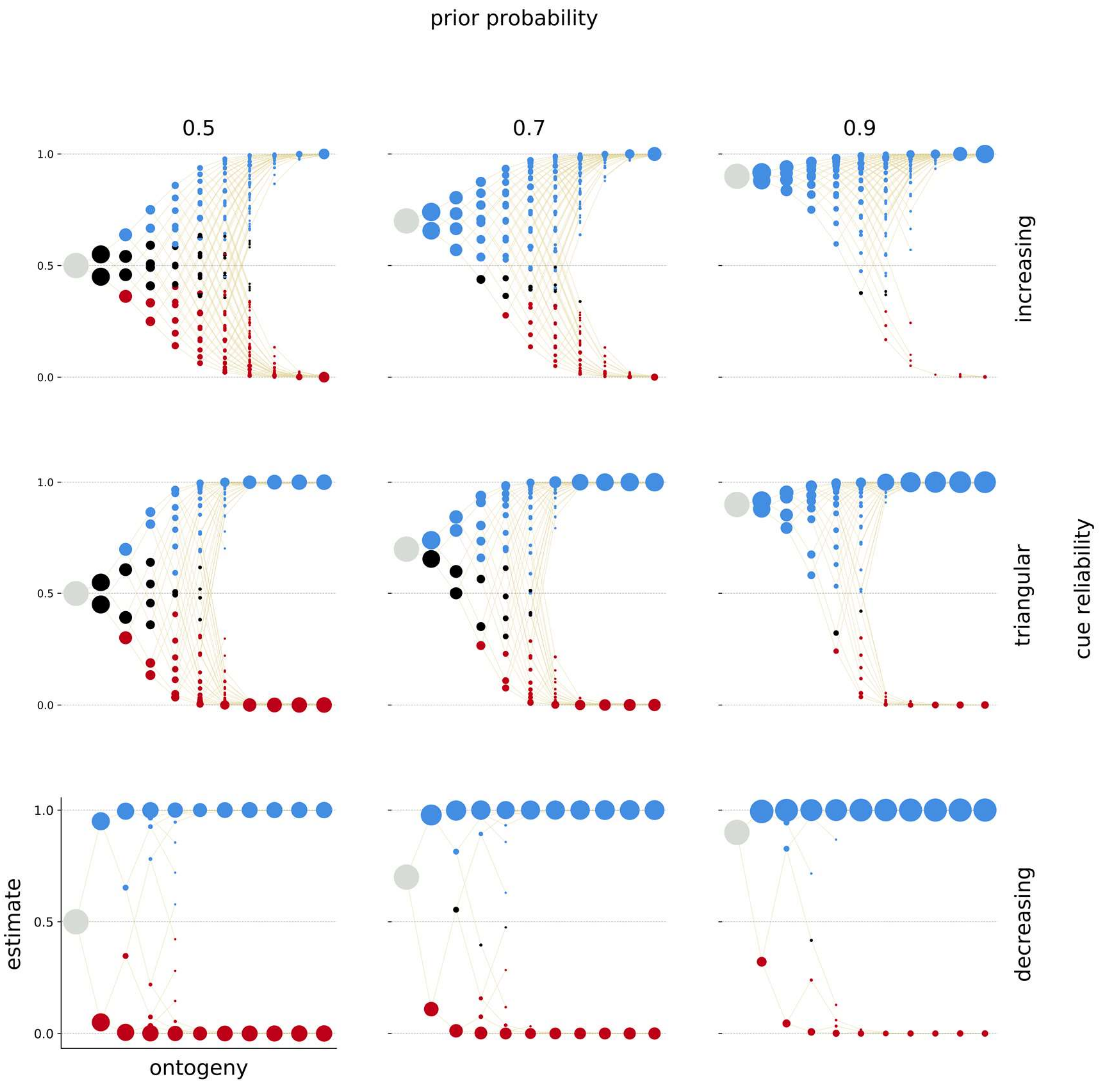

Figure A5.12. Optimal developmental policies. The fitness rewards for correct specializations are **linear** and fitness penalties for incorrect specializations are **diminishing** across all panels. The prior probability of E_1 varies across columns and the cue reliability pattern varies across rows. Each panel depicts the optimal developmental policy for the corresponding parameter values as well as information about the probability of reaching each possible state. The horizontal axis shows developmental time and the vertical axis shows an organism's estimate of being in E_1. Each organism begins ontogeny with the same prior (large grey circle) and then, in each time period, samples a cue, updates its posterior, and makes a phenotypic decision. Beige lines represent possible changes in posteriors across development, tracking possible developmental trajectories. Colored circles represent phenotypic decisions: black indicates waiting, red specializing towards P_0, and blue specializing towards P_1. The area of a circle is proportional to the probability of reaching the corresponding state. These probabilities sum to one within a time period. We only show states that have a probability of more than 0.5% of being reached.

Increasing rewards & linear penalties

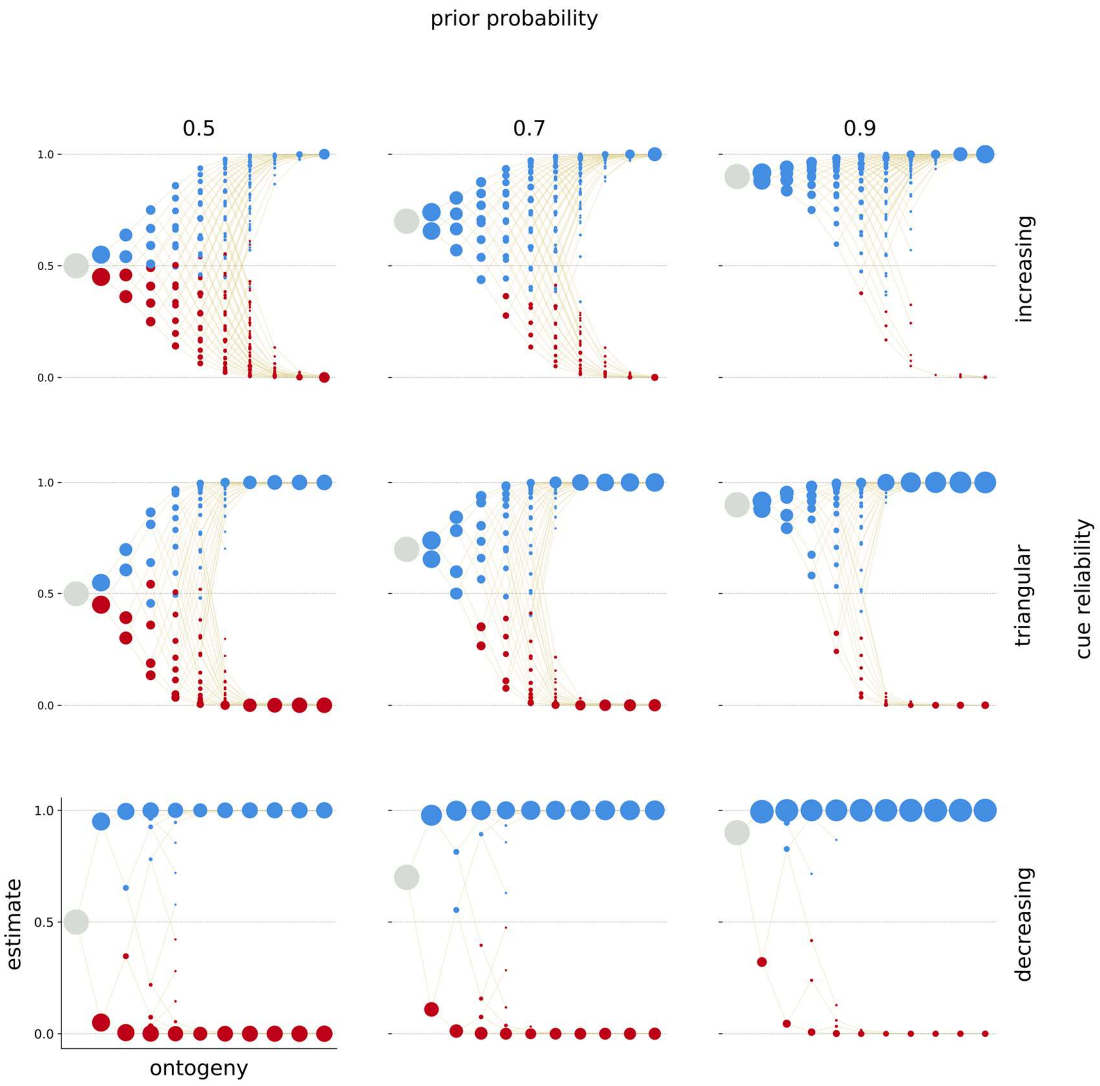

Figure A5.13. Optimal developmental policies. The fitness rewards for correct specializations are **increasing** and fitness penalties for incorrect specializations are **linear** across all panels. The prior probability of E_1 varies across columns and the cue reliability pattern varies across rows. Each panel depicts the optimal developmental policy for the corresponding parameter values as well as information about the probability of reaching each possible state. The horizontal axis shows developmental time and the vertical axis shows an organism's estimate of being in E_1. Each organism begins ontogeny with the same prior (large grey circle) and then, in each time period, samples a cue, updates its posterior, and makes a phenotypic decision. Beige lines represent possible changes in posteriors across development, tracking possible developmental trajectories. Colored circles represent phenotypic decisions: black indicates waiting, red specializing towards P_0, and blue specializing towards P_1. The area of a circle is proportional to the probability of reaching the corresponding state. These probabilities sum to one within a time period. We only show states that have a probability of more than 0.5% of being reached.

Increasing reward & increasing penalties

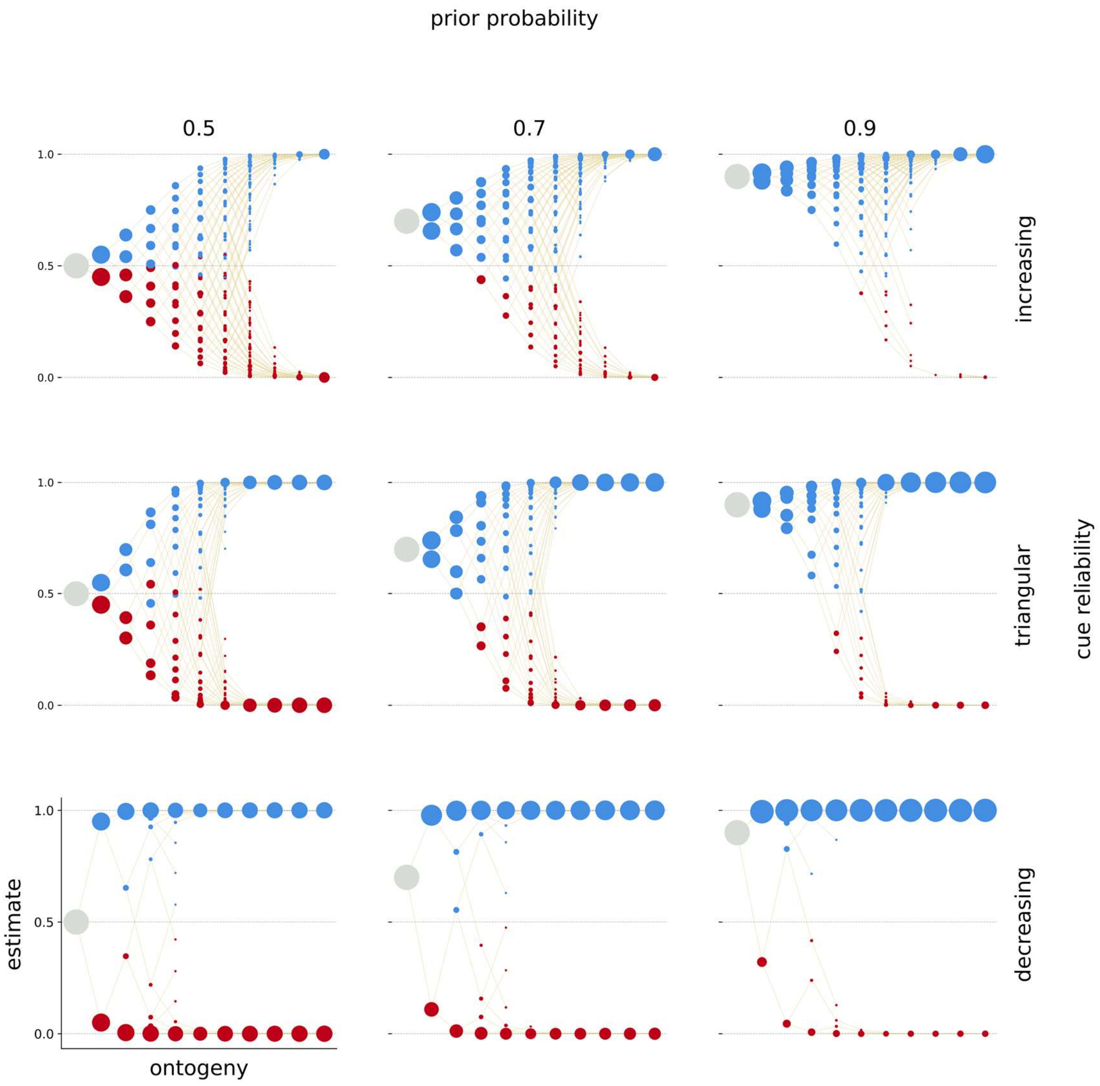

Figure A5.14. Optimal developmental policies. The fitness rewards for correct specializations are **increasing** and fitness penalties for incorrect specializations are **increasing** across all panels. The prior probability of E_1 varies across columns and the cue reliability pattern varies across rows. Each panel depicts the optimal developmental policy for the corresponding parameter values as well as information about the probability of reaching each possible state. The horizontal axis shows developmental time and the vertical axis shows an organism's estimate of being in E_1. Each organism begins ontogeny with the same prior (large grey circle) and then, in each time period, samples a cue, updates its posterior, and makes a phenotypic decision. Beige lines represent possible changes in posteriors across development, tracking possible developmental trajectories. Colored circles represent phenotypic decisions: black indicates waiting, red specializing towards P_0, and blue specializing towards P_1. The area of a circle is proportional to the probability of reaching the corresponding state. These probabilities sum to one within a time period. We only show states that have a probability of more than 0.5% of being reached.

Increasing rewards & diminishing penalties

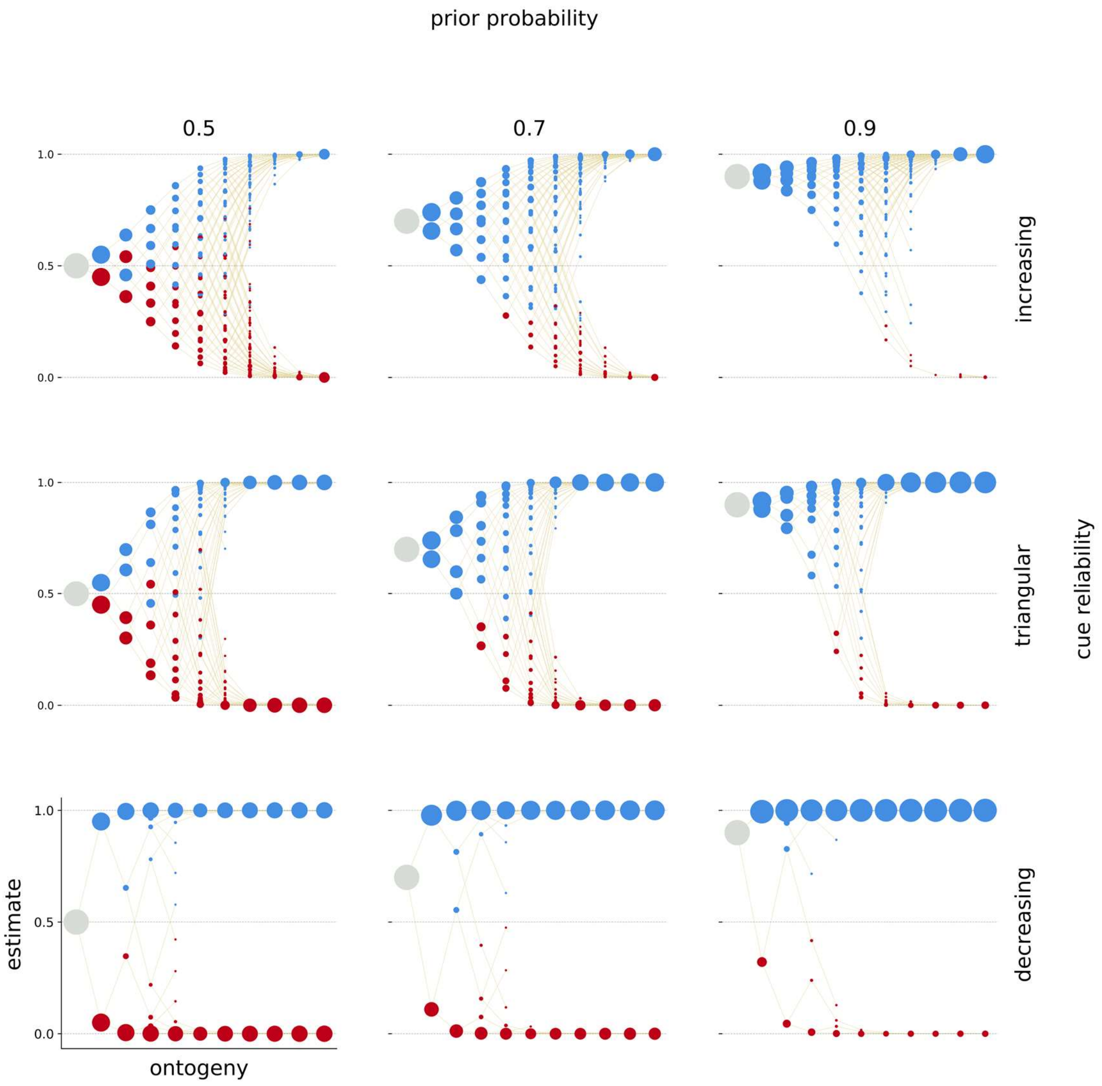

Figure A5.15. Optimal developmental policies. The fitness rewards for correct specializations are **increasing** and fitness penalties for incorrect specializations are **diminishing** across all panels. The prior probability of E_1 varies across columns and the cue reliability pattern varies across rows. Each panel depicts the optimal developmental policy for the corresponding parameter values as well as information about the probability of reaching each possible state. The horizontal axis shows developmental time and the vertical axis shows an organism's estimate of being in E_1. Each organism begins ontogeny with the same prior (large grey circle) and then, in each time period, samples a cue, updates its posterior, and makes a phenotypic decision. Beige lines represent possible changes in posteriors across development, tracking possible developmental trajectories. Colored circles represent phenotypic decisions: black indicates waiting, red specializing towards P_0, and blue specializing towards P_1. The area of a circle is proportional to the probability of reaching the corresponding state. These probabilities sum to one within a time period. We only show states that have a probability of more than 0.5% of being reached.

Diminishing rewards & linear penalties

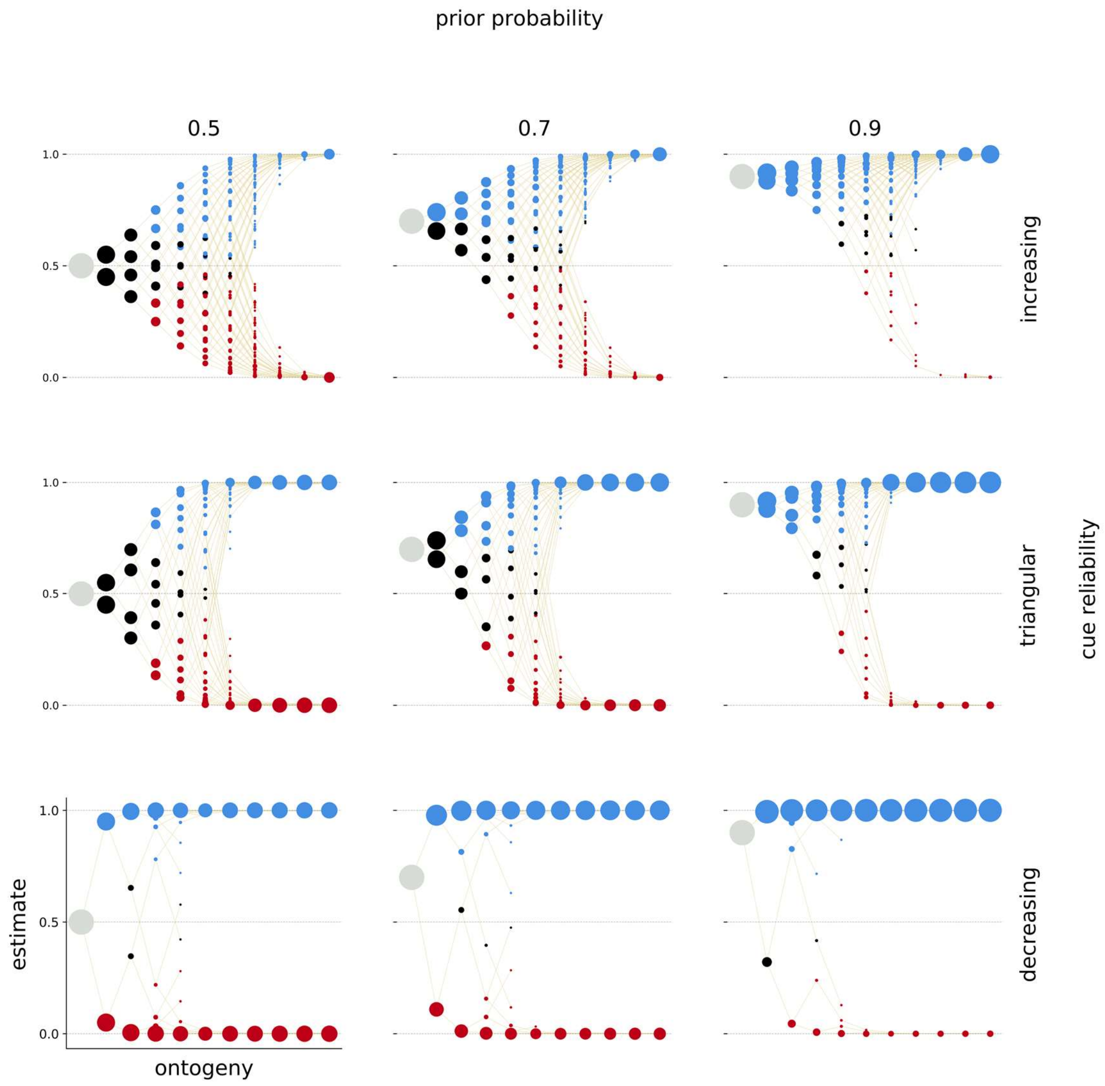

Figure A5.16. Optimal developmental policies. The fitness rewards for correct specializations are **diminishing** and fitness penalties for incorrect specializations are **linear** across all panels. The prior probability of E_1 varies across columns and the cue reliability pattern varies across rows. Each panel depicts the optimal developmental policy for the corresponding parameter values as well as information about the probability of reaching each possible state. The horizontal axis shows developmental time and the vertical axis shows an organism's estimate of being in E_1. Each organism begins ontogeny with the same prior (large grey circle) and then, in each time period, samples a cue, updates its posterior, and makes a phenotypic decision. Beige lines represent possible changes in posteriors across development, tracking possible developmental trajectories. Colored circles represent phenotypic decisions: black indicates waiting, red specializing towards P_0, and blue specializing towards P_1. The area of a circle is proportional to the probability of reaching the corresponding state. These probabilities sum to one within a time period. We only show states that have a probability of more than 0.5% of being reached.

Diminishing reward & increasing penalties

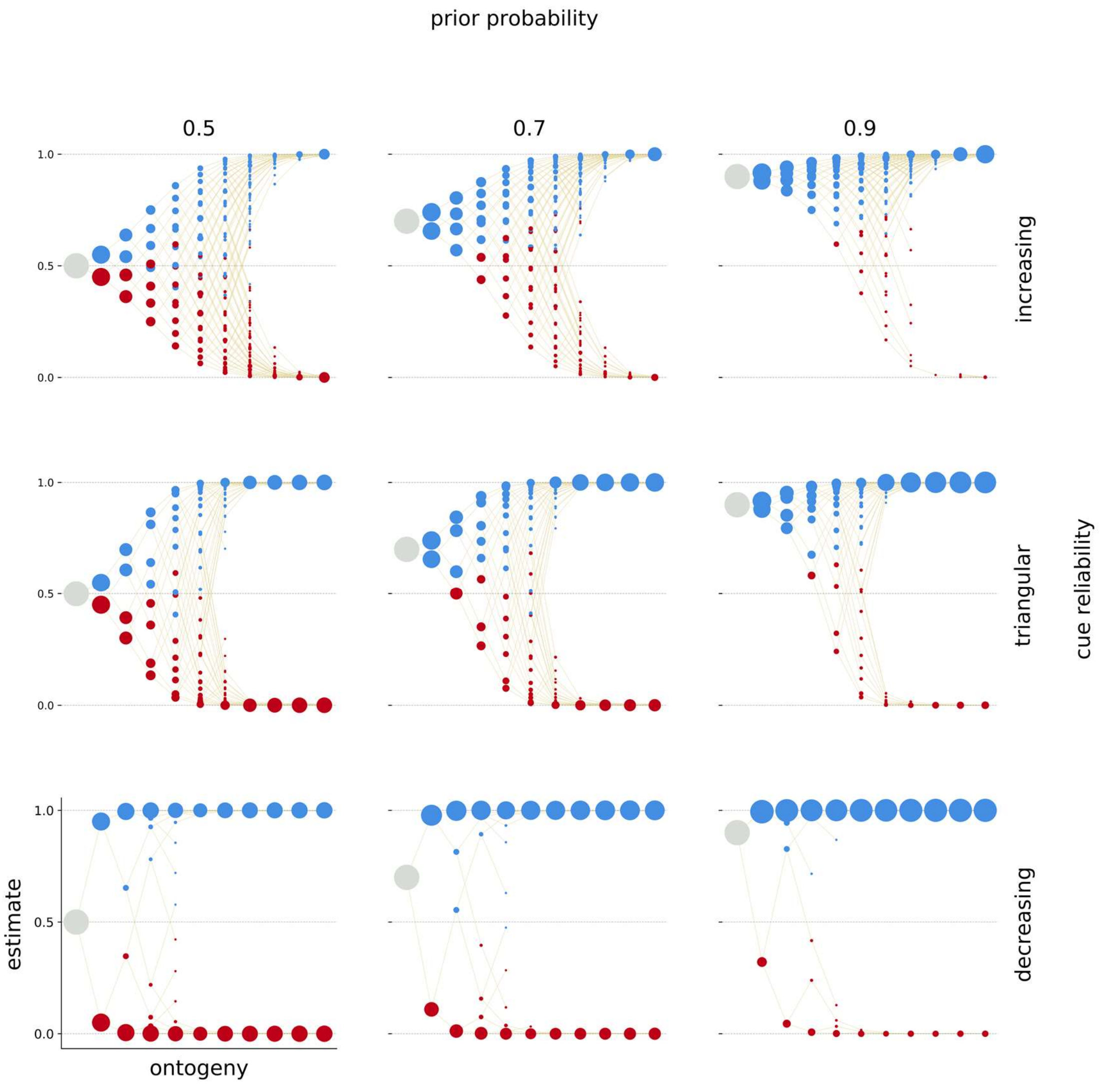

Figure A5.17. Optimal developmental policies. The fitness rewards for correct specializations are **diminishing** and fitness penalties for incorrect specializations are **increasing** across all panels. The prior probability of E_1 varies across columns and the cue reliability pattern varies across rows. Each panel depicts the optimal developmental policy for the corresponding parameter values as well as information about the probability of reaching each possible state. The horizontal axis shows developmental time and the vertical axis shows an organism's estimate of being in E_1. Each organism begins ontogeny with the same prior (large grey circle) and then, in each time period, samples a cue, updates its posterior, and makes a phenotypic decision. Beige lines represent possible changes in posteriors across development, tracking possible developmental trajectories. Colored circles represent phenotypic decisions: black indicates waiting, red specializing towards P_0, and blue specializing towards P_1. The area of a circle is proportional to the probability of reaching the corresponding state. These probabilities sum to one within a time period. We only show states that have a probability of more than 0.5% of being reached.

Diminishing rewards & diminishing penalties

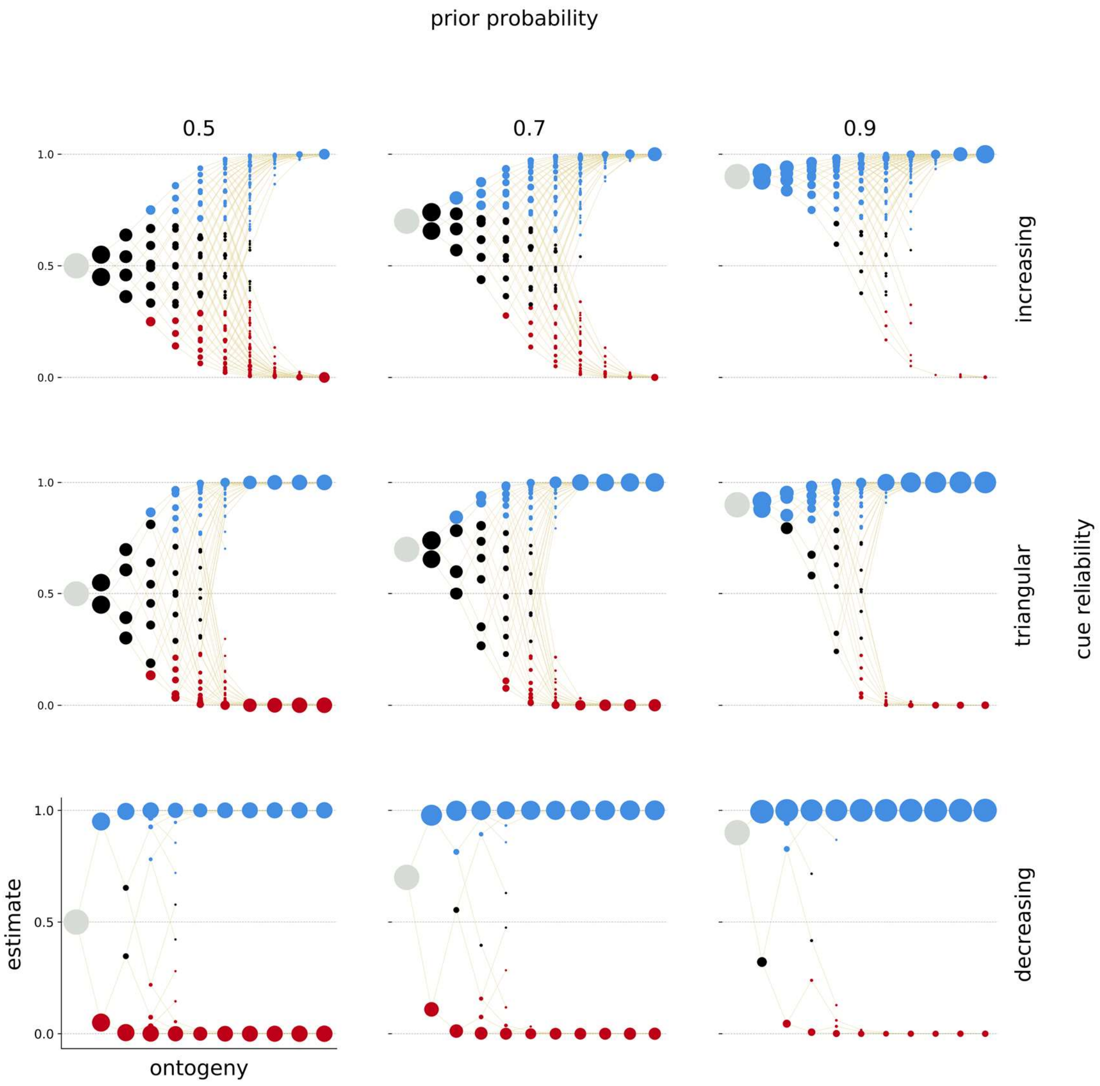

Figure A5.18. Optimal developmental policies. The fitness rewards for correct specializations are **diminishing** and fitness penalties for incorrect specializations are **diminishing** across all panels. The prior probability of E_1 varies across columns and the cue reliability pattern varies across rows. Each panel depicts the optimal developmental policy for the corresponding parameter values as well as information about the probability of reaching each possible state. The horizontal axis shows developmental time and the vertical axis shows an organism's estimate of being in E_1. Each organism begins ontogeny with the same prior (large grey circle) and then, in each time period, samples a cue, updates its posterior, and makes a phenotypic decision. Beige lines represent possible changes in posteriors across development, tracking possible developmental trajectories. Colored circles represent phenotypic decisions: black indicates waiting, red specializing towards P_0, and blue specializing towards P_1. The area of a circle is proportional to the probability of reaching the corresponding state. These probabilities sum to one within a time period. We only show states that have a probability of more than 0.5% of being reached.

c) Rank-order stability

Linear rewards & linear penalties

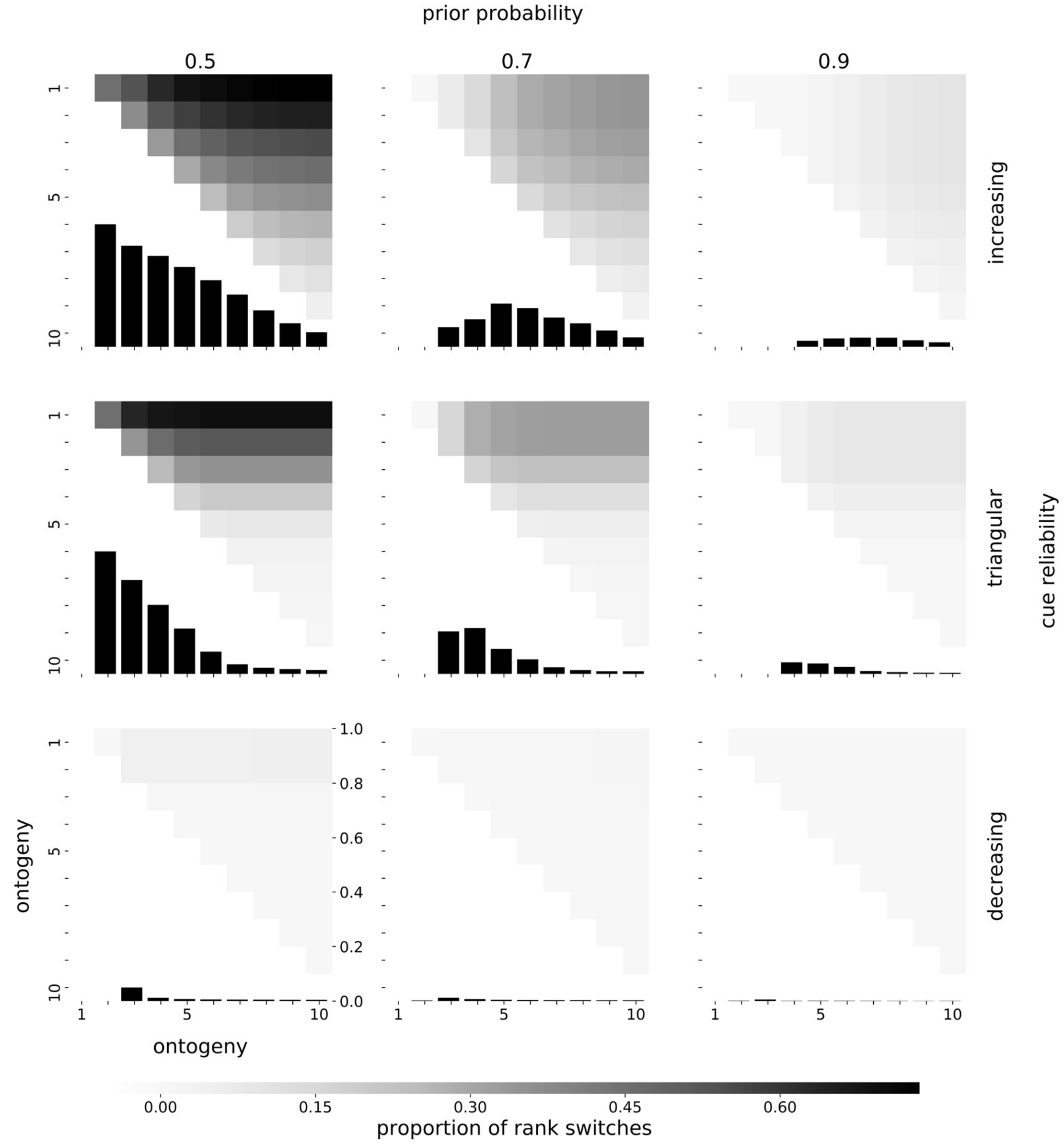

Figure A5.19. Rank-order stability. The fitness rewards for correct specializations are **linear** and fitness penalties for incorrect specializations are **linear** across all panels. The prior probability of E_1 varies across columns and the cue reliability pattern varies across rows. Each panel depicts a simulation of 10,000 organisms following the optimal policy across ontogeny. The environmental state is fixed to E_1. In each time period, organisms are ranked according to the number of specializations towards P_1. Organisms with the same number of specializations share a rank. Each square panel depicts two sets of results, one in the upper right triangle and another in the lower left triangle. For the upper right triangle, the relevant axes are the horizontal and the left vertical, each depicting the full range of ontogenetic time periods. Each cell in this triangle indicates the proportion of rank-switches occurring from the time period on the horizontal axis to the time period on the (left) vertical axis in gray scale, with lighter cells indicating fewer rank-switches and darker cells more rank-switches. The lower left triangle within each panel zooms in on the diagonal of the upper right triangle, depicting the proportion of rank-switches between consecutive time periods in a bar chart. We highlight this scenario as it is the most relevant for empirical research on animal personality where repeatability is typically measured across consecutive years. For this portion of the panel, the horizontal axis depicts ontogenetic time periods and the right vertical axis depicts the proportion of rank-switches in that time period.

Linear reward & increasing penalties

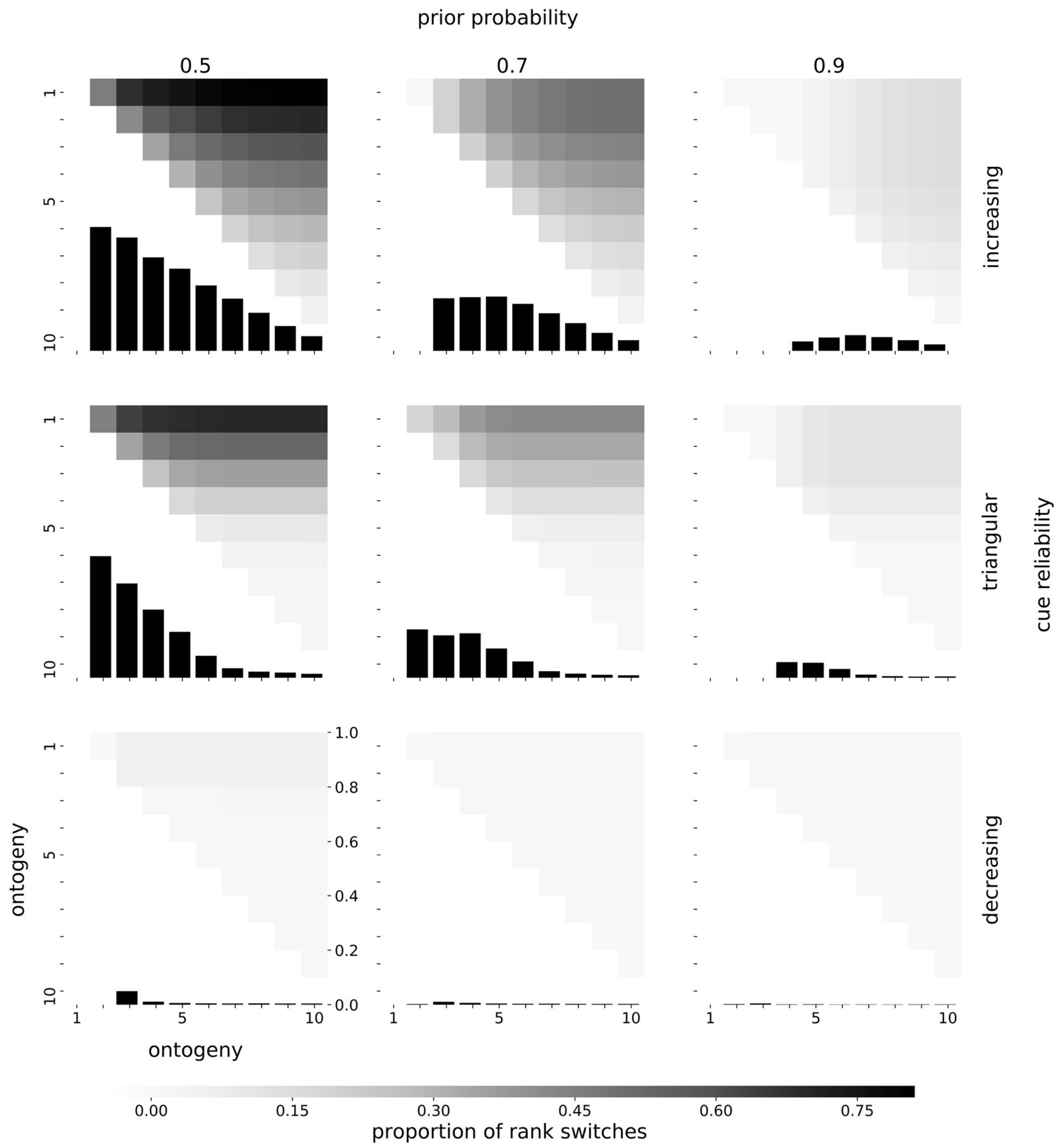

Figure A5.20. Rank-order stability. The fitness rewards for correct specializations are **linear** and fitness penalties for incorrect specializations are **increasing** across all panels. The prior probability of E_1 varies across columns and the cue reliability pattern varies across rows. Each panel depicts a simulation of 10,000 organisms following the optimal policy across ontogeny. The environmental state is fixed to E_1. In each time period, organisms are ranked according to the number of specializations towards P_1. Organisms with the same number of specializations share a rank. Each square panel depicts two sets of results, one in the upper right triangle and another in the lower left triangle. For the upper right triangle, the relevant axes are the horizontal and the left vertical, each depicting the full range of ontogenetic time periods. Each cell in this triangle indicates the proportion of rank-switches occurring from the time period on the horizontal axis to the time period on the (left) vertical axis in gray scale, with lighter cells indicating fewer rank-switches and darker cells more rank-switches. The lower left triangle within each panel zooms in on the diagonal of the upper right triangle, depicting the proportion of rank-switches between consecutive time periods in a bar chart. We highlight this scenario as it is the most relevant for empirical research on animal personality where repeatability is typically measured across consecutive years. For this portion of the panel, the horizontal axis depicts ontogenetic time periods and the right vertical axis depicts the proportion of rank-switches in that time period.

Linear rewards & diminishing penalties

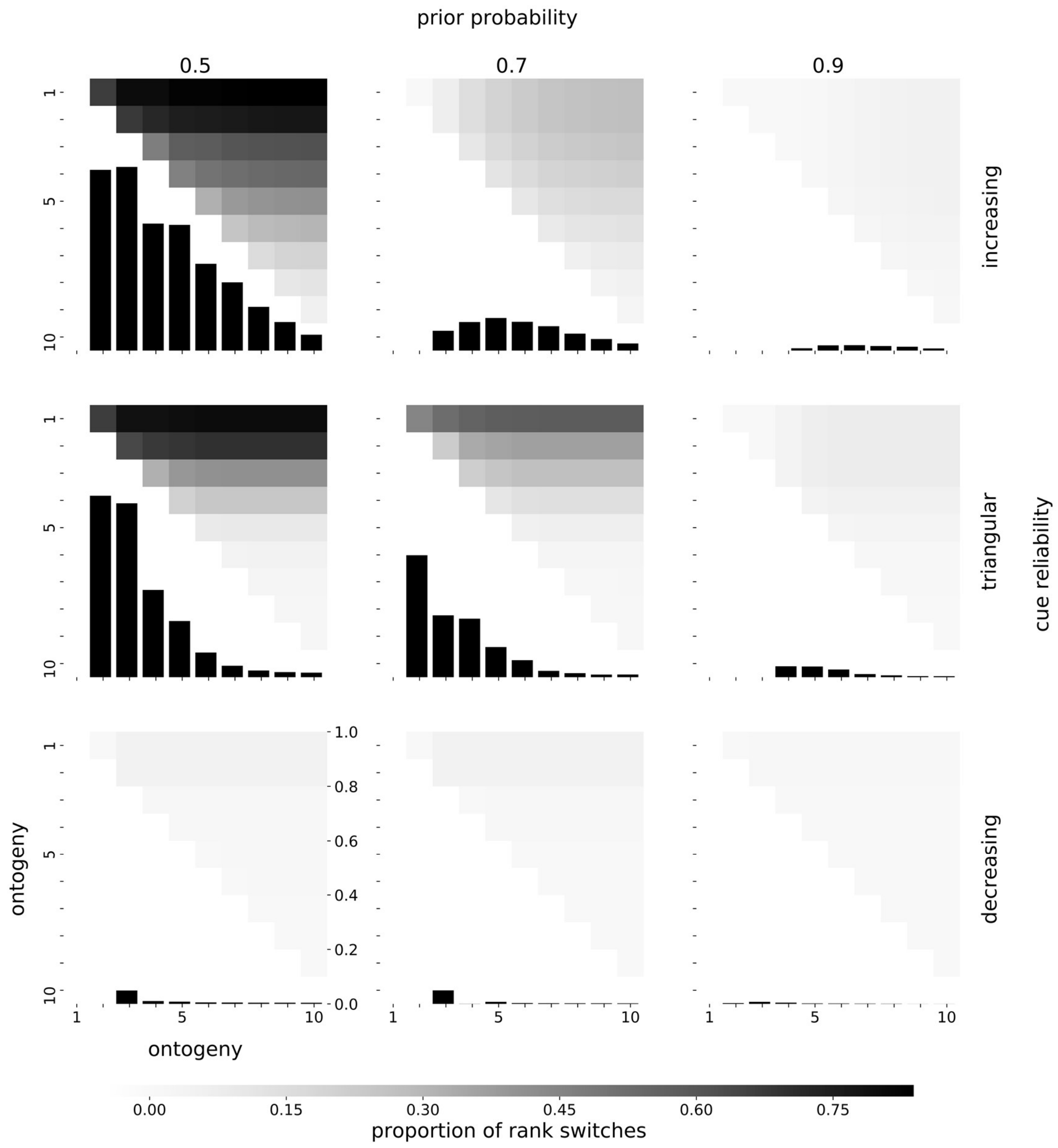

Figure A5.21. Rank-order stability. The fitness rewards for correct specializations are **linear** and fitness penalties for incorrect specializations are **diminishing** across all panels. The prior probability of E_1 varies across columns and the cue reliability pattern varies across rows. Each panel depicts a simulation of 10,000 organisms following the optimal policy across ontogeny. The environmental state is fixed to E_1. In each time period, organisms are ranked according to the number of specializations towards P_1. Organisms with the same number of specializations share a rank. Each square panel depicts two sets of results, one in the upper right triangle and another in the lower left triangle. For the upper right triangle, the relevant axes are the horizontal and the left vertical, each depicting the full range of ontogenetic time periods. Each cell in this triangle indicates the proportion of rank-switches occurring from the time period on the horizontal axis to the time period on the (left) vertical axis in gray scale, with lighter cells indicating fewer rank-switches and darker cells more rank-switches. The lower left triangle within each panel zooms in on the diagonal of the upper right triangle, depicting the proportion of rank-switches between consecutive time periods in a bar chart. We highlight this scenario as it is the most relevant for empirical research on animal personality where repeatability is typically measured across consecutive years. For this portion of the panel, the horizontal axis depicts ontogenetic time periods and the right vertical axis depicts the proportion of rank-switches in that time period.

Increasing rewards & linear penalties

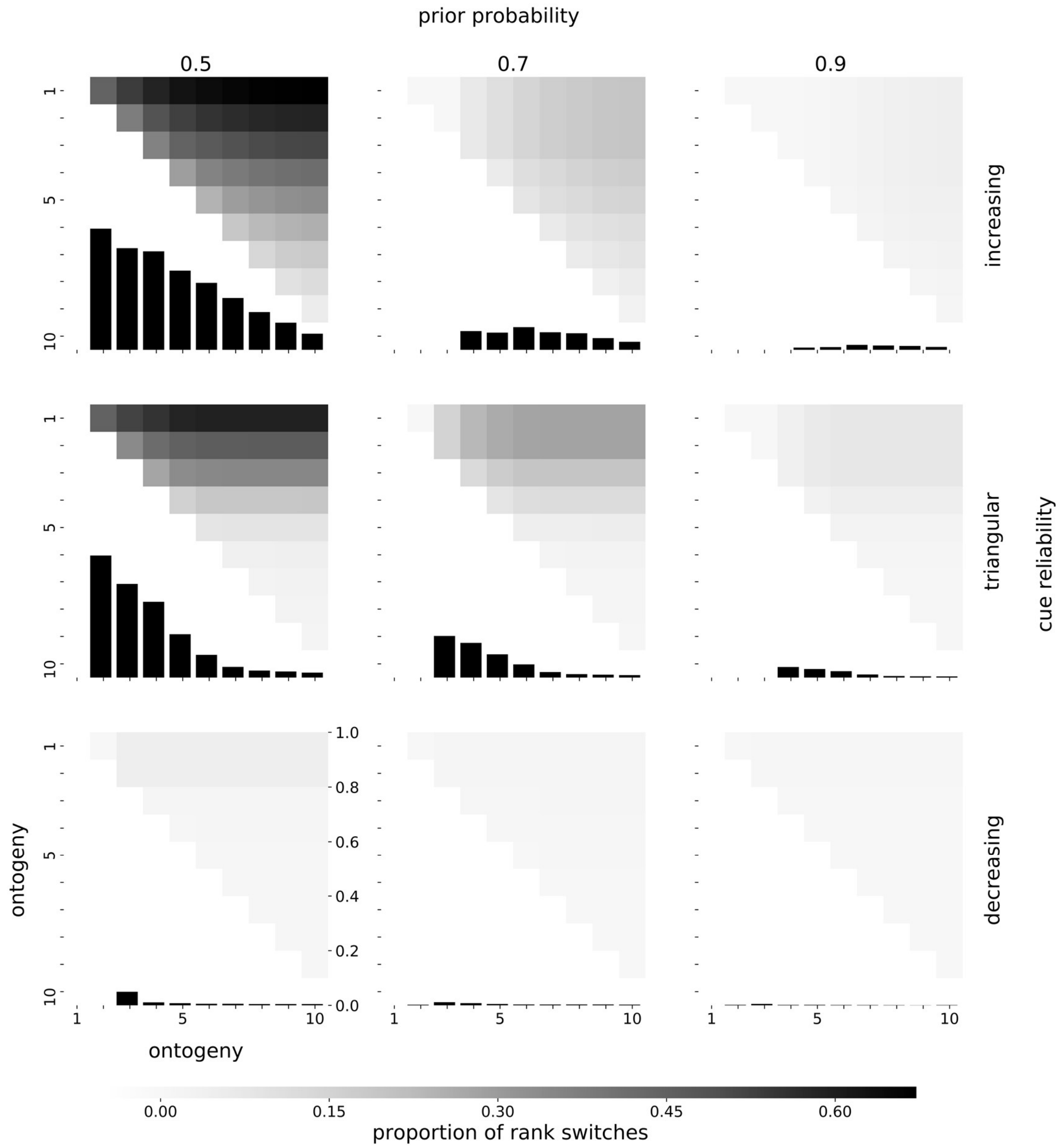

Figure A5.22. Rank-order stability. The fitness rewards for correct specializations are **increasing** and fitness penalties for incorrect specializations are **linear** across all panels. The prior probability of E_1 varies across columns and the cue reliability pattern varies across rows. Each panel depicts a simulation of 10,000 organisms following the optimal policy across ontogeny. The environmental state is fixed to E_1. In each time period, organisms are ranked according to the number of specializations towards P_1. Organisms with the same number of specializations share a rank. Each square panel depicts two sets of results, one in the upper right triangle and another in the lower left triangle. For the upper right triangle, the relevant axes are the horizontal and the left vertical, each depicting the full range of ontogenetic time periods. Each cell in this triangle indicates the proportion of rank-switches occurring from the time period on the horizontal axis to the time period on the (left) vertical axis in gray scale, with lighter cells indicating fewer rank-switches and darker cells more rank-switches. The lower left triangle within each panel zooms in on the diagonal of the upper right triangle, depicting the proportion of rank-switches between consecutive time periods in a bar chart. We highlight this scenario as it is the most relevant for empirical research on animal personality where repeatability is typically measured across consecutive years. For this portion of the panel, the horizontal axis depicts ontogenetic time periods and the right vertical axis depicts the proportion of rank-switches in that time period.

Increasing reward & increasing penalties

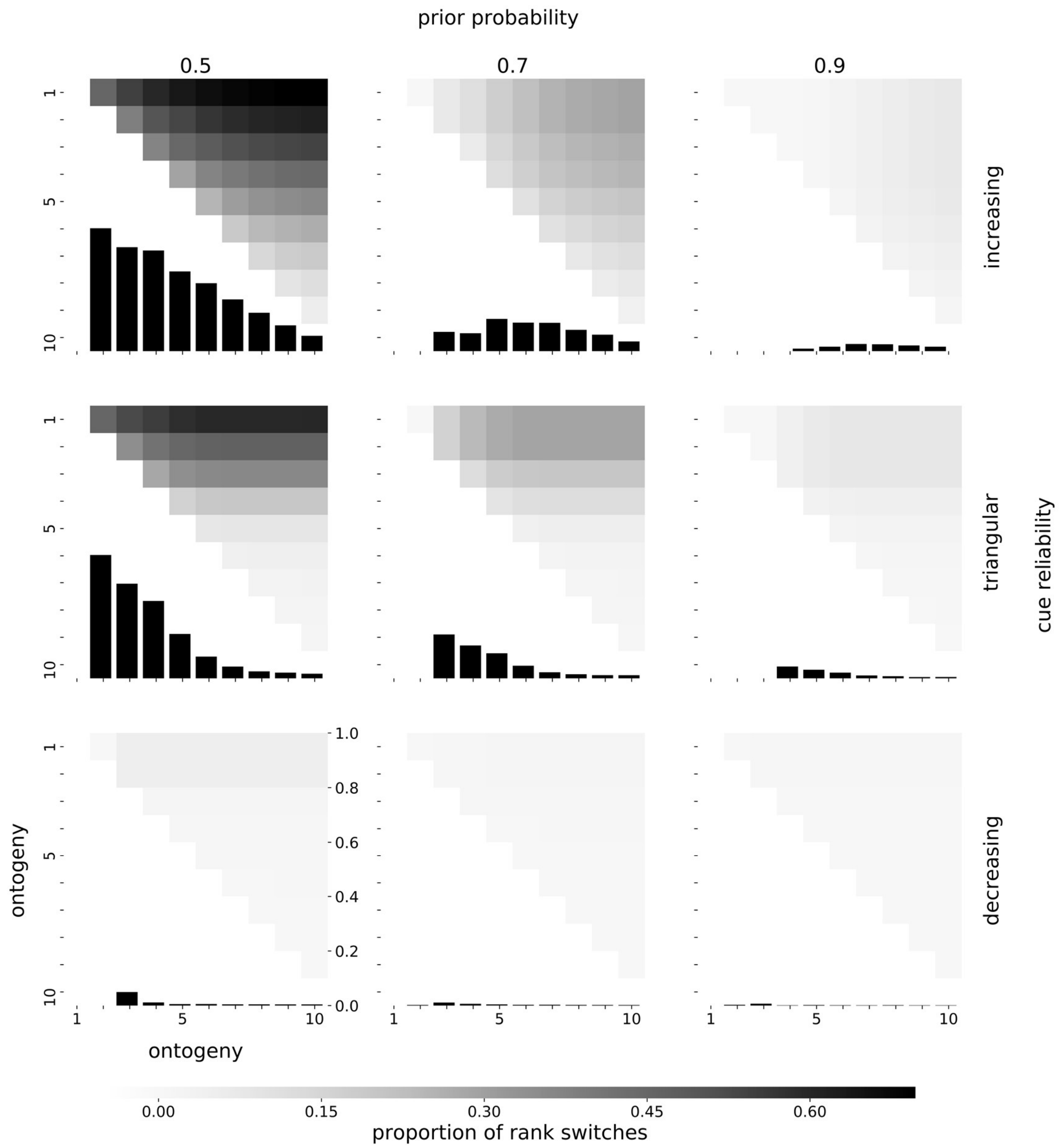

Figure A5.23. Rank-order stability. The fitness rewards for correct specializations are **increasing** and fitness penalties for incorrect specializations are **increasing** across all panels. The prior probability of E_1 varies across columns and the cue reliability pattern varies across rows. Each panel depicts a simulation of 10,000 organisms following the optimal policy across ontogeny. The environmental state is fixed to E_1. In each time period, organisms are ranked according to the number of specializations towards P_1. Organisms with the same number of specializations share a rank. Each square panel depicts two sets of results, one in the upper right triangle and another in the lower left triangle. For the upper right triangle, the relevant axes are the horizontal and the left vertical, each depicting the full range of ontogenetic time periods. Each cell in this triangle indicates the proportion of rank-switches occurring from the time period on the horizontal axis to the time period on the (left) vertical axis in gray scale, with lighter cells indicating fewer rank-switches and darker cells more rank-switches. The lower left triangle within each panel zooms in on the diagonal of the upper right triangle, depicting the proportion of rank-switches between consecutive time periods in a bar chart. We highlight this scenario as it is the most relevant for empirical research on animal personality where repeatability is typically measured across consecutive years. For this portion of the panel, the horizontal axis depicts ontogenetic time periods and the right vertical axis depicts the proportion of rank-switches in that time period.

Increasing rewards & diminishing penalties

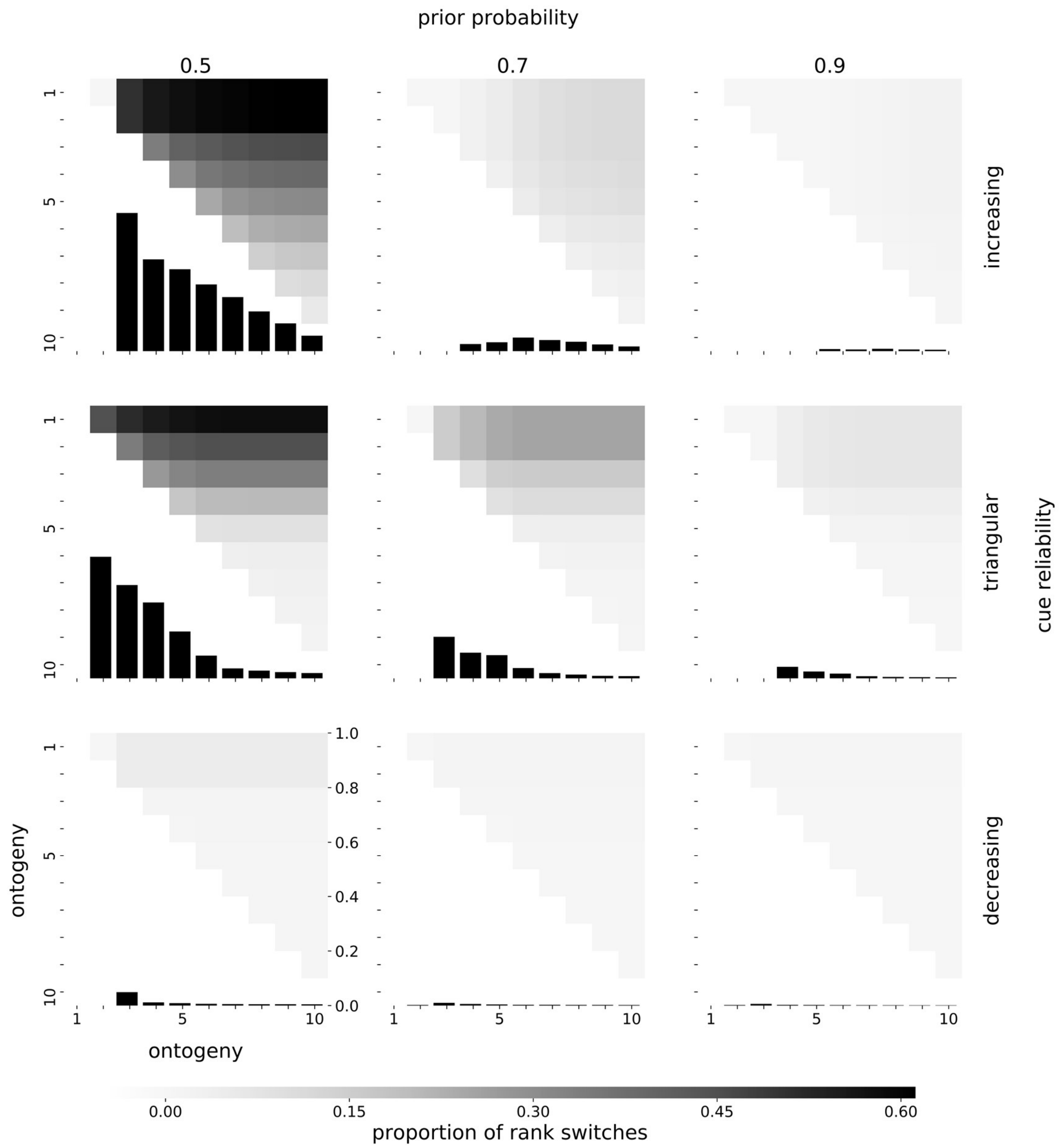

Figure A5.24. Rank-order stability. The fitness rewards for correct specializations are **increasing** and fitness penalties for incorrect specializations are **diminishing** across all panels. The prior probability of E_1 varies across columns and the cue reliability pattern varies across rows. Each panel depicts a simulation of 10,000 organisms following the optimal policy across ontogeny. The environmental state is fixed to E_1. In each time period, organisms are ranked according to the number of specializations towards P_1. Organisms with the same number of specializations share a rank. Each square panel depicts two sets of results, one in the upper right triangle and another in the lower left triangle. For the upper right triangle, the relevant axes are the horizontal and the left vertical, each depicting the full range of ontogenetic time periods. Each cell in this triangle indicates the proportion of rank-switches occurring from the time period on the horizontal axis to the time period on the (left) vertical axis in gray scale, with lighter cells indicating fewer rank-switches and darker cells more rank-switches. The lower left triangle within each panel zooms in on the diagonal of the upper right triangle, depicting the proportion of rank-switches between consecutive time periods in a bar chart. We highlight this scenario as it is the most relevant for empirical research on animal personality where repeatability is typically measured across consecutive years. For this portion of the panel, the horizontal axis depicts ontogenetic time periods and the right vertical axis depicts the proportion of rank-switches in that time period.

Diminishing rewards & linear penalties

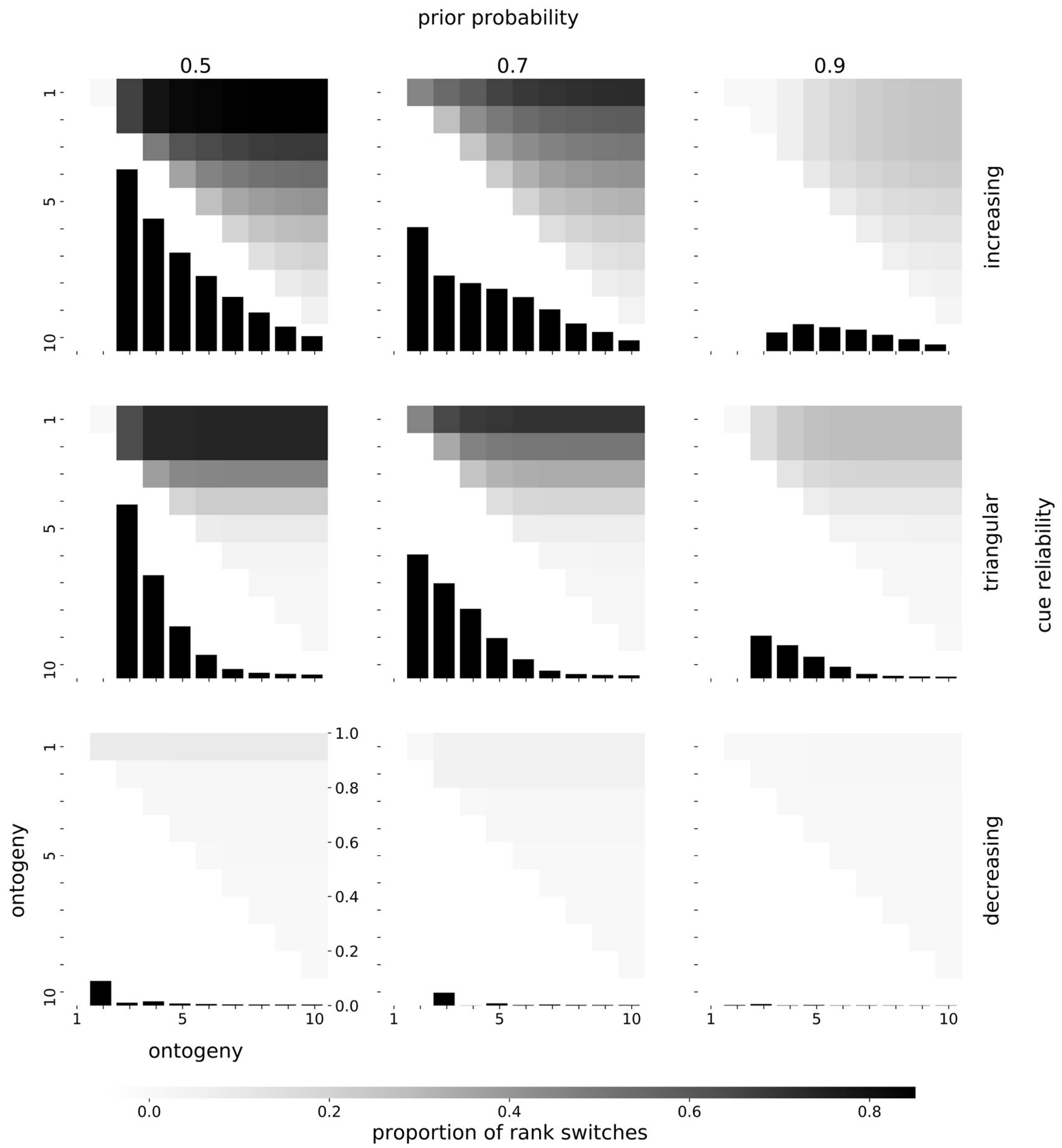

Figure A5.25. Rank-order stability. The fitness rewards for correct specializations are **diminishing** and fitness penalties for incorrect specializations are **linear** across all panels. The prior probability of E_1 varies across columns and the cue reliability pattern varies across rows. Each panel depicts a simulation of 10,000 organisms following the optimal policy across ontogeny. The environmental state is fixed to E_1. In each time period, organisms are ranked according to the number of specializations towards P_1. Organisms with the same number of specializations share a rank. Each square panel depicts two sets of results, one in the upper right triangle and another in the lower left triangle. For the upper right triangle, the relevant axes are the horizontal and the left vertical, each depicting the full range of ontogenetic time periods. Each cell in this triangle indicates the proportion of rank-switches occurring from the time period on the horizontal axis to the time period on the (left) vertical axis in gray scale, with lighter cells indicating fewer rank-switches and darker cells more rank-switches. The lower left triangle within each panel zooms in on the diagonal of the upper right triangle, depicting the proportion of rank-switches between consecutive time periods in a bar chart. We highlight this scenario as it is the most relevant for empirical research on animal personality where repeatability is typically measured across consecutive years. For this portion of the panel, the horizontal axis depicts ontogenetic time periods and the right vertical axis depicts the proportion of rank-switches in that time period.

Diminishing reward & increasing penalties

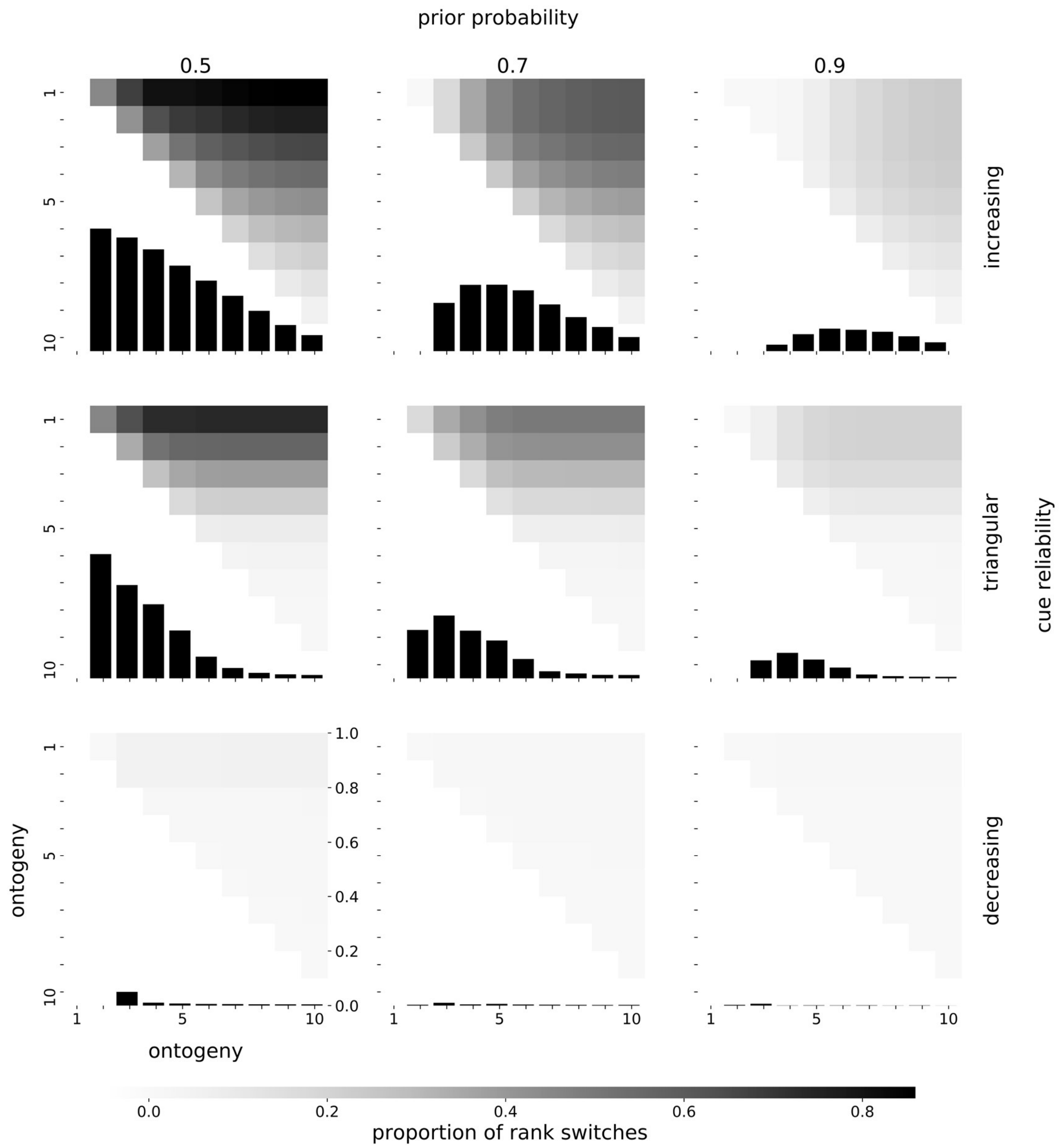

Figure A5.26. Rank-order stability. The fitness rewards for correct specializations are **diminishing** and fitness penalties for incorrect specializations are **increasing** across all panels. The prior probability of E_1 varies across columns and the cue reliability pattern varies across rows. Each panel depicts a simulation of 10,000 organisms following the optimal policy across ontogeny. The environmental state is fixed to E_1. In each time period, organisms are ranked according to the number of specializations towards P_1. Organisms with the same number of specializations share a rank. Each square panel depicts two sets of results, one in the upper right triangle and another in the lower left triangle. For the upper right triangle, the relevant axes are the horizontal and the left vertical, each depicting the full range of ontogenetic time periods. Each cell in this triangle indicates the proportion of rank-switches occurring from the time period on the horizontal axis to the time period on the (left) vertical axis in gray scale, with lighter cells indicating fewer rank-switches and darker cells more rank-switches. The lower left triangle within each panel zooms in on the diagonal of the upper right triangle, depicting the proportion of rank-switches between consecutive time periods in a bar chart. We highlight this scenario as it is the most relevant for empirical research on animal personality where repeatability is typically measured across consecutive years. For this portion of the panel, the horizontal axis depicts ontogenetic time periods and the right vertical axis depicts the proportion of rank-switches in that time period.

Diminishing rewards & diminishing penalties

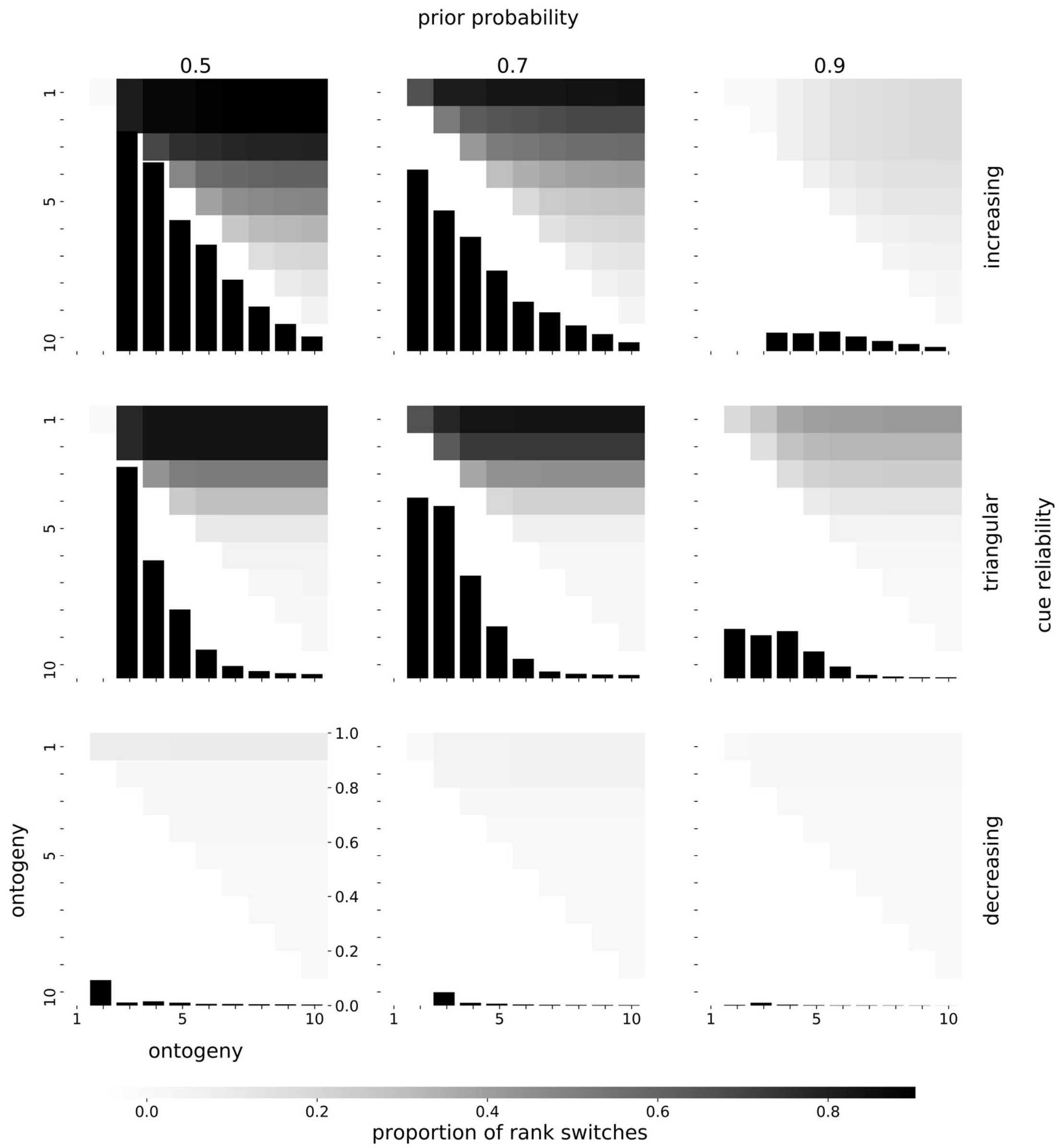

Figure A5.27. Rank-order stability. The fitness rewards for correct specializations are **diminishing** and fitness penalties for incorrect specializations are **diminishing** across all panels. The prior probability of E_1 varies across columns and the cue reliability pattern varies across rows. Each panel depicts a simulation of 10,000 organisms following the optimal policy across ontogeny. The environmental state is fixed to E_1. In each time period, organisms are ranked according to the number of specializations towards P_1. Organisms with the same number of specializations share a rank. Each square panel depicts two sets of results, one in the upper right triangle and another in the lower left triangle. For the upper right triangle, the relevant axes are the horizontal and the left vertical, each depicting the full range of ontogenetic time periods. Each cell in this triangle indicates the proportion of rank-switches occurring from the time period on the horizontal axis to the time period on the (left) vertical axis in gray scale, with lighter cells indicating fewer rank-switches and darker cells more rank-switches. The lower left triangle within each panel zooms in on the diagonal of the upper right triangle, depicting the proportion of rank-switches between consecutive time periods in a bar chart. We highlight this scenario as it is the most relevant for empirical research on animal personality where repeatability is typically measured across consecutive years. For this portion of the panel, the horizontal axis depicts ontogenetic time periods and the right vertical axis depicts the proportion of rank-switches in that time period.

d) Distributions of mature phenotypes

Linear rewards & linear penalties

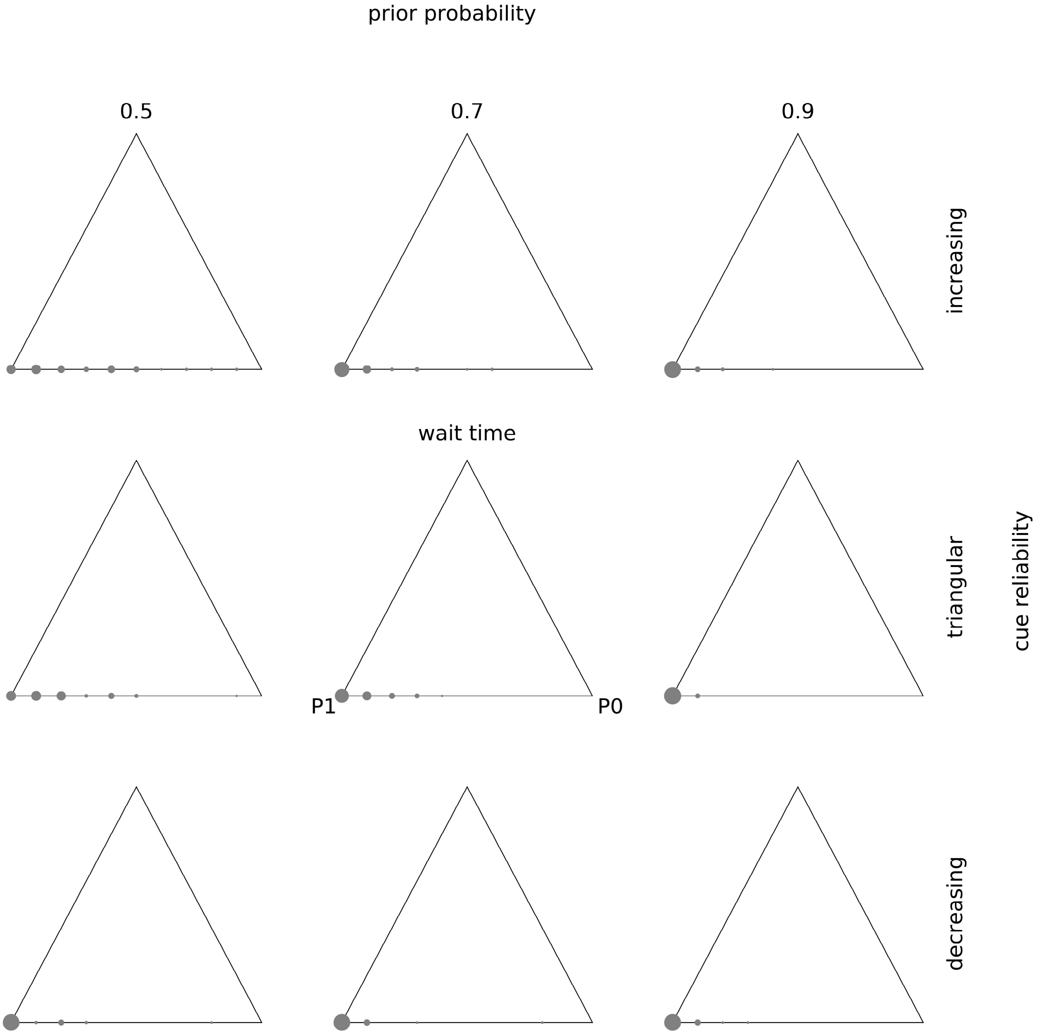

Figure A5.28. Distributions of mature phenotypes. The fitness rewards for correct specializations are **linear** and fitness penalties for incorrect specializations are **linear** across all panels. The prior probability of E_1 varies across columns and the cue reliability pattern varies across rows. Each panel represents a simulation study. For each study we simulate 10,000 organisms who follow the optimal policy and track their development across ontogeny. The environmental state is fixed to E_1. Each triangle plots the distribution of phenotypes at the end of ontogeny. The number of time periods waited, time periods specialized towards P_1 and time periods specialized towards P_0 make up a phenotype. The position of a circle indicates the composition of mature phenotypes. The left and right vertices represent organisms that only specialized towards P_1 and P_0, respectively. The top vertex represents organisms that only waited. Circles on the outer boundary indicate a mixture of two phenotypic decisions, while circles within the triangle indicate a mixture of all three decisions. The area of a circle is proportional to the fraction of simulated organisms that developed the same phenotype.

Linear reward & increasing penalties

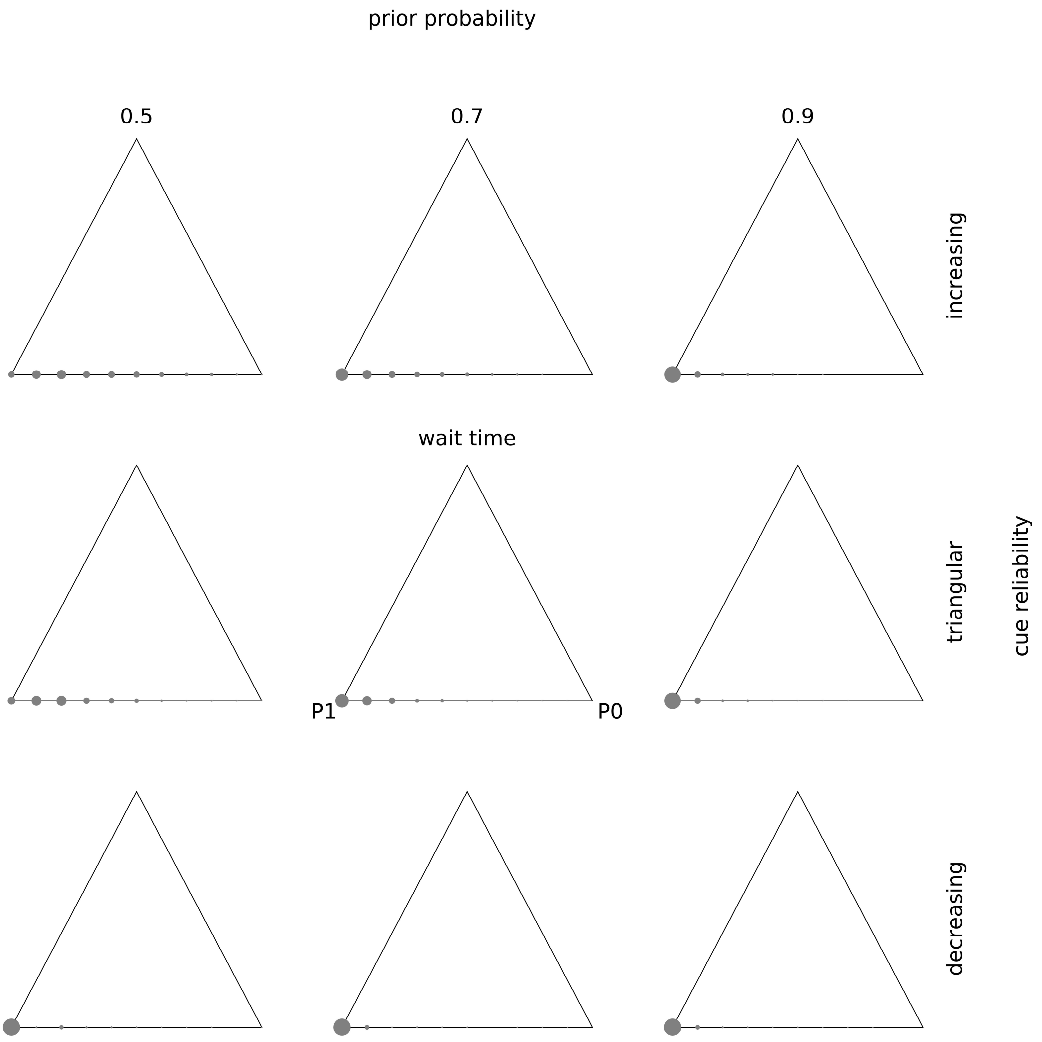

Figure A5.29. Distributions of mature phenotypes. The fitness rewards for correct specializations are **linear** and fitness penalties for incorrect specializations are **increasing** across all panels. The prior probability of E_1 varies across columns and the cue reliability pattern varies across rows. Each panel represents a simulation study. For each study we simulate 10,000 organisms who follow the optimal policy and track their development across ontogeny. The environmental state is fixed to E_1. Each triangle plots the distribution of phenotypes at the end of ontogeny. The number of time periods waited, time periods specialized towards P_1 and time periods specialized towards P_0 make up a phenotype. The position of a circle indicates the composition of mature phenotypes. The left and right vertices represent organisms that only specialized towards P_1 and P_0, respectively. The top vertex represents organisms that only waited. Circles on the outer boundary indicate a mixture of two phenotypic decisions, while circles within the triangle indicate a mixture of all three decisions. The area of a circle is proportional to the fraction of simulated organisms that developed the same phenotype.

Linear rewards & diminishing penalties

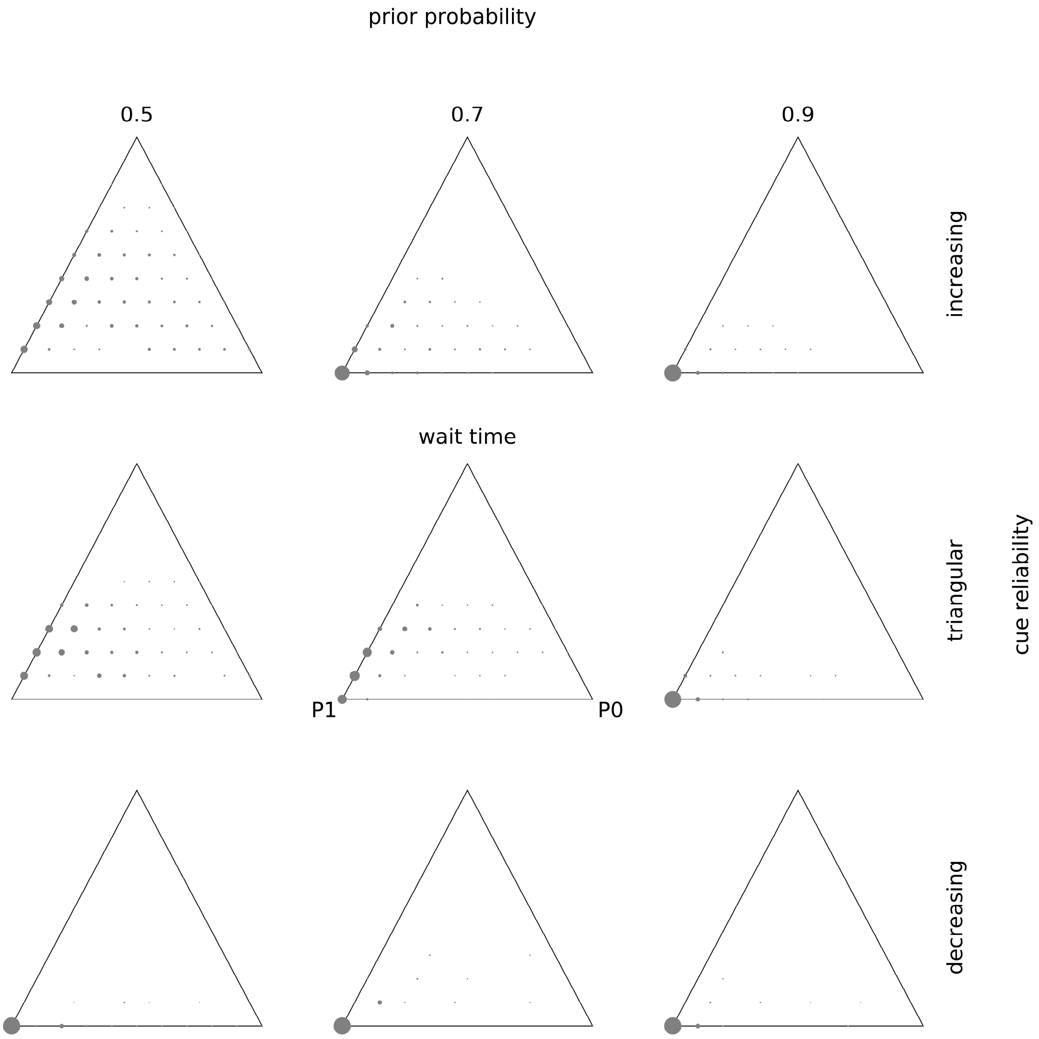

Figure A5.30. Distributions of mature phenotypes. The fitness rewards for correct specializations are **linear** and fitness penalties for incorrect specializations are **diminishing** across all panels. The prior probability of E_1 varies across columns and the cue reliability pattern varies across rows. Each panel represents a simulation study. For each study we simulate 10,000 organisms who follow the optimal policy and track their development across ontogeny. The environmental state is fixed to E_1. Each triangle plots the distribution of phenotypes at the end of ontogeny. The number of time periods waited, time periods specialized towards P_1 and time periods specialized towards P_0 make up a phenotype. The position of a circle indicates the composition of mature phenotypes. The left and right vertices represent organisms that only specialized towards P_1 and P_0, respectively. The top vertex represents organisms that only waited. Circles on the outer boundary indicate a mixture of two phenotypic decisions, while circles within the triangle indicate a mixture of all three decisions. The area of a circle is proportional to the fraction of simulated organisms that developed the same phenotype.

Increasing rewards & linear penalties

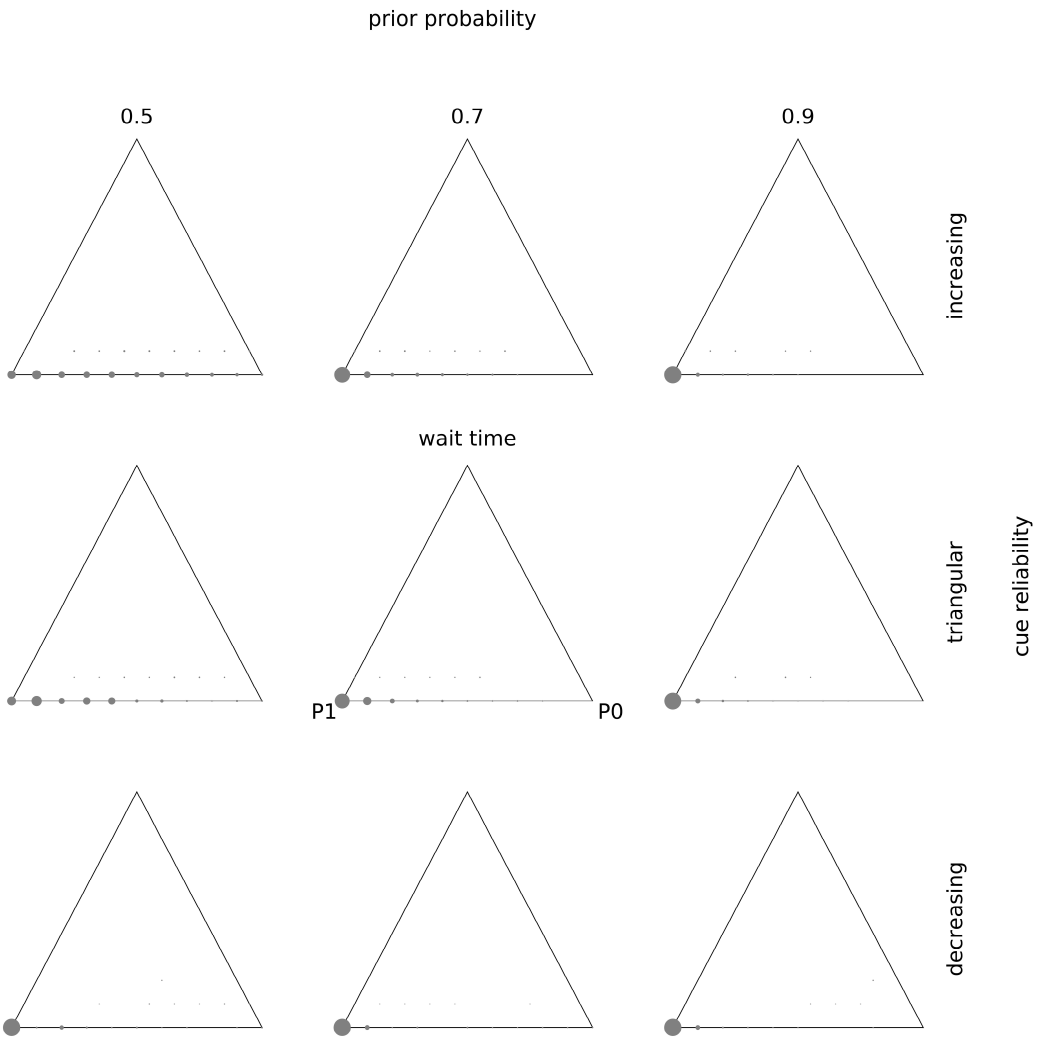

Figure A5.31. Distributions of mature phenotypes. The fitness rewards for correct specializations are **increasing** and fitness penalties for incorrect specializations are **linear** across all panels. The prior probability of E_1 varies across columns and the cue reliability pattern varies across rows. Each panel represents a simulation study. For each study we simulate 10,000 organisms who follow the optimal policy and track their development across ontogeny. The environmental state is fixed to E_1. Each triangle plots the distribution of phenotypes at the end of ontogeny. The number of time periods waited, time periods specialized towards P_1 and time periods specialized towards P_0 make up a phenotype. The position of a circle indicates the composition of mature phenotypes. The left and right vertices represent organisms that only specialized towards P_1 and P_0, respectively. The top vertex represents organisms that only waited. Circles on the outer boundary indicate a mixture of two phenotypic decisions, while circles within the triangle indicate a mixture of all three decisions. The area of a circle is proportional to the fraction of simulated organisms that developed the same phenotype.

Increasing reward & increasing penalties

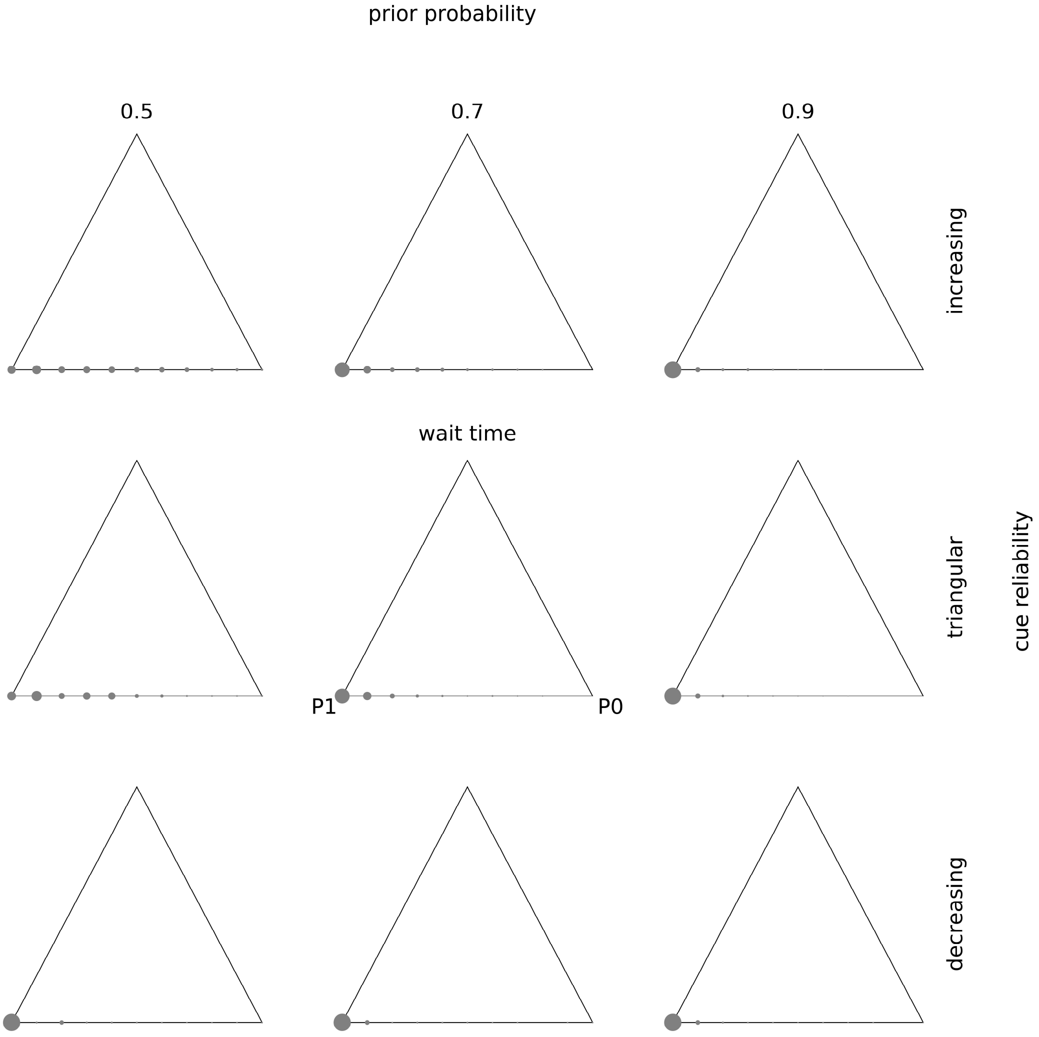

Figure A5.32. Distributions of mature phenotypes. The fitness rewards for correct specializations are **increasing** and fitness penalties for incorrect specializations are **increasing** across all panels. The prior probability of E_1 varies across columns and the cue reliability pattern varies across rows. Each panel represents a simulation study. For each study we simulate 10,000 organisms who follow the optimal policy and track their development across ontogeny. The environmental state is fixed to E_1. Each triangle plots the distribution of phenotypes at the end of ontogeny. The number of time periods waited, time periods specialized towards P_1 and time periods specialized towards P_0 make up a phenotype. The position of a circle indicates the composition of mature phenotypes. The left and right vertices represent organisms that only specialized towards P_1 and P_0, respectively. The top vertex represents organisms that only waited. Circles on the outer boundary indicate a mixture of two phenotypic decisions, while circles within the triangle indicate a mixture of all three decisions. The area of a circle is proportional to the fraction of simulated organisms that developed the same phenotype.

Increasing rewards & diminishing penalties

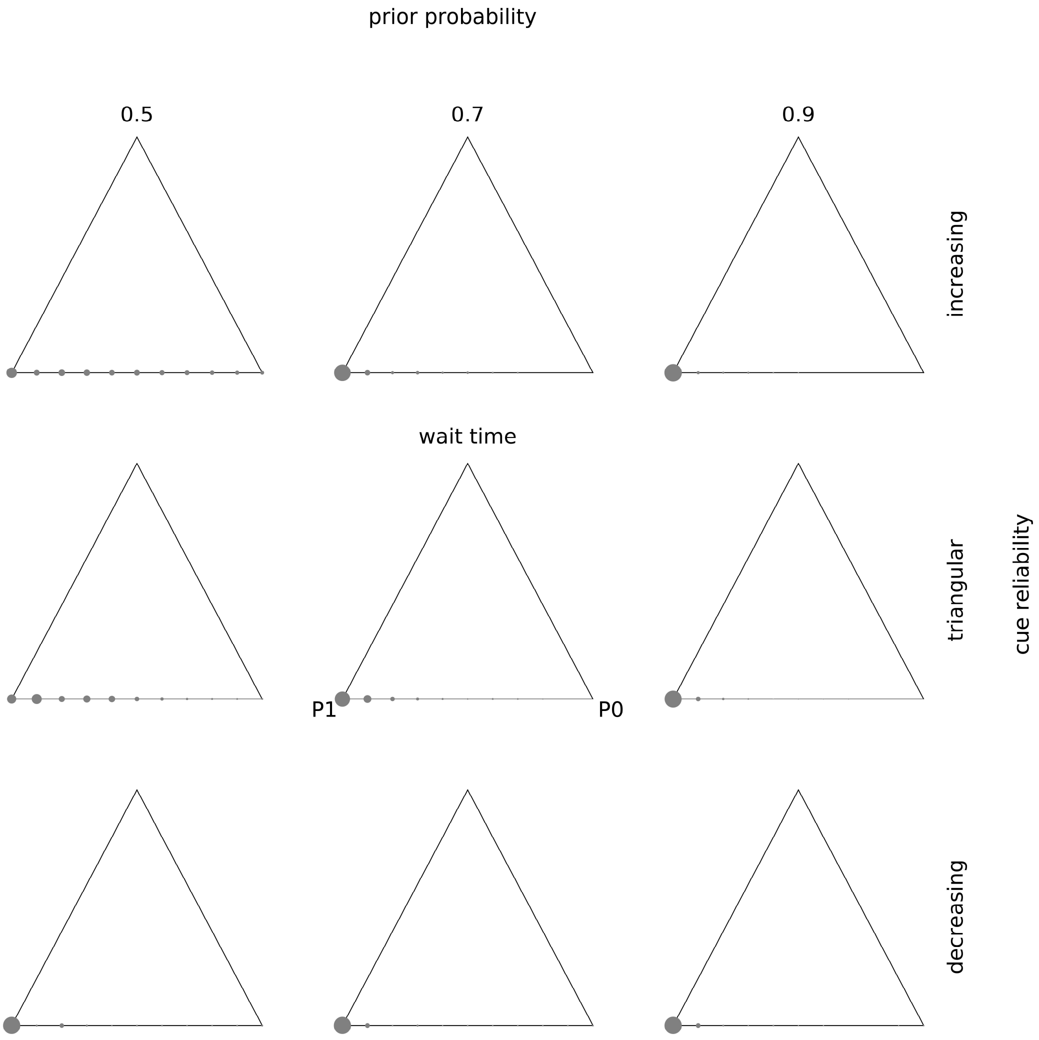

Figure A5.33. Distributions of mature phenotypes. The fitness rewards for correct specializations are **increasing** and fitness penalties for incorrect specializations are **diminishing** across all panels. The prior probability of E_1 varies across columns and the cue reliability pattern varies across rows. Each panel represents a simulation study. For each study we simulate 10,000 organisms who follow the optimal policy and track their development across ontogeny. The environmental state is fixed to E_1. Each triangle plots the distribution of phenotypes at the end of ontogeny. The number of time periods waited, time periods specialized towards P_1 and time periods specialized towards P_0 make up a phenotype. The position of a circle indicates the composition of mature phenotypes. The left and right vertices represent organisms that only specialized towards P_1 and P_0, respectively. The top vertex represents organisms that only waited. Circles on the outer boundary indicate a mixture of two phenotypic decisions, while circles within the triangle indicate a mixture of all three decisions. The area of a circle is proportional to the fraction of simulated organisms that developed the same phenotype.

Diminishing rewards & linear penalties

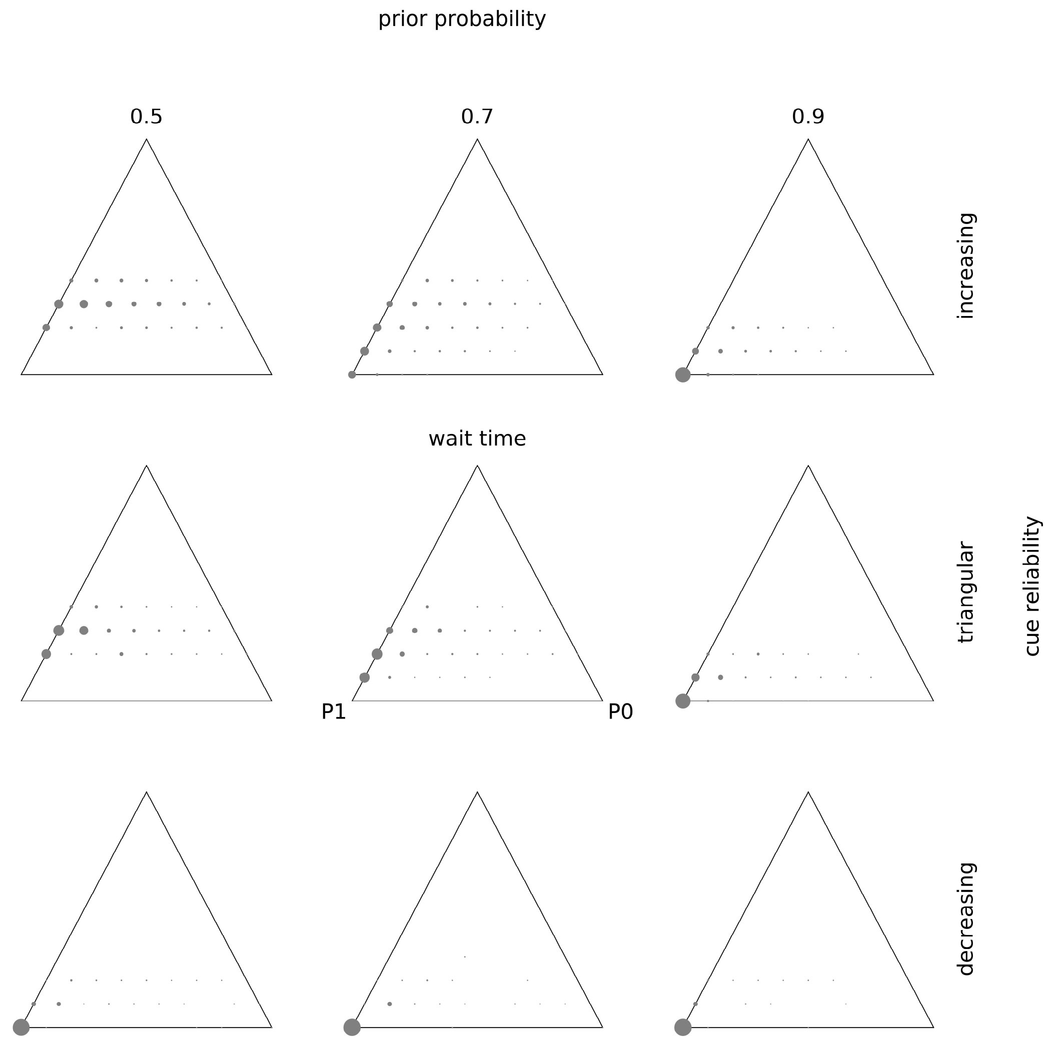

Figure A5.34. Distributions of mature phenotypes. The fitness rewards for correct specializations are **diminishing** and fitness penalties for incorrect specializations are **linear** across all panels. The prior probability of E_1 varies across columns and the cue reliability pattern varies across rows. Each panel represents a simulation study. For each study we simulate 10,000 organisms who follow the optimal policy and track their development across ontogeny. The environmental state is fixed to E_1. Each triangle plots the distribution of phenotypes at the end of ontogeny. The number of time periods waited, time periods specialized towards P_1 and time periods specialized towards P_0 make up a phenotype. The position of a circle indicates the composition of mature phenotypes. The left and right vertices represent organisms that only specialized towards P_1 and P_0, respectively. The top vertex represents organisms that only waited. Circles on the outer boundary indicate a mixture of two phenotypic decisions, while circles within the triangle indicate a mixture of all three decisions. The area of a circle is proportional to the fraction of simulated organisms that developed the same phenotype.

Diminishing reward & increasing penalties

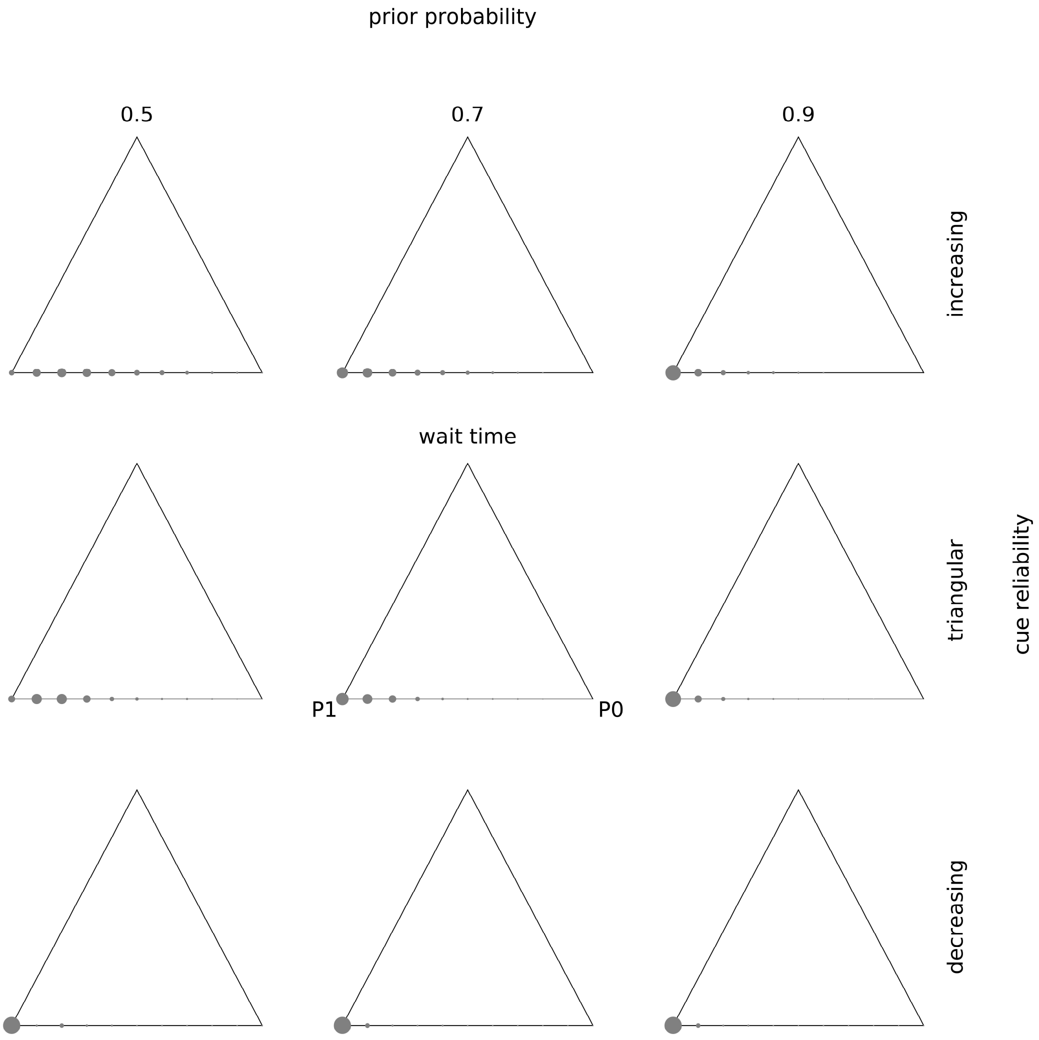

Figure A5.35. Distributions of mature phenotypes. The fitness rewards for correct specializations are **diminishing** and fitness penalties for incorrect specializations are **increasing** across all panels. The prior probability of E_1 varies across columns and the cue reliability pattern varies across rows. Each panel represents a simulation study. For each study we simulate 10,000 organisms who follow the optimal policy and track their development across ontogeny. The environmental state is fixed to E_1. Each triangle plots the distribution of phenotypes at the end of ontogeny. The number of time periods waited, time periods specialized towards P_1 and time periods specialized towards P_0 make up a phenotype. The position of a circle indicates the composition of mature phenotypes. The left and right vertices represent organisms that only specialized towards P_1 and P_0, respectively. The top vertex represents organisms that only waited. Circles on the outer boundary indicate a mixture of two phenotypic decisions, while circles within the triangle indicate a mixture of all three decisions. The area of a circle is proportional to the fraction of simulated organisms that developed the same phenotype.

Diminishing rewards & diminishing penalties

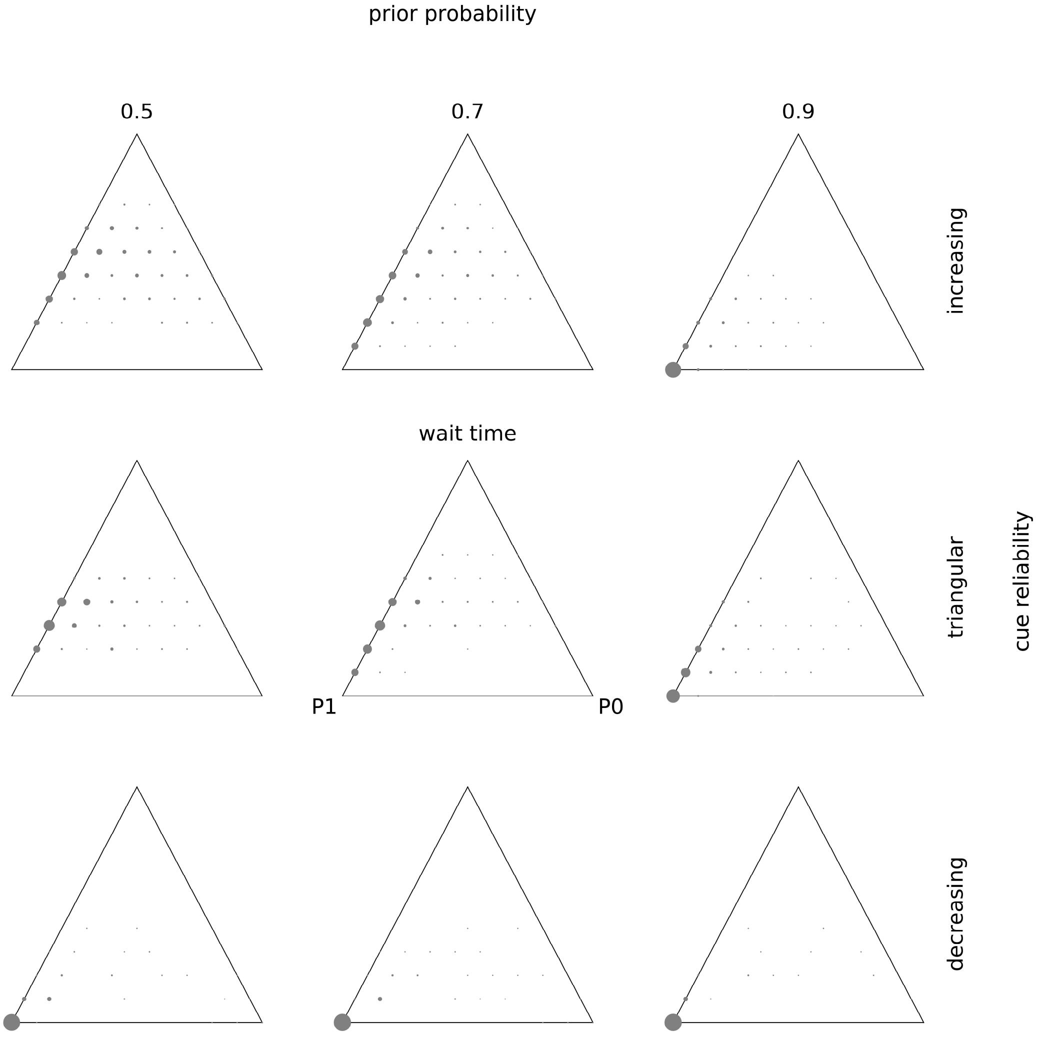

Figure A5.36. Distributions of mature phenotypes. The fitness rewards for correct specializations are **diminishing** and fitness penalties for incorrect specializations are **diminishing** across all panels. The prior probability of E_1 varies across columns and the cue reliability pattern varies across rows. Each panel represents a simulation study. For each study we simulate 10,000 organisms who follow the optimal policy and track their development across ontogeny. The environmental state is fixed to E_1. Each triangle plots the distribution of phenotypes at the end of ontogeny. The number of time periods waited, time periods specialized towards P_1 and time periods specialized towards P_0 make up a phenotype. The position of a circle indicates the composition of mature phenotypes. The left and right vertices represent organisms that only specialized towards P_1 and P_0, respectively. The top vertex represents organisms that only waited. Circles on the outer boundary indicate a mixture of two phenotypic decisions, while circles within the triangle indicate a mixture of all three decisions. The area of a circle is proportional to the fraction of simulated organisms that developed the same phenotype.

e) Fitness of mature phenotypes

Linear rewards & linear penalties

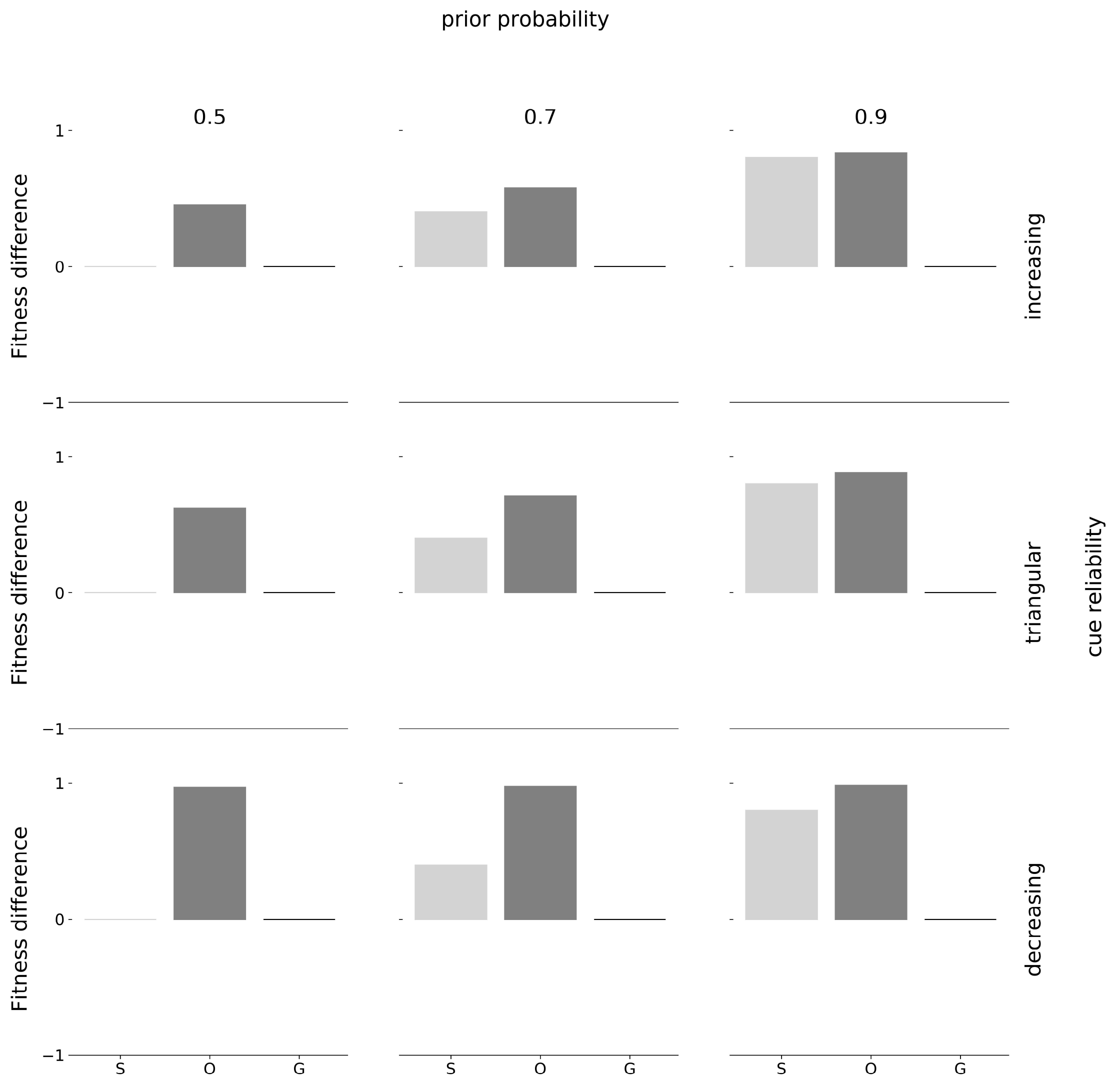

Figure A5.37. Fitness of mature phenotypes. The fitness rewards for correct specializations are **linear** and fitness penalties for incorrect specializations are **linear** across all panels. The prior probability of E_1 varies across columns and the cue reliability pattern varies across rows. Each panel represents a simulation study. For each study we simulate 10,000 organisms who follow the optimal policy and track their development across ontogeny. For half of the population the environmental state is fixed to E_0 and for the other half to E_1 (5,000 organisms per environment).We then compare the average fitness across organisms following the optimal policy ('O'; center, dark-grey bar) to two non-plastic strategies: generalists ('G'; right, black bar) and specialists ('S'; left, light-grey bar). Generalists always specialize halfway towards each phenotypic target, while specialists specialize towards the phenotypic target that is more likely according to the prior. If the prior is 0.5 organisms choose a target at random. Bars indicate the expected fitness difference from baseline (marked as 0) of the three strategies, normalized to range between -1 and 1 with 1 indicating a perfect match to the environment. Fitness differences can be negative when mismatch penalties exceed rewards for correct matches.

Linear reward & increasing penalties

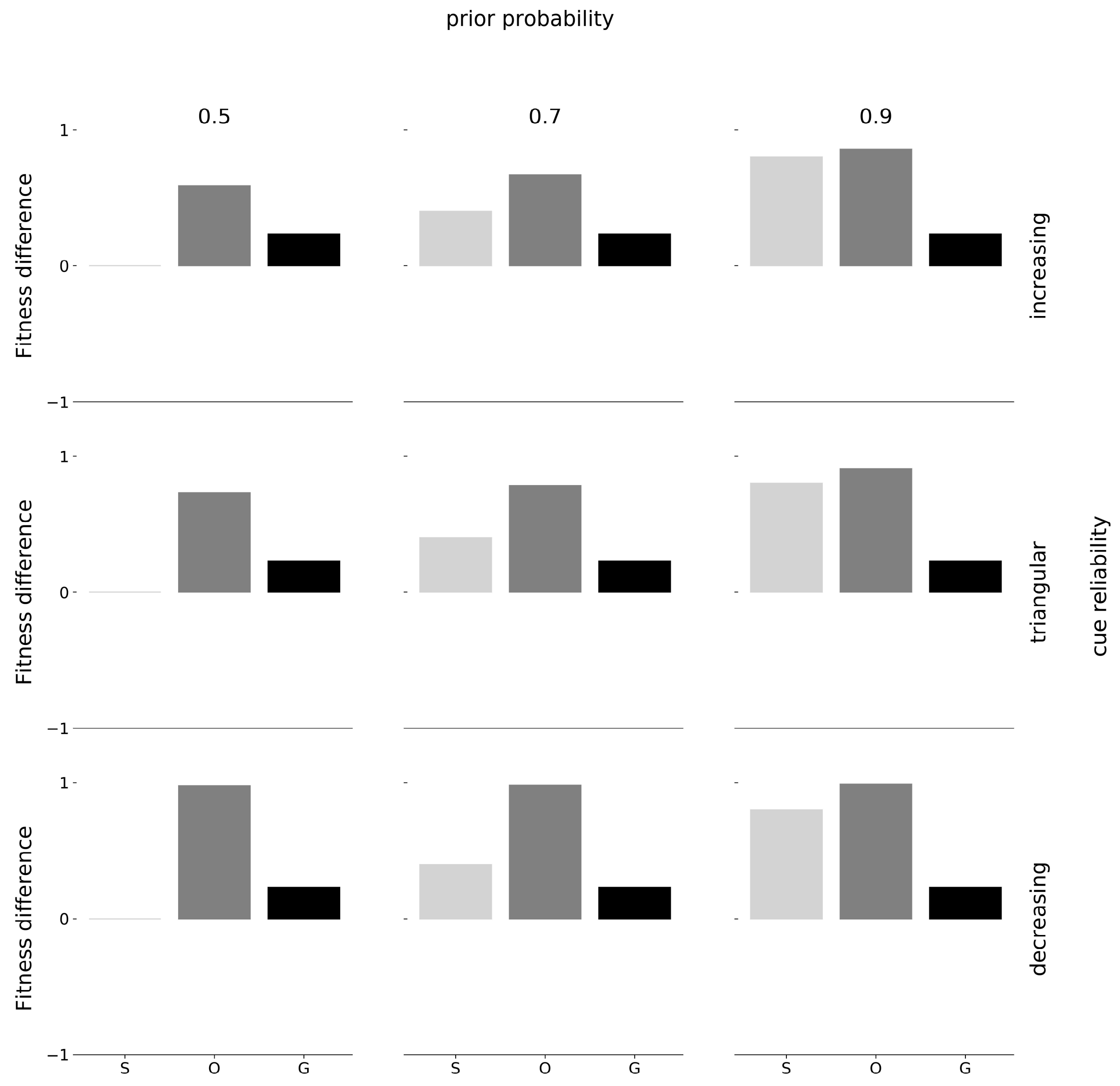

Figure A5.38. Fitness of mature phenotypes. The fitness rewards for correct specializations are **linear** and fitness penalties for incorrect specializations are **increasing** across all panels. The prior probability of E_1 varies across columns and the cue reliability pattern varies across rows. Each panel represents a simulation study. For each study we simulate 10,000 organisms who follow the optimal policy and track their development across ontogeny. For half of the population the environmental state is fixed to E_0 and for the other half to E_1 (5,000 organisms per environment).We then compare the average fitness across organisms following the optimal policy ('O'; center, dark-grey bar) to two non-plastic strategies: generalists ('G'; right, black bar) and specialists ('S'; left, light-grey bar). Generalists always specialize halfway towards each phenotypic target, while specialists specialize towards the phenotypic target that is more likely according to the prior. If the prior is 0.5 organisms choose a target at random. Bars indicate the expected fitness difference from baseline (marked as 0) of the three strategies, normalized to range between -1 and 1 with 1 indicating a perfect match to the environment. Fitness differences can be negative when mismatch penalties exceed rewards for correct matches.

Linear rewards & diminishing penalties

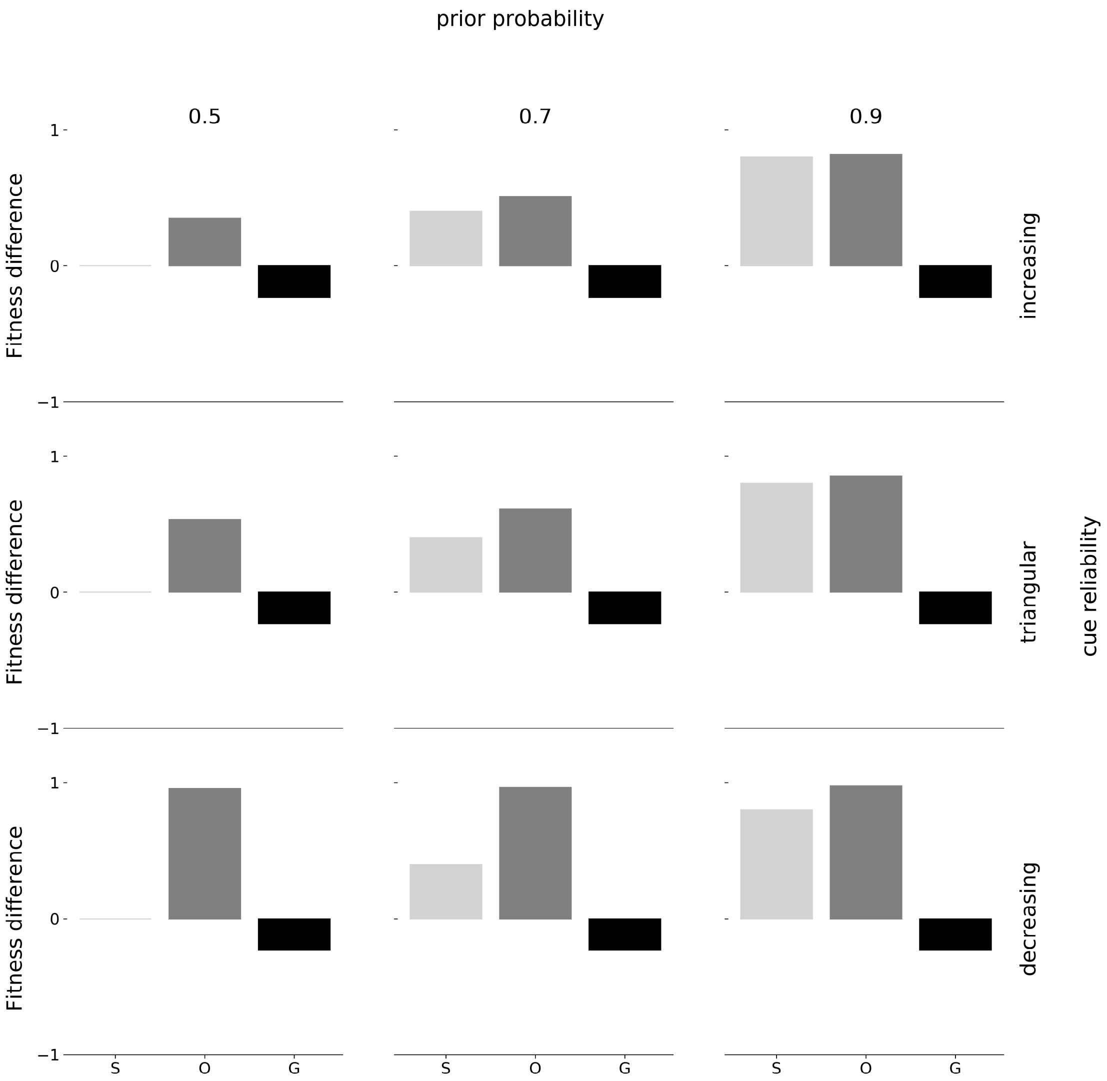

Figure A5.39. Fitness of mature phenotypes. The fitness rewards for correct specializations are **linear** and fitness penalties for incorrect specializations are **diminishing** across all panels. The prior probability of E_1 varies across columns and the cue reliability pattern varies across rows. Each panel represents a simulation study. For each study we simulate 10,000 organisms who follow the optimal policy and track their development across ontogeny. For half of the population the environmental state is fixed to E_0 and for the other half to E_1 (5,000 organisms per environment).We then compare the average fitness across organisms following the optimal policy ('O'; center, dark-grey bar) to two non-plastic strategies: generalists ('G'; right, black bar) and specialists ('S'; left, light-grey bar). Generalists always specialize halfway towards each phenotypic target, while specialists specialize towards the phenotypic target that is more likely according to the prior. If the prior is 0.5 organisms choose a target at random. Bars indicate the expected fitness difference from baseline (marked as 0) of the three strategies, normalized to range between -1 and 1 with 1 indicating a perfect match to the environment. Fitness differences can be negative when mismatch penalties exceed rewards for correct matches.

Increasing rewards & linear penalties

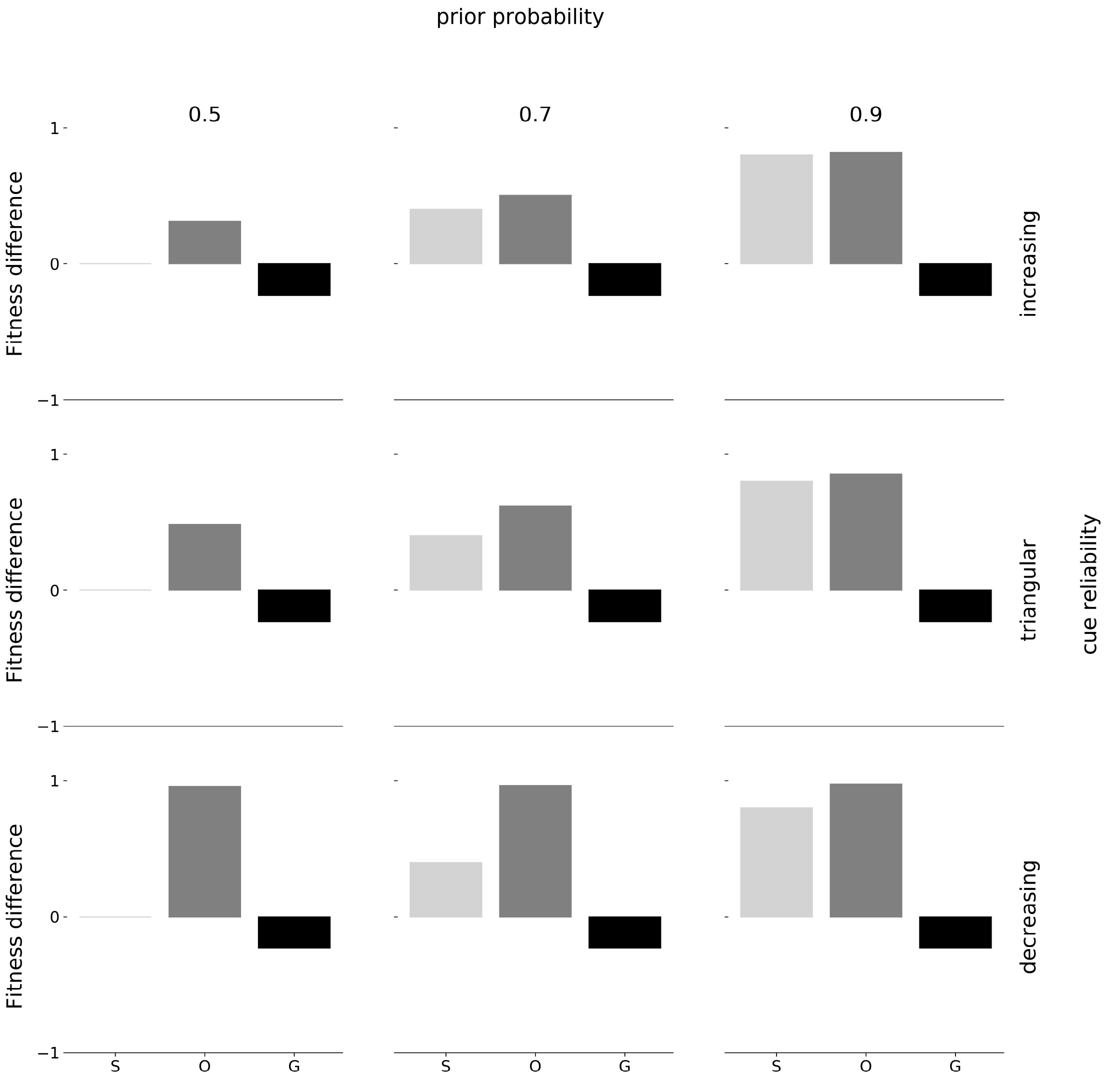

Figure A5.40. Fitness of mature phenotypes. The fitness rewards for correct specializations are **increasing** and fitness penalties for incorrect specializations are **linear** across all panels. The prior probability of E_1 varies across columns and the cue reliability pattern varies across rows. Each panel represents a simulation study. For each study we simulate 10,000 organisms who follow the optimal policy and track their development across ontogeny. For half of the population the environmental state is fixed to E_0 and for the other half to E_1 (5,000 organisms per environment).We then compare the average fitness across organisms following the optimal policy ('O'; center, dark-grey bar) to two non-plastic strategies: generalists ('G'; right, black bar) and specialists ('S'; left, light-grey bar). Generalists always specialize halfway towards each phenotypic target, while specialists specialize towards the phenotypic target that is more likely according to the prior. If the prior is 0.5 organisms choose a target at random. Bars indicate the expected fitness difference from baseline (marked as 0) of the three strategies, normalized to range between -1 and 1 with 1 indicating a perfect match to the environment. Fitness differences can be negative when mismatch penalties exceed rewards for correct matches.

Increasing reward & increasing penalties

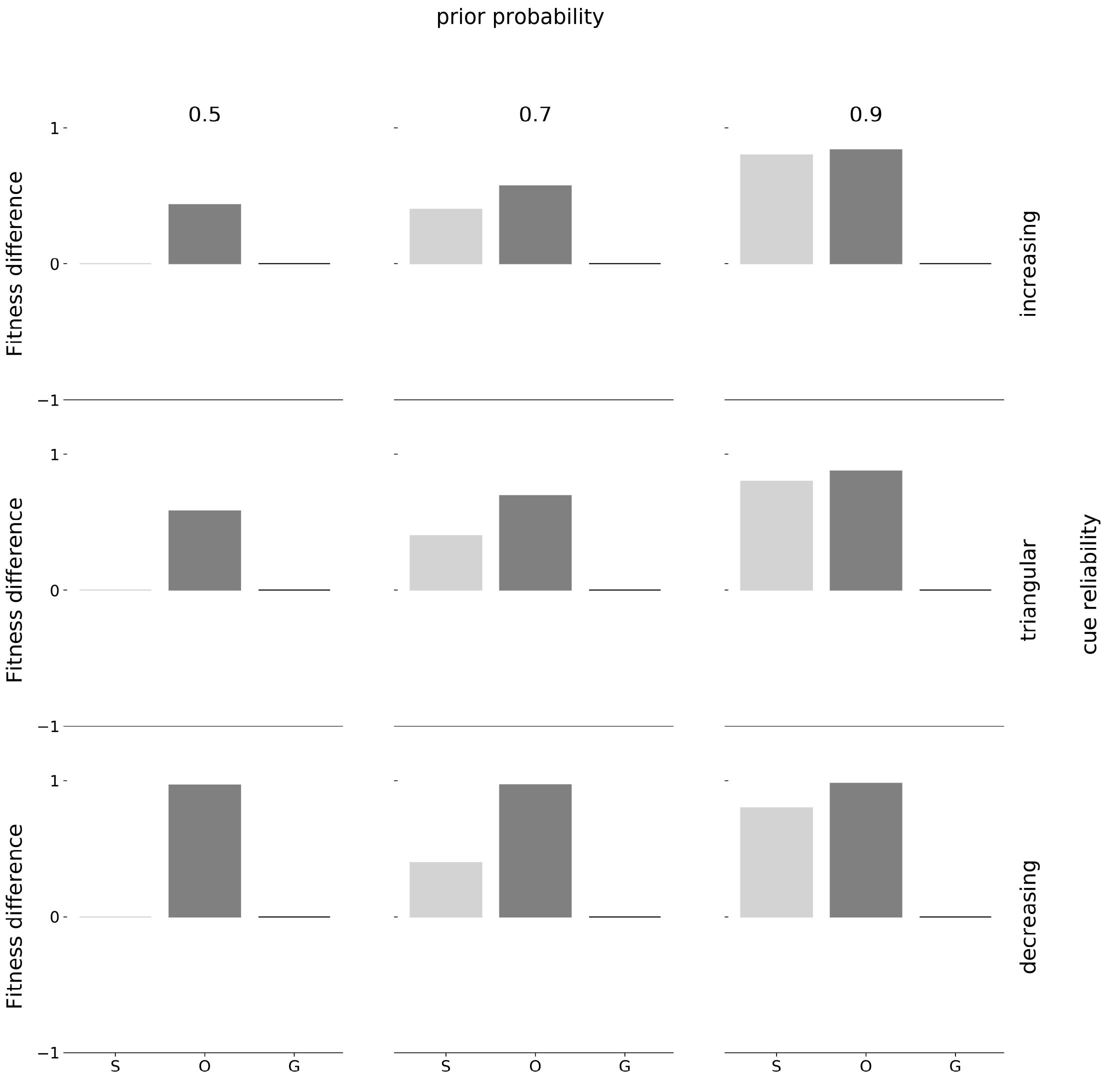

Figure A5.41. Fitness of mature phenotypes. The fitness rewards for correct specializations are **increasing** and fitness penalties for incorrect specializations are **increasing** across all panels. The prior probability of E_1 varies across columns and the cue reliability pattern varies across rows. Each panel represents a simulation study. For each study we simulate 10,000 organisms who follow the optimal policy and track their development across ontogeny. For half of the population the environmental state is fixed to E_0 and for the other half to E_1 (5,000 organisms per environment).We then compare the average fitness across organisms following the optimal policy ('O'; center, dark-grey bar) to two non-plastic strategies: generalists ('G'; right, black bar) and specialists ('S'; left, light-grey bar). Generalists always specialize halfway towards each phenotypic target, while specialists specialize towards the phenotypic target that is more likely according to the prior. If the prior is 0.5 organisms choose a target at random. Bars indicate the expected fitness difference from baseline (marked as 0) of the three strategies, normalized to range between -1 and 1 with 1 indicating a perfect match to the environment. Fitness differences can be negative when mismatch penalties exceed rewards for correct matches.

Increasing rewards & diminishing penalties

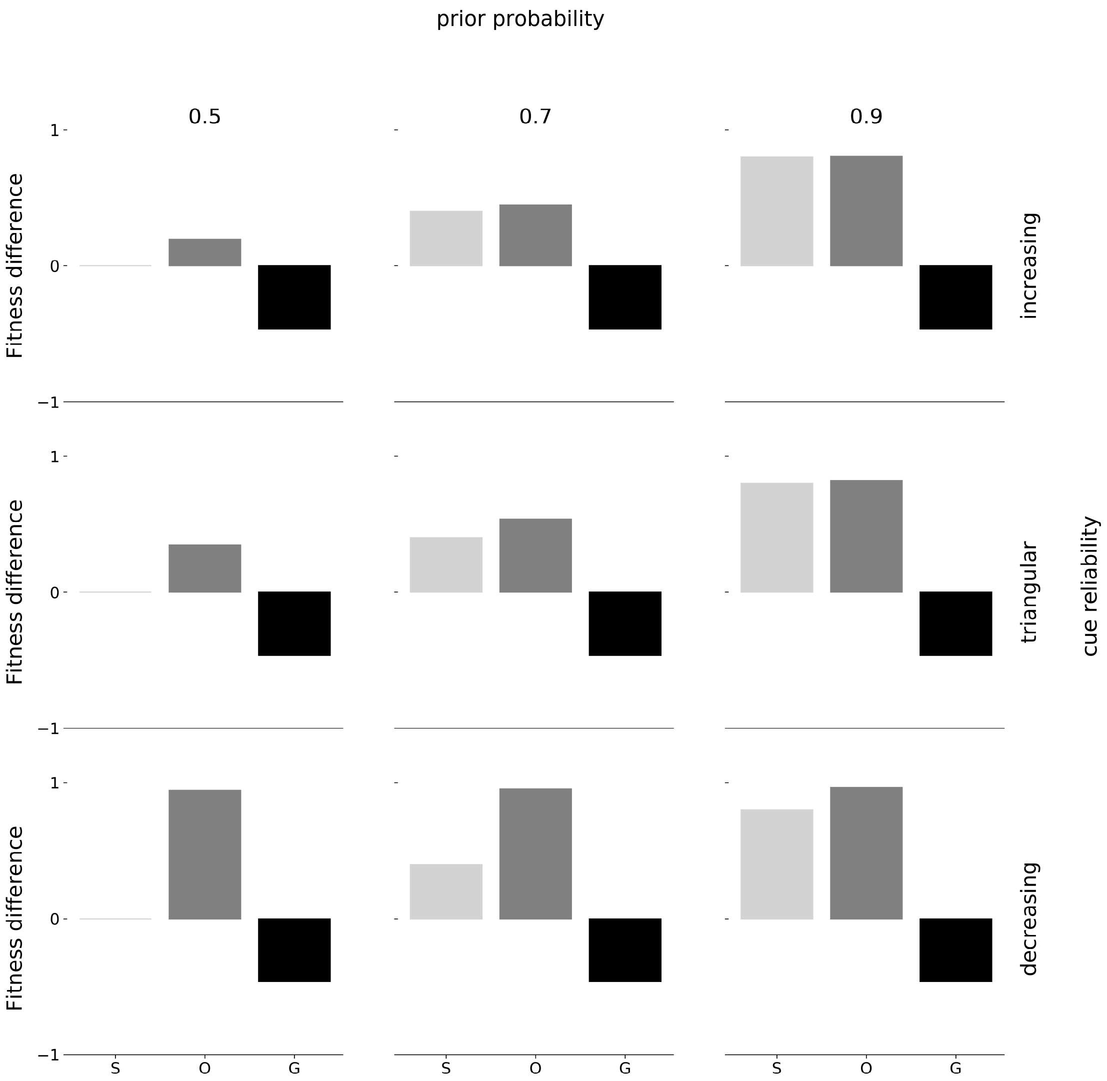

Figure A5.42. Fitness of mature phenotypes. The fitness rewards for correct specializations are **increasing** and fitness penalties for incorrect specializations are **diminishing** across all panels. The prior probability of E_1 varies across columns and the cue reliability pattern varies across rows. Each panel represents a simulation study. For each study we simulate 10,000 organisms who follow the optimal policy and track their development across ontogeny. For half of the population the environmental state is fixed to E_0 and for the other half to E_1 (5,000 organisms per environment).We then compare the average fitness across organisms following the optimal policy ('O'; center, dark-grey bar) to two non-plastic strategies: generalists ('G'; right, black bar) and specialists ('S'; left, light-grey bar). Generalists always specialize halfway towards each phenotypic target, while specialists specialize towards the phenotypic target that is more likely according to the prior. If the prior is 0.5 organisms choose a target at random. Bars indicate the expected fitness difference from baseline (marked as 0) of the three strategies, normalized to range between -1 and 1 with 1 indicating a perfect match to the environment. Fitness differences can be negative when mismatch penalties exceed rewards for correct matches.

Diminishing rewards & linear penalties

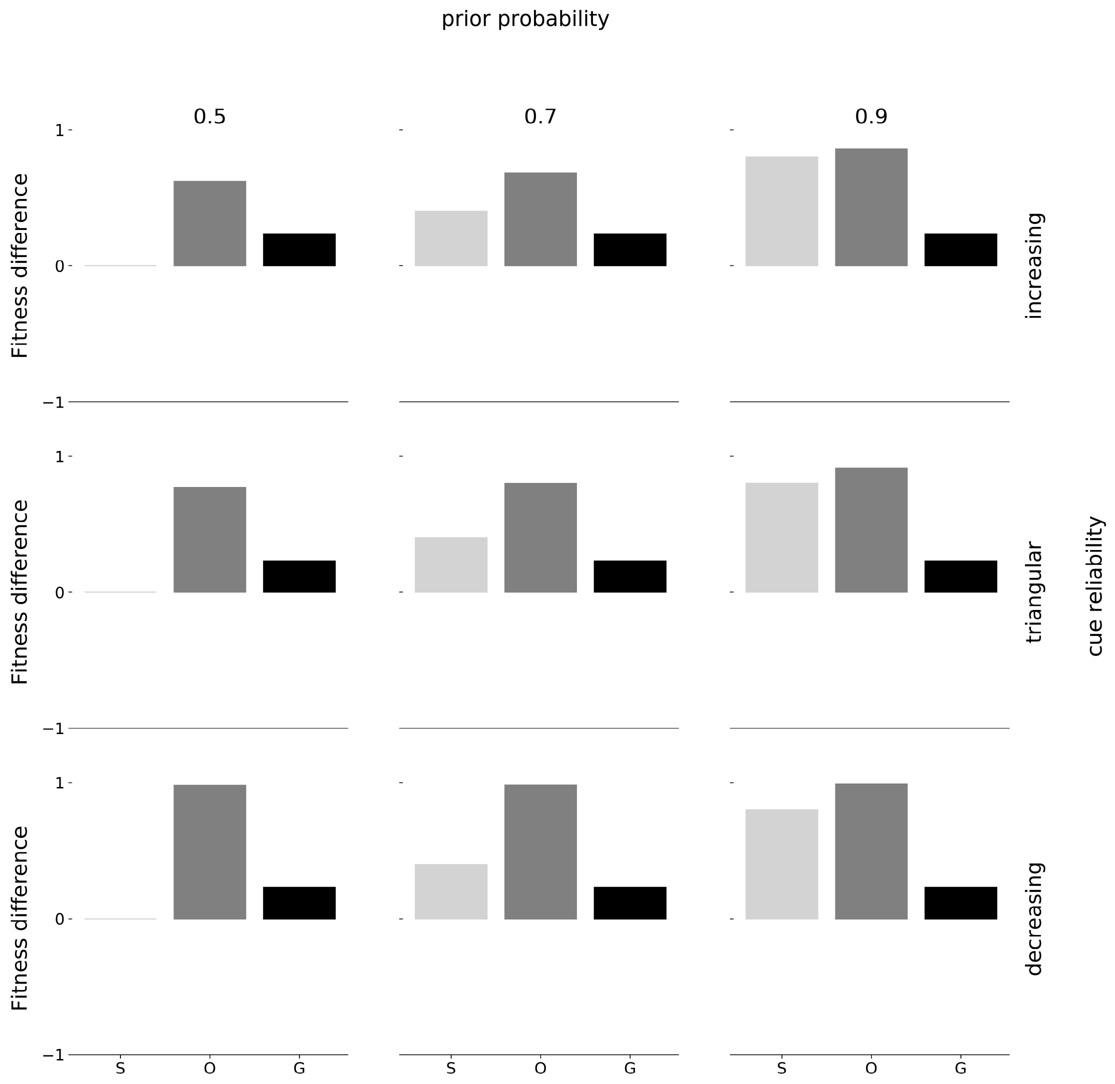

Figure A5.43. Fitness of mature phenotypes. The fitness rewards for correct specializations are **diminishing** and fitness penalties for incorrect specializations are **linear** across all panels. The prior probability of E_1 varies across columns and the cue reliability pattern varies across rows. Each panel represents a simulation study. For each study we simulate 10,000 organisms who follow the optimal policy and track their development across ontogeny. For half of the population the environmental state is fixed to E_0 and for the other half to E_1 (5,000 organisms per environment).We then compare the average fitness across organisms following the optimal policy ('O'; center, dark-grey bar) to two non-plastic strategies: generalists ('G'; right, black bar) and specialists ('S'; left, light-grey bar). Generalists always specialize halfway towards each phenotypic target, while specialists specialize towards the phenotypic target that is more likely according to the prior. If the prior is 0.5 organisms choose a target at random. Bars indicate the expected fitness difference from baseline (marked as 0) of the three strategies, normalized to range between -1 and 1 with 1 indicating a perfect match to the environment. Fitness differences can be negative when mismatch penalties exceed rewards for correct matches.

Diminishing reward & increasing penalties

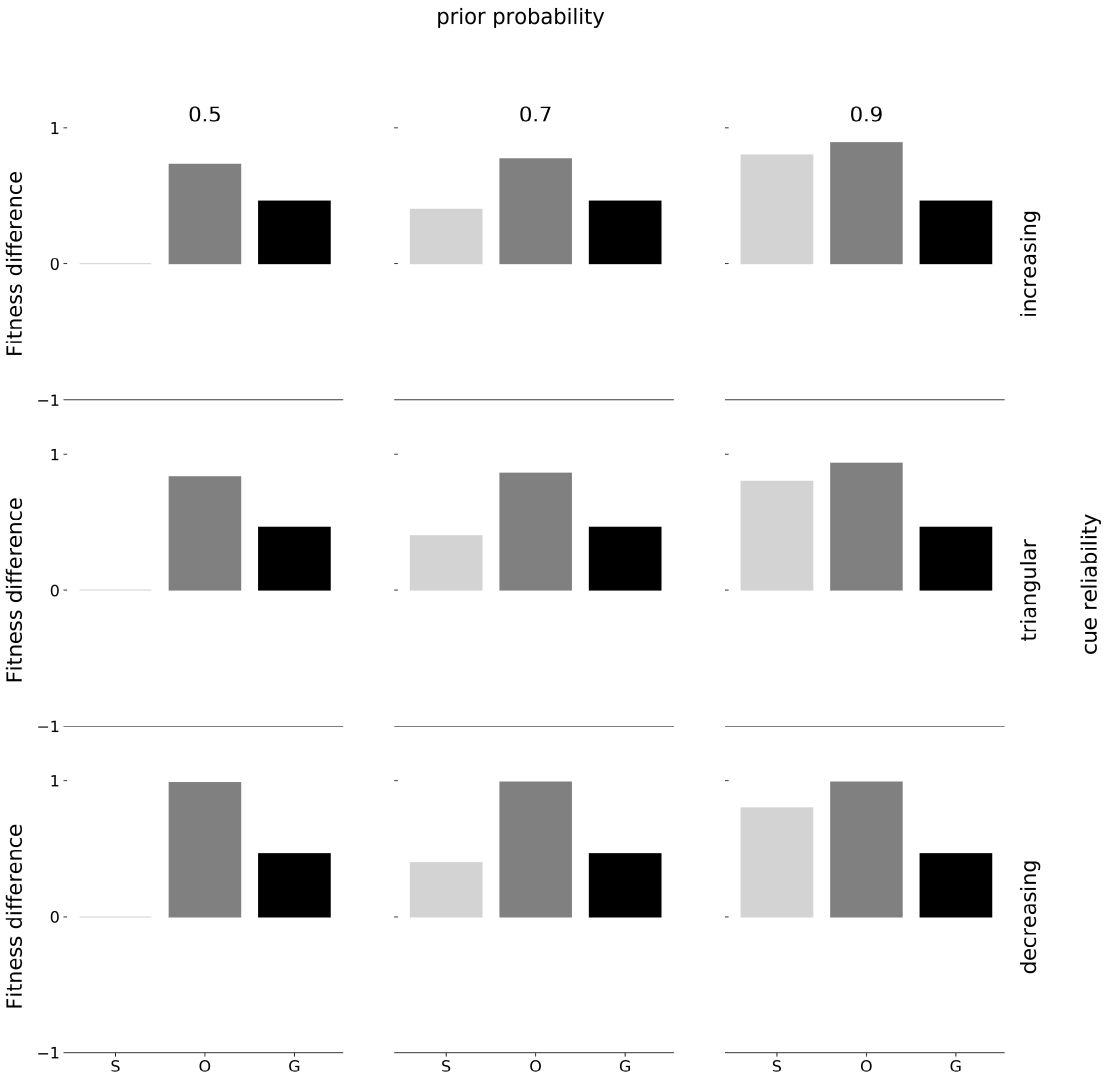

Figure A5.44. Fitness of mature phenotypes. The fitness rewards for correct specializations are **diminishing** and fitness penalties for incorrect specializations are **increasing** across all panels. The prior probability of E_1 varies across columns and the cue reliability pattern varies across rows. Each panel represents a simulation study. For each study we simulate 10,000 organisms who follow the optimal policy and track their development across ontogeny. For half of the population the environmental state is fixed to E_0 and for the other half to E_1 (5,000 organisms per environment).We then compare the average fitness across organisms following the optimal policy ('O'; center, dark-grey bar) to two non-plastic strategies: generalists ('G'; right, black bar) and specialists ('S'; left, light-grey bar). Generalists always specialize halfway towards each phenotypic target, while specialists specialize towards the phenotypic target that is more likely according to the prior. If the prior is 0.5 organisms choose a target at random. Bars indicate the expected fitness difference from baseline (marked as 0) of the three strategies, normalized to range between -1 and 1 with 1 indicating a perfect match to the environment. Fitness differences can be negative when mismatch penalties exceed rewards for correct matches.

Diminishing rewards & diminishing penalties

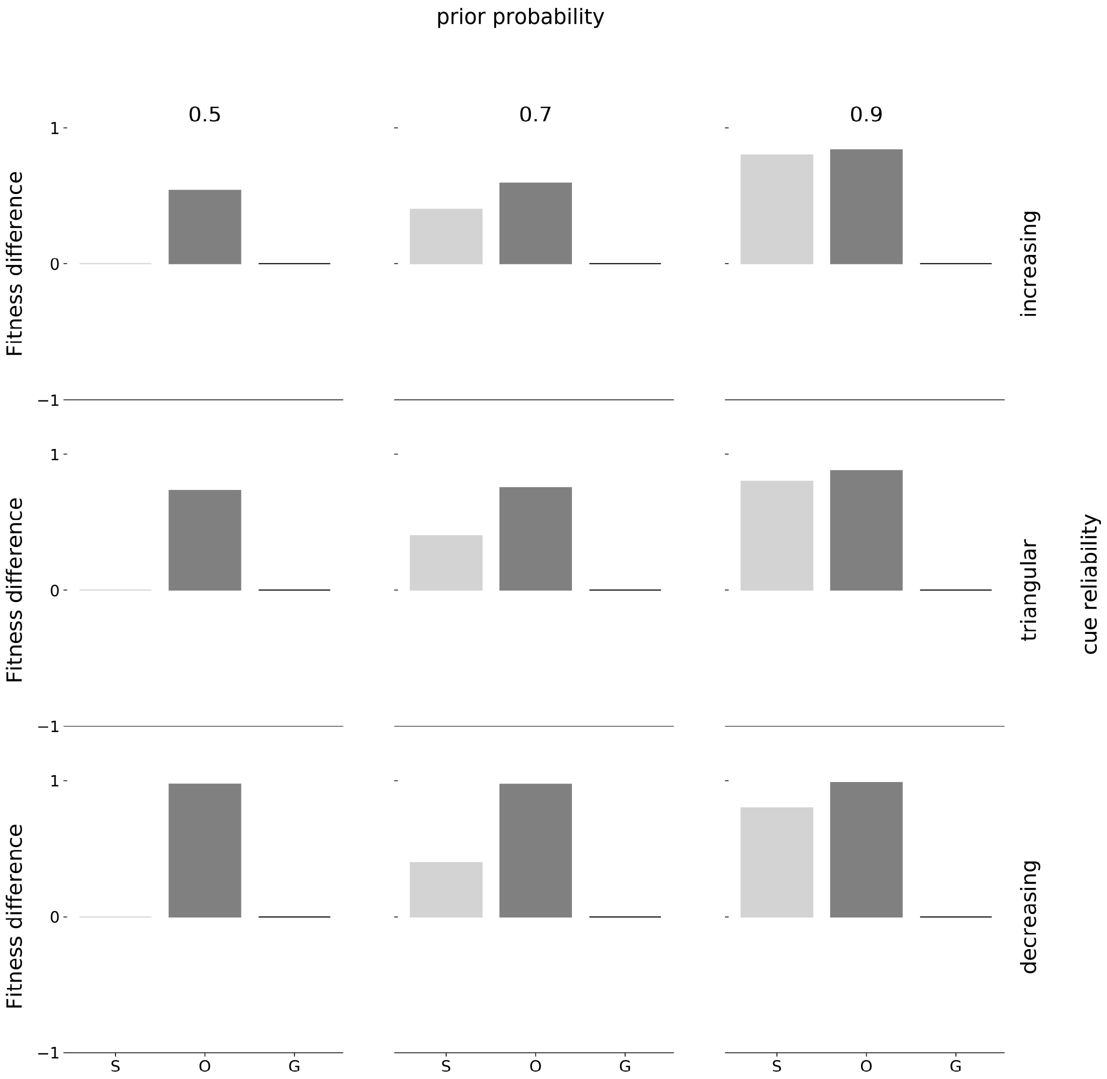

Figure A5.45. Fitness of mature phenotypes. The fitness rewards for correct specializations are **diminishing** and fitness penalties for incorrect specializations are **diminishing** across all panels. The prior probability of E_1 varies across columns and the cue reliability pattern varies across rows. Each panel represents a simulation study. For each study we simulate 10,000 organisms who follow the optimal policy and track their development across ontogeny. For half of the population the environmental state is fixed to E_0 and for the other half to E_1 (5,000 organisms per environment).We then compare the average fitness across organisms following the optimal policy ('O'; center, dark-grey bar) to two non-plastic strategies: generalists ('G'; right, black bar) and specialists ('S'; left, light-grey bar). Generalists always specialize halfway towards each phenotypic target, while specialists specialize towards the phenotypic target that is more likely according to the prior. If the prior is 0.5 organisms choose a target at random. Bars indicate the expected fitness difference from baseline (marked as 0) of the three strategies, normalized to range between -1 and 1 with 1 indicating a perfect match to the environment. Fitness differences can be negative when mismatch penalties exceed rewards for correct matches.
